# Single cell RNA analysis identifies cellular heterogeneity and adaptive responses of the lung at birth

**DOI:** 10.1038/s41467-018-07770-1

**Published:** 2019-01-03

**Authors:** Minzhe Guo, Yina Du, Jason J. Gokey, Samriddha Ray, Sheila M. Bell, Mike Adam, Parvathi Sudha, Anne Karina Perl, Hitesh Deshmukh, S. Steven Potter, Jeffrey A. Whitsett, Yan Xu

**Affiliations:** 1 Division of Pulmonary Biology, Department of Pediatrics, University of Cincinnati College of Medicine, Cincinnati Children’s Hospital Medical Center, Cincinnati, 45229 OH USA; 2 Division of Developmental Biology, Cincinnati Children’s Hospital Medical Center, Cincinnati, 45229 OH USA; 3 Division of Biomedical Informatics, Cincinnati Children’s Hospital Medical Center, Cincinnati, 45229 OH USA

## Abstract

The respiratory system undergoes a diversity of structural, biochemical, and functional changes necessary for adaptation to air breathing at birth. To identify the heterogeneity of pulmonary cell types and dynamic changes in gene expression mediating adaptation to respiration, here we perform single cell RNA analyses of mouse lung on postnatal day 1. Using an iterative cell type identification strategy we unbiasedly identify the heterogeneity of murine pulmonary cell types. We identify distinct populations of epithelial, endothelial, mesenchymal, and immune cells, each containing distinct subpopulations. Furthermore we compare temporal changes in RNA expression patterns before and after birth to identify signaling pathways selectively activated in specific pulmonary cell types, including activation of cell stress and the unfolded protein response during perinatal adaptation of the lung. The present data provide a single cell view of the adaptation to air breathing after birth.

## Abstract (web summary)

The respiratory system is transformed in terms of functional change at birth to adapt to breathing air. Here, the authors examine the molecular changes behind the first breath in the mouse by Drop-seq based RNA sequencing, identifying activation of the unfolded protein response as a perinatal adaptation of the lung.

## Introduction

Adaption of the infant to air breathing is critical to perinatal survival^1,2^. The transition from fetal to postnatal life is mediated by complex physiologic and biochemical processes including ventilation, oxygenation, and increased perfusion of the pulmonary microcirculation^1,3^. Following the first breaths, dynamic structural, biochemical, and functional changes facilitate the transition from a fluid-filled to gas-filled respiratory tract. Multiple cell types, from the conducting airways to peripheral saccules and alveoli, are involved in this critical transition. Alveolar epithelial progenitors differentiate into mature alveolar type 1 (AT1) and type 2 (AT2) cells during the perinatal period. AT1 cells form close contacts with pulmonary endothelial cells lining capillaries, creating the gas exchange region that transports oxygen and carbon dioxide^4^. AT2 cells produce an abundance of surfactant proteins and lipids that reduce surface tension in the alveoli, preventing atelectasis^5^. While the respiratory epithelium actively secretes fluid and electrolytes during fetal life, lung fluids are actively resorbed following birth to establish postnatal ventilation and mucociliary clearance. Apoptosis and inhibition of proliferation of mesenchymal cells causes thinning of alveolar-septal walls, facilitating gas exchange. Vascular, capillary, and lymphatic networks are remodeled, as the microvascular components of the lung expand and mature. Functional changes, including clearance of fetal lung fluid, reduction in pulmonary vascular resistance and enhancement of pulmonary blood flow, and synthesis and release of surfactant occur following birth. Innate and acquired host defense systems are activated, recruiting diverse immune cells to the lung.

Since the respiratory tract matures relatively late in gestation, prematurity underlies the pathogenesis of life-threatening lung disorders, including respiratory distress syndrome (RDS) caused by lack of pulmonary surfactant, and bronchopulmonary dysplasia (BPD), both causing significant morbidity and mortality in premature infants^1,6,7^.

Despite the complexities of lung structure and the diversity of cells involved in lung maturation and adaptation, most genomic and proteomic data used bulk measurements from whole lung tissue to understand perinatal lung development, limiting insights into the activities of and interactions among individual cells^8–11^. Single cell RNA-seq (scRNA-seq) enables transcriptomic mapping of individual cells to measure and understand cellular heterogeneity and responses in complex biological systems^4,12–16^.

Herein, Drop-seq and time course RNA sequencing are used to identify the diversity of pulmonary cells and associated cellular processes activated at birth. A customized analytic pipeline is developed to identify pulmonary cell types and subpopulations as the respiratory tract prepares for and adapts to air breathing. Cell-specific gene signatures, dynamic RNA expression patterns and signaling pathways active at birth are identified. Data from the present study are freely accessed at https://research.cchmc.org/pbge/lunggens/SCLAB.html.

## Results

### The diversity of lung cell types in mouse lung after birth

**Fig. 1:**
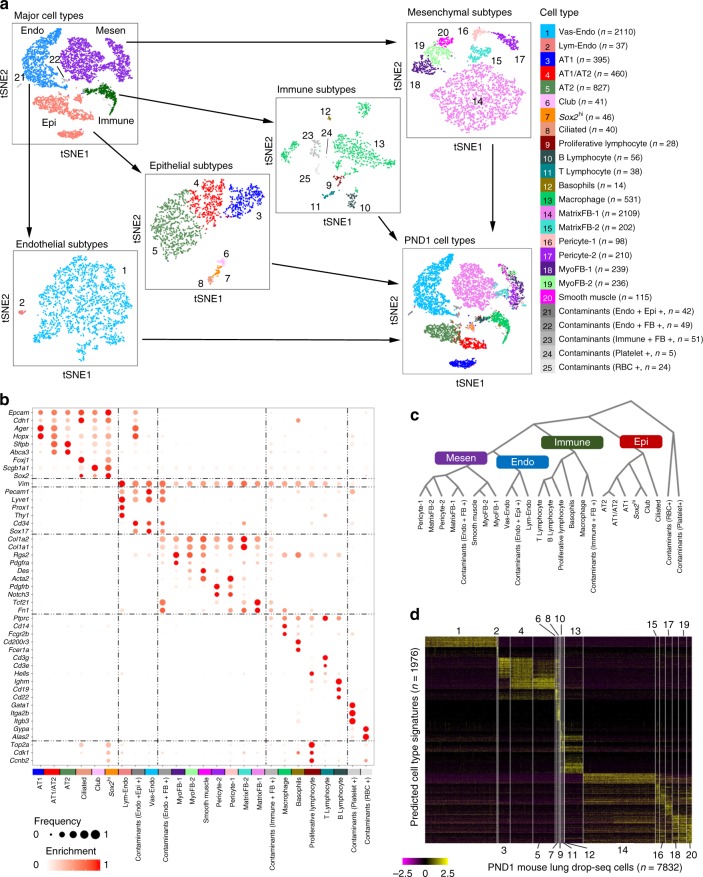
Drop-seq analysis identifies a diversity of cell types in mouse lung after birth. **a** Cell types were identified using an iterative unbiased clustering strategy. Endo endothelial cells, Mesen mesenchymal cells, Immune immune cells, Epi epithelial cells. Cells (*n* = 8003) were from two individual mice at postnatal day 1 (PND1). Source data are provided as Source Data file. **b** Expression of known cell type markers was used to validate the cell type assignments. Node size is proportional to the gene’s expression frequency in a cell type. Node color is proportional to the gene’s sensitivity-based enrichment score in the cluster; red represents high enrichment score; enrichment scores were per gene max normalized for visualization. **c** Hierarchical clustering of cell types was used to reconstruct major lung cell types. Expression of a gene in a cell type was represented by its average expression in all the cells of this type. Pearson’s correlation based on distance and Ward linkage were used. **d** Selective expression of predicted gene signatures in corresponding cell types is shown in the heatmap. The predicted contaminated (doublet) cells (*n* = 171) were not included

Single cell RNA sequencing of whole lung tissue from newborn mice was performed using Drop-seq^13^ (Supplementary Table 1). Data were pre-filtered at both cell and gene level (Methods), resulting in a pool of 8003 cells used for further analysis. Median numbers of genes and transcripts detected per cell were 958 and 1790, respectively, comparable with previous data^17^ (Supplementary Figure 1). Replicates were well correlated after library size normalization (whole genome Pearson’s correlation: 0.98), indicating technical reproducibility of the data. Employing an iterative, graph-based clustering strategy, we identified four major cell types and 20 cell sub-types from postnatal day 1 (PND1) mouse lung (Methods; Fig. 1a; Supplementary Figures 2–6; Supplementary Data 1). Predicted cell types were validated using known cell type selective markers (Fig. 1b). Epithelial cells (*n* = 1809, expressing *Epcam* and *Cdh1*), endothelial cells (*n* = 2147, expressing *Pecam1* and *Emcn*), mesenchymal cells (*n* = 3209, expressing *Col1a1*, *Col1a2* and *Pdgfra*), and immune cells (*n* = 667, expressing myeloid and lymphocyte cell markers, *Ptprc*, *Spi1*, *Cd19*, and *Cd3g*) were identified (Fig. 1; Supplementary Figure 2). Five cell clusters (consisting of 2.1% of cells) expressed markers typical of more than one major cell type (Fig. 1a; Supplementary Figures 2 and 6), supporting the likelihood that they represent doublets. Hierarchical clustering using the expression of predicted signature genes was used to reconstruct major lung cell types from the predicted 20 distinct cell types (Fig. 1c), validating the cell type and subtype assignments. Based on these newly assigned cell types, a binomial probability test^17^ was used to identify differentially expressed genes (false discovery rate adjusted *p*-value < 0.1) and to predict signature genes (Methods; Fig. 1d; Supplementary Table 5; Supplementary Data 2).

### Distinct epithelial cells and their differentiation states

**Fig. 2:**
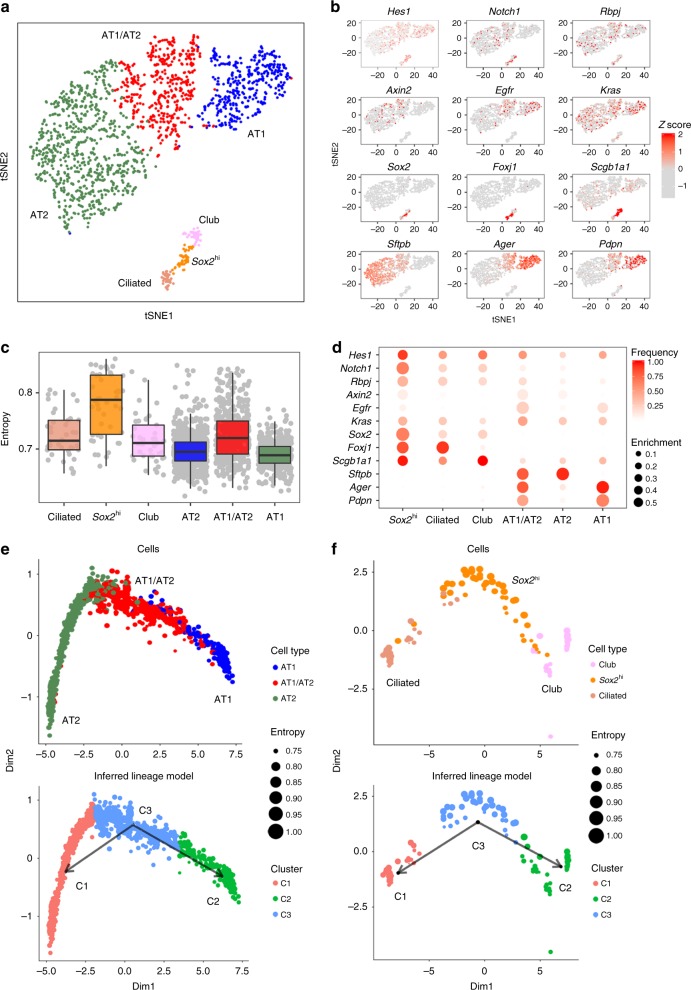
Distinct pulmonary epithelial cells and their differentiation states. **a** Predicted subpopulations of epithelial cells in mouse lung at PND1. **b** Expression of Notch pathway genes (*Hes1*, *Notch1*, and *Rbpj*) and selective markers is shown. The expression is *z*-score normalized. **c** Single cell entropy was used to predict differentiation states of epithelial subpopulations. Entropy of a single cell is calculated using SLICE^19^. Higher entropy represents less differentiated cell states. Boxplots represent 25th (bottom), 50th (centerline), and 75th (top) percentiles. **d** Enrichment of gene expression in epithelial subpopulations. Node size is proportional to the sensitivity-based enrichment score. The scores are per gene max normalized. Node color is proportional to the gene expression frequency in the cluster; red represents high expression frequency. **e** Predicted differentiation lineage model among AT1, AT1/AT2, and AT2 cells. **f** Predicted differentiation lineage model among *Sox2*^hi^, club, and ciliated cells. In **e** and **f**, top panels show cells in reduced dimensional space calculated using the DDRTree method in Monocle 2^18^ and bottom panels show the cell states/clusters and lineage models predicted by SLICE^19^

We identified six distinct epithelial subpopulations, including three peripheral (alveolar) and three proximal (conducting airway) cell types (Fig. 2a; Supplementary Figure 3). Among the three alveolar cell types, AT1 cells expressed *Hopx*, *Pdpn*, *Aqp5*, and *Ager*; AT2 cells expressed *Sftpb*, *Sftpc*, *Abca3*, and *Napsa*. AT1 and AT2 cells did not share signature genes. In contrast, a distinct cluster of cells co-expressed both AT1 and AT2 markers, which we termed “AT1/AT2”, (Fig. 2a, b; Supplementary Figure 3). AT1/AT2 cells co-expressed *Muc1* and *Pdpn*, consistent with characteristics of “bipotent progenitor” cells identified by Desai et al. at E18.5^4,14^. Present data from PND1 lung tissues indicated that AT1/AT2 cells generally lacked *Sox9* RNA and were relatively abundant (25%), compared to bipotent cells at E18.5 (8%)^4,14^. AT1/AT2 cells selectively expressed their own signature genes, including *Egfr* and *Shh*. A subset of AT1/AT2 cells (11%) were *Axin2*^+^ (Fig. 2b), which is more enriched than *Axin2*^+^ in other epithelial cells (two-tailed Chi-square *p*-value < 1e−4), perhaps representing recently reported WNT responsive AT2 progenitor cells^15,16^. Genes associated with EGFR-KRAS signaling, including *Egfr*, *Kras*, *Erbb2*, and *Erbb3*, were selectively enriched in AT1/AT2 cells (Fig. 2b; Supplementary Data 2), supporting the role of EGFR signaling in controlling “bipotent progenitor” cell proliferation and differentiation^4,15^. Notch signaling pathway genes were enriched in AT1/AT2 cells compared to well-differentiated AT1 and AT2 cells (Fig. 2b, d). Proximity ligation fluorescent in situ hybridization (PLISH) identified epithelial cells co-expressing *Sftpc* and *Ager* in peripheral regions of mouse lung (Supplementary Figure 7a, b). In both Drop-seq and PLISH analyses, we identified cells co-expressing *Sftpc* and *Scgb1a1* (Supplementary Figure 7c, d, h); however, these cells were not found preferentially in bronchoalveolar duct junction regions. Using PLISH, we detected six cells co-expressing *Sftpc*, *Ager*, and *Scgb1a1*, representing <0.1% of the lung cells (Supplementary Figure 7e, f). In the Drop-seq data, 20 out of 1809 epithelial cells co-expressed *Sftpc*, *Ager*, and *Scgb1a1* RNA, representing 0.2% of total lung cells in the Drop-seq data; these cells clustered in the AT1/AT2 epithelial subtype (Supplementary Figure 7g).

Among conducting airway epithelial cells, club cells selectively expressed known markers including *Scgb1a1*, *Scgb3a2*, and *Cyp2f2*. Ciliated cell selectively expressed *Foxj1* and a number of dynein encoding genes, *Dnali1*, *Dnah3*, *Dnaic2*, and *Dnaaf1* (Fig. 2; Supplementary Figure 3; Supplementary Data 2). Functional enrichment analysis on predicted subtype signature genes was used to assess the accuracy of the mapping of cell subtypes (Supplementary Data 2). A small group of epithelial cells, which we termed *Sox2*^hi^, expressed both ciliated and club cell markers, as well as high levels of *Sox2* (Fig. 2; Supplementary Figure 3), a transcription factor regulating airway epithelial cell differentiation.

We performed dimension reduction using the DDRTree method in Monocle 2^18^, and then used SLICE^19^ to predict cell differentiation states and differentiation trajectories (Supplementary Figures 8 and 9). SLICE used single cell entropy to predict differentiation states of epithelial subpopulations; experimental validations are needed to confirm the progenitor state. Among the three peripheral cell subtypes, AT1/AT2 cells had the highest entropy, predicting their role as progenitors of AT1 and AT2 cells (Fig. 2c, e; Supplementary Figure 8). Among the three conducting airway subtypes, highest entropy was found in *Sox2*^hi^ cells, supporting their potential role as progenitors of club and ciliated cells (Fig. 2c, f; Supplementary Figure 9). The prediction was consistent with previous studies demonstrating that *Sox2* is required for maintenance and differentiation of bronchiolar club, ciliated, and goblet cells^20,21^. Key components of Notch signaling, *Notch1*, *Rbpj* and *Hes1*, were highly enriched in the *Sox2*^hi^ conducting airway cells and in AT1/AT2 alveolar cells (Fig. 2b, d). *Notch1* and *Hes1* expression varied among three airway cells in the order of *Sox2*^hi^ > club > ciliated, Notch signaling was positively correlated with *Sox2* and negatively correlated with ciliated cell gene expression, consistent with the role of Notch signaling in the regulation of club and ciliated cell differentiation in the conducting airway in part via SOX2^20,22,23^. Notch signaling was more active in *Sox2*^hi^ and AT1/AT2 progenitors and less active in terminally differentiated ciliated and AT1 cells (Fig. 2). Recent single cell transcriptional profiling of human AT2 cell from fibrotic lungs after influenza infection supports a role for Notch signaling in epithelial progenitor proliferation^24^. Taken together, present data support the concept that Notch signaling regulates progenitor cell functions in both conducting and alveolar epithelial cells.

### Endothelial subpopulations and transcriptional mechanisms

**Fig. 3:**
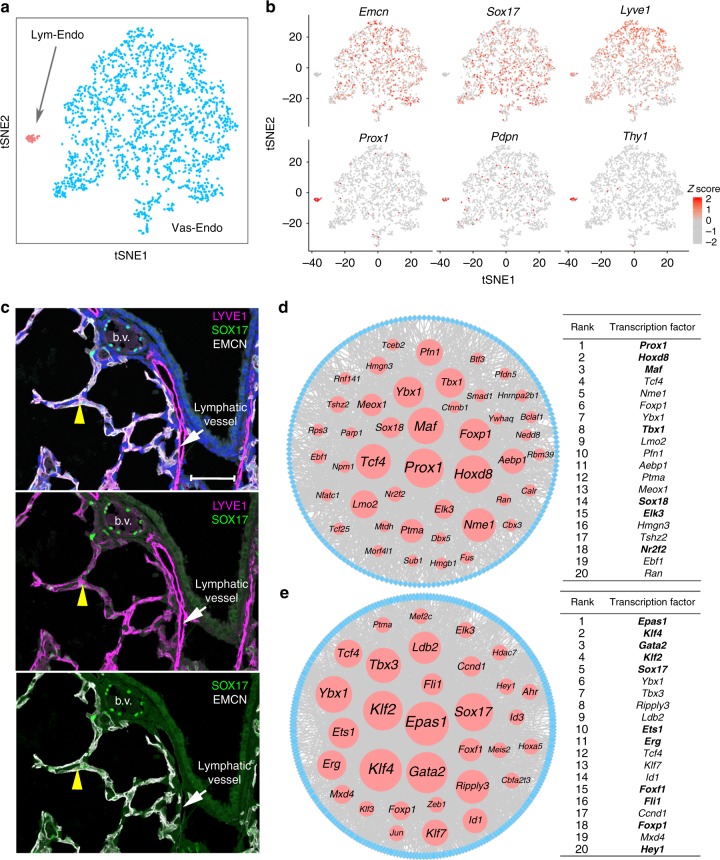
Prediction of key transcription factors for the two endothelial subtypes. **a** Predicted subpopulations of endothelial cells in mouse lung at PND1. **b** Expression of selective lymphatic and vascular endothelial markers is shown. The expression is *z*-score normalized. **c** Immunostaining for LYVE1, SOX17, and EMCN in mouse lung at PND1. LYVE1 is expressed at higher levels in lymphatic vessels (white arrows), and expressed at lower levels in a subset of cells co-expressing SOX17 and/or EMCN. Arrowheads are triple positive cells. Blood vessels (b.v.) are lined with SOX17^+^ cells. Sections from at least three independent animals were evaluated. Scale bar is 50 μm. **d** Prediction of key TFs for the lymphatic endothelial (Lym-Endo) subtype. Left panel shows the predicted TRN for Lym-Endo cells. Right panel shows 20 top-ranked TFs predicted to regulate Lym-Endo subtypes. TFs in bold are known to play important roles in the development of Lym-Endo. **e** Prediction of key transcription factors for the vascular endothelial (Vas-Endo) subtype. Left panel shows the predicted transcriptional regulatory network (TRN) for Vas-Endo cells. Right panel shows 20 top-ranked transcription factors or cofactors (TFs) predicted to regulate the Vas-Endo subtype. TFs in bold are known to play important roles in the development of Vas-Endo. In **d** and **e**, red nodes represent TFs, blue nodes represent target genes (TGs), edges represent the predicted regulatory interactions between TFs and TGs, and node size is proportional to their predicted importance in the reconstructed TRN

Two distinct endothelial cell populations were identified, termed “Vascular Endothelial” (Vas-Endo) and “Lymphatic Endothelial” (Lym-Endo) (Fig. 3a; Supplementary Figure 4). *Pecam1* (CD31), a known pan-endothelial marker, was expressed in both subpopulations. *Emcn*, *Cd34* and *Sox17*, were more abundantly expressed in Vas-Endo (Fig. 3b; Supplementary Figure 4). Signature genes of Vas-Endo cells were enriched for “angiogenesis” and “vascular development” based on functional enrichment analysis using ToppGene^25^ (Supplementary Data 2). *Lyve1*, *Prox1*, *Pdpn*, *Thy1*, and *Flt4*, were co-expressed in Lym-Endo cells (Fig. 3b; Supplementary Figure 4). Signature genes of Lym-Endo were enriched for functional annotations including “regulation of filopodium assembly”, “genes up-regulated in lymphatic endothelial cells compared to blood endothelial cells”, and genes whose deletion or mutation caused “abnormal lymphatic vessel morphology”. Together, these findings support the concept that the Lym-Endo subpopulation represents pulmonary lymphatic endothelial cells, a relatively rare cell type not previously identified by scRNA-seq of mouse lung cells using the Fluidigm C1 platform^26,27^. Our single cell study supports the concept that *Lyve1* is essential, but not sufficient, to identify lymphatic endothelial cells, a finding supported by previous studies^28,29^ and our immunofluorescence co-staining of LYVE1, EMCN and/or SOX17, the latter two selective markers for vascular endothelial cells (Fig. 3c). *Lyve1* may be useful for identification of lymphatic endothelial cells in combination with additional markers, such as *Thy1* or *Prox1*.

We used the driving force analysis in SINCERA^30^ to infer the transcriptional regulatory networks (TRNs) and predict key transcription factors (TFs) regulating lymphatic and vascular endothelial subtypes (Fig. 3d, e; Methods). The predicted 20 most important TFs are shown in Fig. 3. Only three TFs (*Foxp1*, *Ybx1*, and *Tcf4*) were shared (Fig. 3d, e), suggesting that vascular and lymphatic endothelial cell types were regulated by distinct transcriptional programs. A number of predicted TFs known to play important roles in the development of lymphatic endothelial cells (*Prox1*, *Hoxd8*, *Maf*, *Tbx1*, *Sox18*, *Elk3*, and *Nr2f2*^31–34^) and vascular endothelial cells (*Epas1*, *Foxf1*, *Foxp1*, *Klf2*, *Klf4*, *Gata2*, *Sox17*, *Ets1*, *Erg*, *Hey1*, and *Fli1*^34,35^) were identified, supporting the validity of the prediction method. Previously unreported TFs predicted in our analysis represent potential important regulators of lung lymphatic and vascular endothelial cells for experimental validation.

### Diverse pulmonary mesenchymal cells

**Fig. 4:**
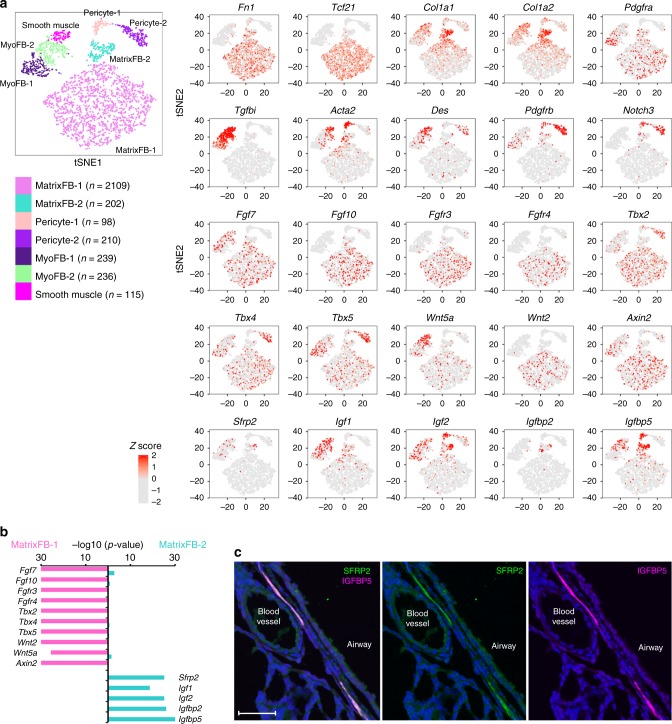
Diverse pulmonary mesenchymal cells and expression of signaling pathway genes. **a** Visualization of the expression of cell type signature genes, T-box transcription factors, and WNT, FGF, and IGF signaling pathway genes are shown in a *t*-distributed stochastic neighbor embedding (tSNE) plot of the seven mesenchymal subtypes. Left panel shows the reference map of seven mesenchymal subtypes in tSNE plot. Right panel shows the expression of signature and signaling pathway genes. **b** Differential expression of T-box transcription factors, WNT, FGF, and IGF pathway genes in the two MatrixFB subtypes is shown. Violet bars: −log_10_ transformed *p-*value of binomial differential expression test of genes in MatrixFB-1 cells; blue bars: −log_10_ transformed *p-*value of binomial differential expression test of genes in MatrixFB-2 cells. Minimum *p-*value was set to 1E−30. **c** Immunofluorescence staining shows co-location of SFRP2 and IGFBP5 in a subset of mesenchymal cells, representing a subset of the MatrixFB-2 cell type. Sections from at least three independent animals were evaluated. Scale bar is 50 μm

Seven mesenchymal cell subtypes, including “Matrix Fibroblast” (MatrixFB, *n* = 2 subtypes), pericyte (*n* = 2), and “Myofibroblast” (MyoFB)/smooth muscle cells (*n* = 3) were identified on the basis of unbiased clustering analysis of the Drop-seq data (Fig. 4a; Supplementary Figure 5). While cell-specific markers were not readily discerned, mesenchymal cell subtypes were largely defined by expression gradients of cell selective markers. MatrixFB-1 cells expressed higher levels of *Tcf21*, *Fn1*, *Fgf10*, and *Vcam1*; MatrixFB-2 cells expressed higher levels of type 1 collagen (*Col1a1* and *Col1a2*) (Fig. 4a). Signature genes of MatrixFB-1 and MatrixFB-2 were largely different but were shared commonly enriched functions, including “extracellular matrix organization” and “collagen formation” (Supplementary Data 2). Both expressed proliferative markers at low levels and had similar levels of single cell entropy, supporting the concept that MatrixFB-1 and MatrixFB-2 likely represent two distinct functional cell subtypes rather than two-cell states.

Potential regulators controlling MatrixFB-1 and MatrixFB-2 cell subtypes were predicted by gene expression analysis. WNT (*Wnt2*, *Wnt5a*, and *Axin2*) and FGF signaling (*Fgf10*, *Fgf7*, *Fgfr3* and *Fgfr4*), as well as T-box TFs (*Tbx2*, *Tbx4* and *Tbx5*) were significantly enriched in MatrixFB-1 cells. *Sfrp2*, an inhibitor of WNT signaling, and a family of insulin-like growth factors and binding proteins ( *Igf1*, *Igf2*, *Igfbp2*, and *Igfbp5*) were enriched in MatrixFB-2 cells (Fig. 4b). Immunofluorescence staining of Fibronectin 1 (FN1), a selective marker for MatrixFB-1, was localized in peribronchiolar and perivascular fibroblasts (Supplementary Figure 10). Immunofluorescence staining demonstrated a subset of MatrixFB-2 cells co-expressing SFRP2 and IGFBP5 within the mesenchymal compartment lining proximal airways (Fig. 4c). These cells were found juxtaposed to cells expressing myoFB/smooth muscle markers, ACTA2 and TGFBI (Supplementary Figures 11 and 12).

Pericytes were subdivided into two sub-populations, Pericyte-1 and Pericyte-2, both expressing multiple pericyte selective markers, including *Pdgfrb*, *Notch3*, *Mcam*, *Cspg4*. Pericyte-1 cells selectively expressed *Map3k7cl*, *Mustn1*, and *Acta2*. Pericyte-2 cells expressed *Agtr1a*, *Vsnl1*, and *Art3*, but lacked or expressed low levels of *Acta2*. PDGFRβ^+^/CSPG4^+^ and PDGFRβ^+^/CSPG4^+^/ACTA2^+^ pericytes were identified by immunofluorescence staining (Supplementary Figure 13).

Three distinct subtypes of smooth muscle/myoFBs were identified, including MyoFB-1, MyoFB-2, and Smooth Muscle. MyoFB-1 cells expressed high levels of *Pdgfra* and *Ednrb*, but lacked mature muscle markers *Actg2*, *Des*, and *Cnn1*. MyoFB-2 cells co-expressed myoFB and smooth muscle markers and may represent cells in transition from myoFBs to smooth muscle cells. Smooth muscle cells expressed smooth muscle markers (*Actg2*, *Cnn1*, and *Des*), but lacked myoFB markers *Pdgfra* and *Ednrb*. WNT signaling genes were highly expressed in the smooth muscle/myoFBs clusters of cells. A subset of MyoFB-1 cells (11%) co-expressed *Axin2* and *Pdgfra*, of which 50% expressed *Fgf7*, perhaps representing a subset of mesenchymal alveolar niche cells located adjacent to AT2 progenitor cells involved in AT2 regeneration^16^. In order to validate MyoFB subtypes, we performed immunofluorescence staining of PDGFRα-GFP^+^ and ACTA2 using a GFP lineage traced mouse model^36^. The majority of PDGFRα-GFP^+^ fibroblasts (MyoFB) detected in terminal saccules lacked FN1 (MatrixFB-1) at E18.5 (Supplementary Figure 10). MyoFB-1 (PDGFRα-GFP^+^/αSMA^−^), MyoFB-2 (PDGFRα-GFP^+^/αSMA^+^), and smooth muscle (PDGFRα-GFP^−^/αSMA^+^) cells were identified (Supplementary Figure 10). In addition, we identified a marker, *Tgfbi*, expressed in all three subtypes of smooth muscle/myoFBs cells in the Drop-seq data. TGFBI^+^/PDGFRβ^+^/ACTA2^+^ myoFBs and TGFBI^+^/ACTA2^+^ smooth muscle cells were identified in mouse lung at PND1 (Supplementary Figure 12).

### Subpopulations of pulmonary immune cells

Five distinct sub-populations of immune cells were identified using unbiased clustering and were further defined based on the expression of signature genes and known immune cell markers (Fig. 1; Supplementary Figure 6). Cells selectively expressing *Fcer1a* and *Cd200r3* were predicted to be basophils; cells selectively expressing *Cd14*, *Fcgr2b*, and *Spi1*, were predicted as a macrophage cell type. Three subtypes of lymphocytes were identified. Cells selectively expressing *Cd19*, *Cd22*, and *Ighm* were defined as B lymphocyte, while those expressing *Cd3g*, *Cd3e*, and *Cd7* were defined as T lymphocyte. A third lymphocytic subtype expressed high levels of cell cycle-associated genes (e.g., *Top2a*, *Mki67*, and *Cdk1*), multiple histone cluster 1 genes and *Hells*, a gene encoding a lymphoid-specific helicase involved in “lymphocyte proliferation”. We designated these cells as proliferative lymphocytes, consistent with known proliferative immune cells in the postnatal lung^37^. Flow cytometry further identified the diversity of immune cell populations, including macrophages, T cells, and B cells in PND1 mouse lungs (Supplementary Figure 14).

### Comparison of Drop-seq and Fluidigm C1 predictions

Drop-seq analysis of PND1 lung cells was validated by an independent scRNA-seq experiment using the Fluidigm C1 platform (Supplementary Table 1; Supplementary Figure 15; Supplementary Data 3 and 4). Total of seven cell type/subtypes were identified by Fluidigm C1 while 20 subtypes were identified by drop-seq (Supplementary Figure 15a, b). Cell types and signature genes identified from Drop-seq and Fluidigm C1 were largely correlated and consistent (Supplementary Figure 15c, d). Larger numbers of cells obtained in the Drop-seq data enabled better resolution of cell type heterogeneity (Supplementary Figure 15b). Fluidigm C1 detected more differentially expressed genes per cell (Supplementary Figure 15d), which may enable better pathway and network analyses, finding consistent with other comparative studies^38,39^.

### Dynamic regulation of genes and pathways at birth

**Fig. 5:**
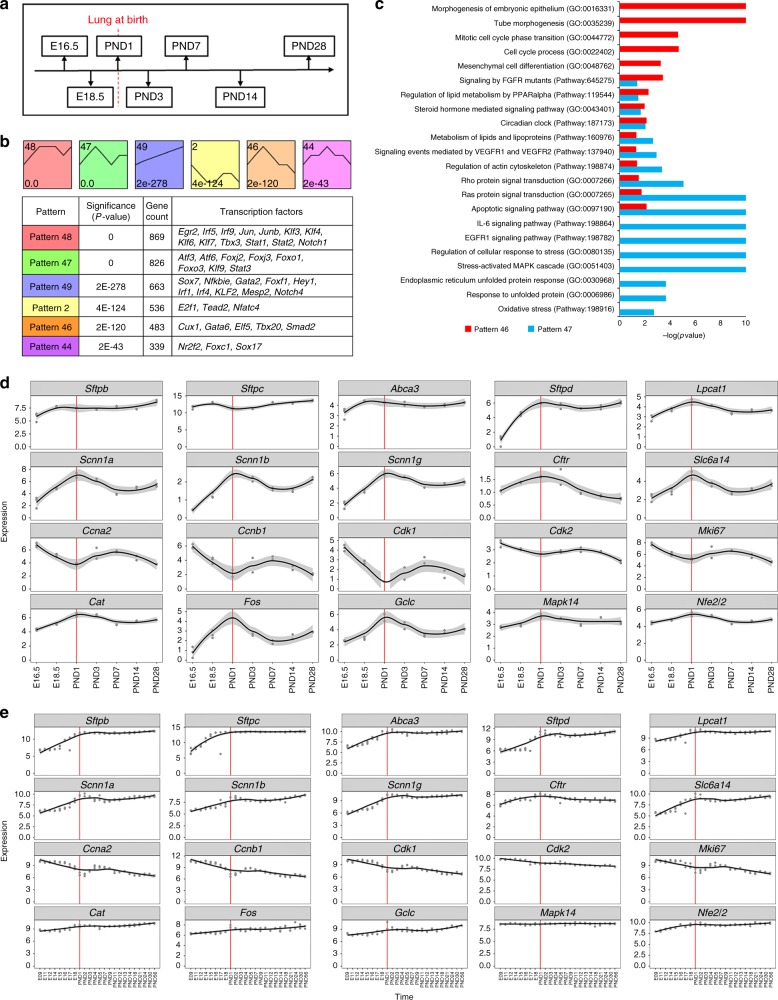
Dynamic regulation of genes and bioprocesses at birth. **a** RNA-seq samples were collected from seven time points of mouse lung development. **b** Top panel: six representative temporal gene expression patterns were identified; red dashed lines indicate PND1. Bottom panel: the significance (*p-*value), gene counts, and transcription factors in the six representative gene expression patterns. **c** Functional annotations uniquely or commonly enriched by the temporal gene expression patterns “Pattern 46” and “Pattern 47”. **d**, **e** The dynamic patterns of representative genes in four biological processes during mouse lung development. Red lines represent the data from mouse lung at PND1. The four biological processes and representative genes include surfactant synthesis (*Sftpb*, *Sftpc*, *Abca3*, *Sftpd*, *Lpcat1*), fluid transport and clearance (*Scnn1a*, *Scnn1b*, *Scnn1g*, *Cftr*, *Slc6a14*), cell proliferation (*Ccna2*, *Ccnb1*, *Cdk1*, *Cdk2*, *Mki67*), and response to oxidative stress (*Cat*, *Fos*, *Gclc*, *Mapk14*, *Nfe2l2*). **d** Gene expression patterns derived from RNA-seq data of mouse lung at E16.5-PND28. **e** Gene expression patterns derived from published RNA microarray data of mouse lung development from E9.5 to PND56^10^. Gray dots represent individual data points. Black lines represent fitted locally weighted scatterplot smoothing profiles; gray regions are the confidence intervals around smoothing

To identify genes and pathways associated with pulmonary maturation, we analyzed RNA-seq experiments from mouse whole lung samples collected at seven developmental time points, embryonic day 16.5 (E16.5), E18.5, postnatal days 1, 3, 7, 14, and 28 (Fig. 5a; Supplementary Table 2). Using short time-series expression miner (STEM)^40^, we identified six major temporal RNA expression patterns (Fig. 5b; Supplementary Data 5) and validated the patterns using a published developmental time-series of mouse lung RNA microarray data, consisting of 26 time points from E9.5 to PND56^10^ (Supplementary Figure 16).

Expression patterns 46 and 47 peaked on PND1, likely representing genes and bioprocesses activated at birth. Pattern 47 (826 genes) was uniquely enriched in stress-related biological processes and signaling pathways, including “stress-activated MAPK cascade”, “regulation of cellular response to stress”, “responded to unfolded protein”, “endoplasmic reticulum unfolded protein response” and “oxidative stress” (Fig. 5c). Pattern 46 (483 genes) decreased after-birth and thereafter. This group of genes shares predicted roles in “mesenchymal cell differentiation”, “mitotic cell cycle phase transition”, and “tube morphogenesis”. The major bioprocesses and pathways shared by Patterns 46 and 47 include “circadian clock” and “metabolism of lipids and lipoproteins” (Fig. 5c). The activation of the circadian clock genes at birth may indicate responses to light. Induction of “metabolism of lipids and lipoproteins” is likely related to the metabolic adaptations needed to provide energy and substrates for surfactant biosynthesis necessary for ventilation^1^. Pattern 49 genes increased expression progressively after birth and were associated with “positive regulation of immune system process” and “immune response” (Supplementary Data 5). Pattern 2 genes were associated with “cell cycle process and phase transition” and “RNA splicing and processing” (Supplementary Data 5); their expression was increased from PND7 to PND14, consistent with increased alveolarization and septal growth (Fig. 5b). Dynamic gene expression analysis of lung tissue RNA-seq data from E16.5 to PND28 identified the induction of a network of genes designed to enhance surfactant synthesis and to establish alveolar fluid balance (Fig. 5d, e), processes required for lung function at birth. Expression of stress-related genes and bioprocesses, including “regulation of cellular response to stress”, “responded to unfolded protein”, “endoplasmic reticulum unfolded protein response”, and “oxidative stress” was increased at birth; in contrast genes involved in cell proliferation were decreased before birth. (Fig. 5).

### Activation of the unfolded protein response pathway at birth

**Fig. 6:**
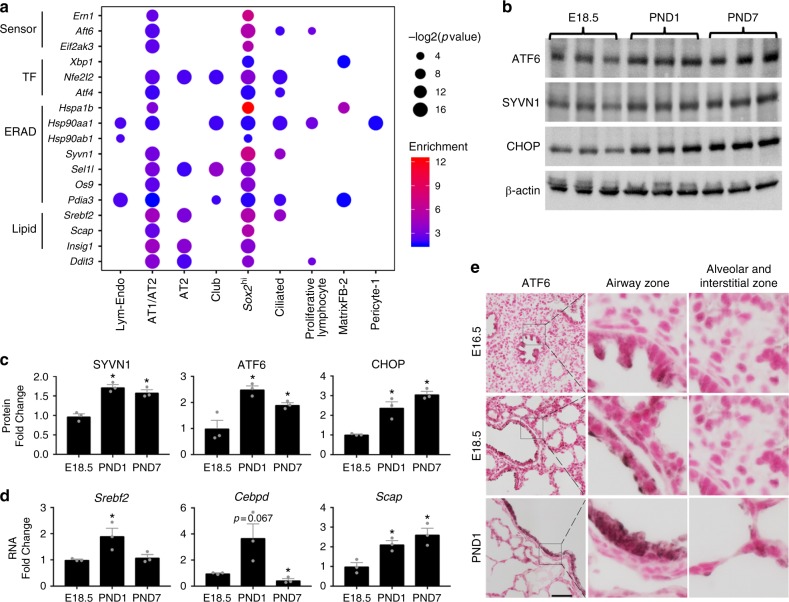
UPR activity in the mouse lung at birth. **a** Associations of unfolded protein response (UPR) gene expression and specific cell types based on the single cell Drop-seq data. Associations (nodes) with Fisher’s exact test *p-*value < 0.05 and enrichment score ≥ 1.5 are shown. Node size is proportional to –log_2_-transformed Fisher’s exact test *p-*value. Node color gradient is proportional to the enrichment score. Sensor stress sensors, TF transcription factors, ERAD endoplasmic reticulum-associated degradation, Lipid lipid biosynthesis. **b** Whole lung lysates from three mice at each age of E18.5, PND1, and PND7 were immunoblotted for ATF6, SYVN1, CHOP, and β-actin. **c** Quantification of immunoblotting after normalization to β-actin. **d** Whole lung RNA was used to quantitate *Scap*, *Cebpd*, and *Srebf2* RNA from three mice at each age of E18.5, PND1, and PND7. **e** ATF6 staining was increased in airway of PND1 compared to E18.5 and E16.5 tissue. Scale bar is 50 μm. Images are representative of at least three embryos/animals for each time point. Boxed regions are zoomed in. In **c** and **d**, *p-value < 0.05 from E18.5 determined by one-way ANOVA with Dunnett’s multiple comparison, mean ± S.E.M. Source data are provided as a Source Data file

In the present study, mRNAs encoding multiple key components in the UPR pathway were induced on PND1, including stress sensors (*Atf6*, *Ern1*, and *Eif2ak3*), key TFs (*Xbp1*, *Atf6*, and *Nrf2*), ERAD components (*Hsp70*, *Hsp90*, *Syvn1*, *Sel1l*, *Os9*, *Ubx*, and *Amer*), and genes involved in lipid biosynthesis (*Srebf2*, *Scap*, *Insig1*, and *Cebpd*) (Supplementary Figure 17), indicating that the transition to postnatal life is associated with ER stress activating UPR in pulmonary cells. The association of key UPR genes with individual cell types was identified from the Drop-seq data. The majority (75%) of cells in which UPR was enriched were epithelial. Among epithelial subtypes, UPR genes were most enriched in AT1/AT2 alveolar cells, and in *Sox2*^hi^ conducting airway epithelial cells (Fig. 6a and Supplementary Data 6).

### Experimental validation of UPR induction at birth

ATF4 and AFT6 are known to play important roles in regulating UPR genes. Bioinformatic analyses suggested that *Atf4* and *Atf6* express in same lung epithelial cell subtypes (Fig. 6a). However, *Atf6* expression was induced at birth while *Atf4* and its transcriptional targets were not induced at birth (Supplementary Figure 17). Consistent with the RNA data, ATF4 and ATF6 immunofluorescence staining was observed in both airway and alveolar cells (Fig. 6e; Supplementary Figure 18; Supplementary Data 6). ATF6 staining was increased in airway cells on PND1 compared with embryonic lung, while ATF4 staining did not change during this period (Fig. 6; Supplementary Figure 18), supporting the concept that ATF6, rather than ATF4, was activated in the stress response after birth.

Other UPR pathway components were assessed by immunoblotting and qPCR. Synoviolin 1 (SYVN1), involved in ER-associated degradation (ERAD) and ubiquitin-dependent degradation of misfolded ER proteins, and DNA damage inducible transcript 3 (DDIT3, also known as CHOP), activated by ER stress and promoting apoptosis, were increased from E18.5 to PND1 and PND7 (Fig. 6b, c). As shown in Supplementary Figure 17, activation of UPR attenuates general protein synthesis and activates lipid biosynthesis via PERK signaling^41,42^. Increased expression of *Srebf2*, *Cebpd*, and *Scap* was identified by quantitative PCR (Fig. 6d), supporting activation of UPR pathway components associated with lipid biosynthesis following birth.

**Fig. 7:**
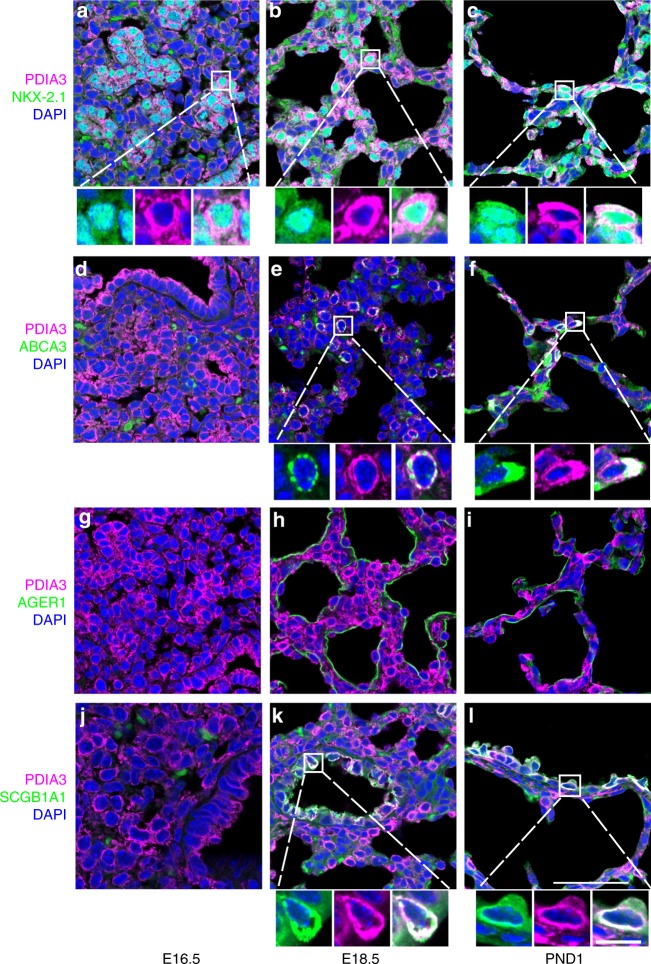
PDIA3 staining in perinatal mouse lung epithelial cells. Mouse lungs were immunostained for PDIA3 and indicated markers of airway and alveolar epithelial cells at E16.5, E18.5, and PND1. PDIA3 co-expressed with NKX2-1 (**a**–**c**), ABCA3 (**d**–**f**), and SCGB1A1 (**j**–**l**). PDIA3 did not co-stain with AGER1 (**g**–**i**). Boxed regions are zoomed in to show individual and merged channels to highlight co-localization of markers at each age. Images are representative of at least three embryos or pups at each age. Scale bar is 50 and 10 μm for the zoomed in box

Protein disulfide isomerase family A member 3 (*Pdia3*) encodes an ER protein, which interacts with calreticulin and calnexin to promote formation of disulfide bonds during protein folding. *Pdia3* RNA was increased at PND1 and was most highly enriched in AT1/AT2, ciliated and lymphatic endothelial cells at PND1 (Fig. 6a). PDIA3 staining was associated with NKX2-1-stained epithelial cells, the latter enriched in AT1/AT2 cells in the single cell RNA study (Fig. 7a–c; Supplementary Data 6). PDIA3 co-localized with the AT2 cell marker ABCA3, the club cell marker SCGB1A1, but not with AT1 cell markers AGER or PDPN (Fig. 7d–l; Supplementary Figure 19). PDIA3 expression was increased during AT2 to AT1 transdifferentiation mediated by WNT signaling^43^, consistent with the present finding that both *Pdia3* and *Axin2* were more enriched in the AT1/AT2 subpopulation.

### Web tool development and data sharing

**Fig. 8:**
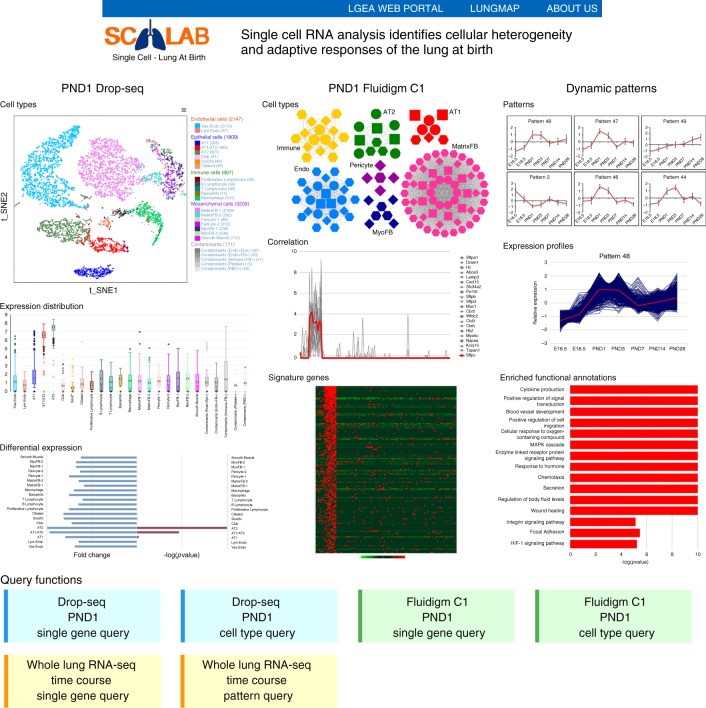
Snapshots of outputs of the scLAB web application. Single cells of Lung At Birth, scLAB (https://research.cchmc.org/pbge/lunggens/SCLAB.html), provides easy accesses and visualizations of the data and results of the present work, including PND1 Drop-seq and Fluidigm C1 single cell RNA-seq data analysis, as well as the dynamic patterns of whole-lung time-course RNA-seq data analysis

To facilitate the query, visualization and re-utilization of the present data, we developed a web application, named “single cells of Lung At Birth” (scLAB), which is freely accessed at https://research.cchmc.org/pbge/lunggens/SCLAB.html. The search tools enables query by gene of interest, cell type, or dynamic gene expression patterns during mouse lung development (Fig. 8). Cell types, cell type-specific signature genes, dynamic gene expression patterns and enriched biological processes are visualized in *t*-distributed stochastic neighbor embedding (tSNE) plots, heatmaps, and profile charts. Data are available in tabular format for downloading and downstream analyses.

## Discussion

We integrated scRNA-seq analysis of PND1 mouse lung with developmental RNA profiles obtained from whole lung tissue to begin to understand the complexity of cellular adaptation of the lung to air breathing at birth. Single cell RNA profiling identifies a diversity of pulmonary cells during perinatal development, and provides access to the genes, processes, and cell–cell interactions regulating pulmonary structure and function at birth. A diversity of cell types, including epithelial, fibroblastic, immune, vascular endothelial, and other rare cell subtypes were identified using selective gene expression patterns. Key bioprocesses and pathways dynamically regulated during the transition to air breathing were identified by integrating single cell and bulk RNA-seq data obtained during the perinatal period. Among the signaling pathways induced at birth, we demonstrated activation of UPR in alveolar epithelial cells, which was accompanied by increased expression of TFs regulating surfactant protein and lipid biosynthesis.

The transition from fetal life to air breathing requires the activation of a series of adaptive responses to the sudden transition from the relatively hypoxic intrauterine environment to the relatively hyperoxic extrauterine environment^44,45^. Altered redox homeostasis in the ER can cause ER stress and induce production of reactive oxygen species in the ER^46^. Increased amount of surfactant proteins, electrolyte and fluid transport proteins synthesized and processed in ER and exposure to ambient oxygen after birth may contribute to the observed increase of UPR and oxidative stress in alveolar and airway epithelial cells. Accumulation of misfolded proteins in ER and changes in redox status may make the newborn vulnerable to additional environmental challenges, which may contribute to the pathogenesis of lung injury causing RDS and BPD in preterm infants^1,6,7^.

A number of important ER resident UPR components were induced at birth, including ATF6, PERK, and IRE1, that function as ER stress sensors activating intracellular signaling pathways to degrade misfolded proteins, regulate protein and lipid biosynthesis and activate molecular chaperones. ATF6, NRF2, and XBP1 encode stress-inducible potent transcriptional activators which regulate target gene expression to enhance protein folding or degradation needed to maintain cell homeostasis under ER and oxidative stress and to promote cell survival^47,48^ (Supplementary Figure 17). Analysis of PND1 single cell data mapped key UPR components to individual cells. Important ER stress sensors and TFs regulating UPR were selectively enriched in alveolar (AT1/AT2) and conducting airway epithelial cells (*Sox2*^hi^) in the newborn lung (Fig. 6a). Consistent with the induction of key components of UPR pathway, expression of genes in a regulatory network controlling surfactant proteins and lipid biosynthesis and metabolism was increased at birth, a time of active synthesis, processing, and secretion of large amounts of surfactant proteins and phosphatidylcholine by AT2 cells. Mutations causing misfolding and mistrafficking of SFTPA/B/C and ABCA3 activate UPR and cause AT2 cell injury and interstitial lung disease in newborn infants and adults^49–51^. SREBP, SCAP, and CEBPD, which play essential roles in the regulation of pulmonary surfactant and phospholipid biosynthesis, were increased at birth^11,52,53^ (Figs. 5 and 6). Pathways regulating lipid synthesis are closely linked to UPR signaling. XBP1, ATF6, and IRE1α directly transactivate SREBP/SCAP and C/EBP to regulate hepatic lipogenesis^54–56^. Taken together, our findings support the hypothesis that the induction of phospholipid biosynthesis and lipid accumulation in lung epithelial cells at birth contributes to ER stress that activates the adaptive phase of UPR to maintain cellular homeostasis.

The regulation of UPR signaling is highly dependent on the nature of the stimulus, as well as the intensity and duration of stress. Early UPR responses attenuate protein synthesis, promoting adaptive responses to restore ER function and to maintain cell survival. Under prolonged ER stress, UPR transitions from an adaptive to an apoptotic response. In the present study, we observed the activation of adaptive responses, i.e., attenuation of protein synthesis, increased lipid biosynthesis, protein refolding and expression of anti-apoptotic protein *Bcl2*. Genes in the apoptotic phase of UPR (e.g., *Casp3/9/12*, *Bak*, and *Bax*) were not induced (Supplementary Figure 17), perhaps indicating that the level and nature of stresses accompanying birth are relatively mild and lipid centric, likely representing pro-survival, rather than cell death responses. However, alveolar epithelial cells under active adaptive stress may be vulnerable to additional external challenges caused by prematurity, infection, cell injury, or mutations in genes causing misfolded proteins. This concept is supported by recent findings, wherein conditional deletion of *Emc3*, an ER membrane protein involved in the ERAD pathway, disrupted both surfactant lipid and protein trafficking, causing activation of UPR in alveolar AT2 cells, resulting in respiratory failure at birth^57^.

While lineage relationships among epithelial cells are increasingly understood in the developing mouse lung, mesenchymal lineage relationships are less well defined. We applied an unbiased and unsupervised iterative clustering approach and identified seven mesenchymal cell subtypes, including two MatrixFB, two pericyte, and three MyoFB/smooth muscle cell subtypes (Fig. 4). The present study provides a resource to gain insight into heterogeneity of lung mesenchymal cells; allowing identification of cell types and potential regulatory mechanisms. Genes involved in WNT (*Wnt2*, *Wnt5a*, and *Axin2*), FGF (*Fgf10*, *Fgf7*, *Fgfr3*, and *Fgfr4*), and T-box family of TFs (*Tbx2*, *Tbx4*, and *Tbx5*) were selectively enriched in the MatrixFB-1 cells (Fig. 4). FGF and WNT signaling often function in a positive regulatory loop^58,59^. Tbx4 and Tbx5 interact during lung growth and branching, and were directly involved in the *Wnt2*/*Fgf10* signaling pathway^60^. Mutations in *TBX4* cause lung hypoplasia and respiratory failure at birth^61^. We hypothesize that T-box family of TFs, WNT and FGF signaling genes interact to form a transcriptional regulatory network influencing differentiation of MatrixFB-1 cells (Supplementary Figure 20). We identified MatrixFB-2 cells expressing high levels of type 1 collagens (*Col1a1 and Col1a2*), ECM components (*Dpt*, *Vcan*, and *Eln*), and *Acta2*. A WNT signaling inhibitor (*Sfrp2)* and insulin-like growth factors and binding proteins (*Igf1*, *Igf2*, and *Igfbp2/5/6*) were highly enriched in MatrixFB-2 cells (Fig. 4a, b). IGF pathway is known to interact with and regulate WNT signaling^62,63^. The present study supports the concept that antagonistic interactions between IGF and WNT/FGF pathways determine the differentiation states and sub-populations of pulmonary mesenchymal cells at birth (Supplementary Figure 20). Recent scRNA-seq analysis of adult mouse lung predicted four distinct subtypes of fibroblasts, including “lipofibroblasts”^64^. While shared similarities with the present findings of the two matrix fibroblasts subtypes in the neonatal mouse lung, difference in signature genes defining the subtypes are evident and no clearly defined “lipofibroblasts” were identified at PND1.

Data and methods integration facilitate comprehensive understanding of adaptive responses in specific pulmonary cells at birth. By integrating Drop-seq single cell data at PND1 with the time course of RNA profiles from whole lung RNA-seq and Fluidigm C1 scRNA-seq, we identified key bioprocesses and pathways that were dynamically regulated during the transition to air breathing. We created pseudo-bulk RNA-seq samples from Drop-seq and Fluidigm C1-based scRNA-seq data from mouse lung at PND1 and compared these with bulk RNA-seq data from different development stages, showing that the pseudo-bulk and bulk RNA-seq samples from PND1 were closely related (Supplementary Figure 21).

While scRNA-seq provides useful insights into the cells and processes active during lung development, there are limitations associated with the present analytic platforms. Low mRNA capture efficiency (~10%) complicates analysis. Important low abundance TFs, signaling molecules and associated bioprocesses and pathways may be lost in scRNA-seq studies. Rare cell types may not be captured from whole lung digests. We observed relatively low numbers of conducting airway cells compared with cells of the peripheral lung (15:1), perhaps consistent with the abundance of peripheral compared to conducting airway tissue. The ratio of pulmonary vascular to lymphatic endothelial cell was 57:1. Whether this reflects actual cell abundance or differences in capture efficiency is unclear. Both lymphatic endothelial and conducting airway cells were detected by Drop-seq but were not detected using Fluidigm C1 RNA-seq analysis and rare pulmonary neuroendocrine cells and nerve cells were not detected by either platform. Single cell analysis combined with tissue microdissection, presorting or laser capture microdissection of specific regions of lung may enhance expression analysis of rare and/or region-specific lung cell populations. For example, recent single cell RNA analyses of pre-sorted airway epithelial cells from conducting airways identified rare lung cell types, including ionocytes, brush cells, and neuroendocrine cells^65,66^.

The present study provides a comprehensive analysis of cells and processes involved in the adaptation to air breathing at birth. Single cell transcriptomic analysis revealed cellular and molecular processes forming and maintaining prenatal lung structure and function. Elucidation of the diversity of pulmonary cells and cell–cell interactions, provides the means to understand the pathogenesis of lung diseases affecting newborn infants. Data from the present study are freely accessed at https://research.cchmc.org/pbge/lunggens/SCLAB.html.

## Methods

### Mice

Animal protocols were approved by the Institutional Animal Care and Use Committee in accordance with NIH guidelines. C57BL6/J mice (Jackson Laboratories), female, PND1 of age, were used in single cell RNA-seq experiments; PDGFRα-GFP^36^ mice (B6.129S4-*PDGFRα*^*tm11****(EGFP)****Sor*^/J, Jackson Laboratories), mixed gender, were used in immunostaining experiments for validating mesenchymal cell subtypes; C57BL6/J mice (Jackson Laboratories), mixed gender, were used in all the other experiments. All mice were time mated. The presence of a vaginal plug was defined as E0.5. PND1 was defined as 24 ± 6 h after birth.

### Immunohistochemistry

Isolated lung tissues were fixed overnight in 4% paraformaldehyde/PBS, equilibrated in 30% sucrose, and embedded in OCT. 7 μm frozen sections were used. Sections from at least three independent animals were evaluated at the indicated developmental time point. Immunohistochemical assays were performed using the methodologies described in detail at https://research.cchmc.org/lungimage. The antibodies and method of antigen retrieval used are indicated in Supplementary Table 3. Confocal images were taken on either a Nikon A1 LUNA inverted, Nikon A1R LUN-V inverted, Nikon A1R inverted, using Nikon elements software. Images were post-processed in either Nikon Elements or IMARIS BITPLANE. Brightfield images were acquired on a Zeiss AXIOIMAGER.A2 using AxioVision software.

### Proximity ligation fluorescent in situ hybridization

Isolated lung tissues were fixed overnight in 4% paraformaldehyde/PBS and embedded in OCT or paraffin. Proximity ligation fluorescent in-situ hybridization (PLISH) was performed as reported in Nagendran et al.^67^ and Gokey et al.^68^, using the hybridization probes for *Sftpc*, *Ager*, and *Scgb1a1* previously reported^15^. In short, slides were pretreated with 10 mM citrate buffer (pH 6.0) for antigen retrieval. Target hybridization probes (From IDT) were incubated at concentrations of 100 nM each in hybridization buffer (1 M sodium trichloroacetate (NaTCA), 5 mM EDTA, 50 mM Tris pH 7.4, 0.2 mg/ml heparin in DEPC water) for 2 h at 37 °C and 100% humidity. Sections were incubated with T4 ligase buffer (NEB cat #M0202) and phosphorylated common bridge and connector circle oligos at 10 nM for 60 min. Subsequent incubations were performed with ligase buffer and T4 ligase for 2 h under the same conditions. DNA amplification was accomplished using Phi-29 polymerase (Lucigen-30221) in polymerase buffer using the same conditions overnight. After the reaction was complete, slides were washed with label buffer (2 × SCC/20% formamide in DEPC water) and incubated with 100 nM fluorescent label tagged label probes in label probe buffer for 1 h using the same conditions. Subsequent immunofluorescent co-staining was performed, and images were obtained by confocal microscopy on a Nikon A1R LUN-V and analyzed on Nikon Elements.

### Immunoblotting

Whole lung lysates were lysed in RIPA buffer containing protease and phosphatase inhibiting cocktails (Thermo Scientific). Protein lysate (25 μg) was loaded into 10–20% Tris–glycine gels (Novex) to separate proteins. An iBlot2 (Invitrogen) was used for dry transfer onto nitrocellulose membranes. Membranes were blocked using 5% bovine serum albumin (BSA) in Tris-buffered saline with 0.1% Tween 20 (TBST) and primary antibodies were diluted in 0.5% BSA/TBST. Western blots were quantified using Image lab software (Biorad). β-actin was used as a loading control. All blots were performed in triplicate (Supplementary Figure 22). Antibodies and concentrations used are listed in Supplementary Table 3. Uncropped blots can be found in Supplementary figure 22.

### qPCR RNA analysis

RNA was isolated from whole lung of three mice each at embryonic and postnatal ages using the RNeasy Micro kit (Qiagen). Reverse transcription was performed using 500 ng RNA and the iScript cDNA synthesis kit (Biorad) to make cDNA. StepOne Plus Real-Time PCR system utilizing TaqMan gene expression assays (Applied Biosystems) was used for qPCR analysis. Each target was ran in triplicate for each sample. RNA assay probes are listed in Supplementary Table 4.

### Cell isolation for flow cytometry

Lungs were isolated and pooled from three newborn mice. Lung tissue was cut into pieces and incubated (37 °C, 30 min) with shaking (150 r.p.m.) in digestion buffer (RPMI 1640 with 10% FBS, 15 mM HEPES, 1% penicillin/streptomycin (wt/vol) and 300 U/ml collagenase VIII) and then pressed through a 100-µm nylon strainer to obtain a single-cell suspension. 1 × 10^7^ cells were washed and then incubated (4 °C, 10 min) with anti-mouse CD16/CD32 and then re-incubated (4 °C, 30 min) with anti-mouse CD45 antibody (30-F11), anti-mouse CD4 antibody (GK1.5), anti-mouse CD8 antibody (53-5.8), anti-mouse CD11b antibody (M1/70), anti-mouse CD11c antibody (N418), anti-mouse CD19 antibody (6D5), anti-mouse Ly6G antibody (1A8), anti-mouse F4/80 antibody (BM8) (all diluted 1:100, Biolegend). Cells were sorted using a LSRII (BD Biosciences) and the data analyzed with FlowJo (Treestar).

### Cell isolation for single cell RNA analyses

Left and right lobes of PND1 mouse lungs were rapidly dissected in ice-cold PBS and finely minced in a Petri dish on ice. Lung pieces were transferred to a 1.5 ml conical tubes using P1 pipetman, cut tip, using 700 μl of TrypLE(10X)/tube and then with 500 μl of collagenase (10 mg/ml in PBS) per tube. The suspension was incubated in a 37 °C water bath for 1 min and triturated for 30 s with cut tip 1 ml blue tip, and repeated until most clumps were gone (7–10 min). Cell suspension was passed through a 40 μm strainer into a 50 ml Falcon tube and the filter rinsed with 5 ml of ice cold PBS with 0.1% BSA, and 15 ml of PBS were added to the filtrate. Cells were centrifuged at 300 × *g* for 5 min at 4 °C. Supernatant was removed. Sigma RBC lysis buffer were added at room temperature. Lysis was monitored microscopically by removing a small aliquot. Cells were washed and pelleted at 300 × *g* for 5 min at 4 °C. Cells were re-suspended in 0.8 ml of PBS with 0.1% BSA, and allowed to settle. The top 3/4 of cells were obtained and the procedure was repeated. Cell nucleus were adjusted to 300 cells per microliter for Fluidigm C1, or 100 cells per microliter for Drop-seq. For Drop-seq experiments, single-cell suspensions were processed through Drop-Seq^13^ to generate single-cell cDNA libraries attached to microbeads. Microbeads were counted, and amplified by PCR, and the 3′ end of the cDNA was prepared for sequencing. Fluidigm C1 experiments were carried out as per Fluidigm recommended protocols.

### Computational analysis of Drop-seq single cell data

The alignment of paired-end sequence reads to mouse genome (mm10) and the generation of digital expression matrix were processed using Drop-seq tools (http://mccarrolllab.com/download/922/, version 1.12)^13,17^. Read2 was aligned with bowtie2-2.2.7 using the “-k 1” option. The aligned reads were tagged with their corresponding UMI and barcode from read1. Each aligned read was tagged with its gene name. The expression matrix was generated by counting the number of unique UMIs per gene per cell.

We removed cells with (1) <500 detected (transcript count > 0) genes, and (2) >10% of transcript counts mapped to mitochondrial genes. We removed genes with transcripts detected in <2 cells. This pre-filtering resulted in using the expression of 19,233 genes in 8003 cells (2388 cells from Batch 1 and 5615 cells from Batch 2) for further analysis. Among the retained 8003 cells, the median number of genes detected per cell was 958 and median number of transcripts per cell was 1790 (Supplementary Figure 1), and 12% of the non-zero values in the filtered expression matrix had a value >3 (Supplementary Figure 1), consistent with previous Drop-seq data^13,17^.

Transcript counts in each cell were normalized by dividing by the total number of transcripts in each cell multiplied by the median number of transcripts per cell. Since data were collected and processed in two batches, we assessed the number of expressed genes and transcripts in cells from different batches and observed a clear batch difference in gene expression related to sample preparation and sequencing (Supplementary Figure 23a). A set of lung-specific negative control genes was used to calculate a scaling factor to reduce the batch difference. Selection of a set of lung-specific negative control genes was described in Supplementary Note 1. We evaluate the batch effect after scaling normalization by assessing expression of commonly used housekeeping genes, including *Actb*, *Actg1*, *B2m*, *Rps6*, *and Rpl13* (all of these genes are expressed in >20% of cells in each batch) and confirmed that our normalization procedure reduced differences between batches (Supplementary Figure 23c). Log_2_(normalized count + 1) was used for further analysis.

Given the normalized expression matrix, the following analytic workflow was employed to map the major cell types and then subtypes within each major cell type. (1) Remove genes that were expressed (transcript count > 0) in <2 cells. (2) Detect highly variable genes using “MeanVarPlot” function in Seurat^69^ for dimension reduction. (3) Perform PCA-based dimension reduction using the *z*-score transformed expression of highly variable genes. PCA was performed using the “prcomp” function in R. (4) Select principal components (PCs) for t-distributed stochastic neighbor embedding (tSNE) analysis and clustering analysis. For major cell-type identification, we selected PCs with standard deviation >3; for subtype identification, we selected PCs with standard deviation >2. (5) Perform tSNE analysis using the scores of the selected PCs using “Rtsne” function in the R package and visual inspection to assess whether there is batch effect on each cell cluster. If there is no such effect for the cluster, analysis proceeds to Step 6. Otherwise, the ComBat method^70^ was used for batch correction on the whole transcriptome of selected cells, and then we repeated steps (2)–(4) using ComBat-corrected data and used new PCs for clustering analysis. ComBat correction was not necessary for analysis of major cell type identification, since we did not observe clear batch effect there (Supplementary Figure 2a); however, ComBat correction was applied in the subtype analysis to remove batch effects (Supplementary Figure 23d, f, h, j). We used tSNE plots to assess successful removal of the batch differences (Supplementary Figure 23e, g, i, k). (6) Use the scores of the selected PCs to discover cell clusters using the graph-based Louvain–Jaccard methods^17^. (7) Identify cluster-specific signature genes. (8) Assign cell clusters to putative cell types based on inspecting the expression of known cell type markers and the identified signature genes. Functional enrichment analysis was applied to cell type signatures using the ToppGene suite^25^ to validate the cell type assignments. Supplementary Figures 2–6 show key results (cell clusters, marker expression, and cell type assignments) in identifying the major cell types, endothelial, epithelial, mesenchymal, and immune sub-populations. We compared cell type assignments from the above integrated analysis and from independent analyses of individual batches (Supplementary Note 2). Results supported the correctness of the batch correction operations and cell type assignments from the integrated analysis. The integrated analysis enabled cell type identification using more cells, improving the resolution of cell type heterogeneity (Supplementary Figure 24).

Cluster/cell type-specific differentially RNA expression was tested using a nonparametric binomial test^17^, which compares the frequency of gene expression in the cluster with its frequency in all the other cells. To define cluster/cell type-specific signature genes, we considered genes that satisfied: (1) <0.05 false discovery rate (FDR) of the binomial test, (2) minimum two-fold effective size, (3) detection in at least 20% of cells in the cluster, and (4) detection in <40% of all cells. The effective size^17^ of a gene in a cluster/cell type is the ratio between the gene’s expression frequency in the cluster/cell type and its expression frequency in all the other cells. For each cluster/cell type, genes were ranked by sensitivity and FDR value, and then up to the top 100 genes which satisfied the above criteria constituted the cluster/cell type specific signature.

### Calculation of sensitivity-based enrichment score

The enrichment of a gene in a specific Drop-seq cell type is calculated as follows: enrichment = (*a*/*b*)/(*c*/*d*), where *a* is the number of cells positively expressing gene *A* in cell type *X*, *b* is the total number of cells positively expressing gene *A*, *c* is the number of cells in cell type *X*, and *d* is the total number of cells. In the calculation, (*a*/*b*) represents the sensitivity of gene expression in the given cell type.

### Driving force analysis for endothelial cell subtypes

For a given cell type, TFs or cofactors differentially expressed (<0.1 FDR of *p*-value of binomial test, ≥2 effective size, ≥20% expression frequency in the cell type, <40% expression frequency in all cells, and ≥20% expression sensitivity) or expressed in more than 60% of the cells in the cell type were selected as candidate TFs. Cell type signature genes were selected as candidate target genes (TGs). Next, the significance of interactions between TF->TF and TF->TG were inferred using the “drivingforce.inferTRN” function in SINCERA^30^. Interactions with significant *p* values were included in reconstructing the cell type-specific TRNs. The largest connected components (LCCs) of the reconstructed TRNs were used for TF importance ranking using the “drivingforce.rankTFs” function in SINCERA. For each cell type, we chose a minimum *p-*value (<0.001) to select significant interactions that preserved about 80% of nodes in the LCC.

### Computational analysis of Fluidigm C1 single-cell RNA-seq

Cells with read count <0.5 million and total number of genes expressed <500 were removed from the analysis. Cells highly expressing selective markers of two distinct major cell types were considered contaminants and were removed from the analysis. After preprocessing, SINCERA pipeline^30^ was used for downstream analysis. Cell clusters were identified using hierarchical clustering with average linkage and Pearson’s correlation-based distance. Cell type assignment was based on cell type-specific marker genes expressions and validated using functional enrichment of cluster-specific differentially expressed genes. Cluster-specific differentially expressed genes were identified using the two-group Welch’s *t*-test-based method in SINCERA^30^.

### Time-course RNA-seq experiment

Embryos and mice for this study were collected from timed pregnant mice. Whole lungs were surgically dissected at embryonic (E) days 16.5, 18.5, and postnatal days (PND) 1, 3, 7, 14, and 28 (Fig. 5). Total RNA was extracted from cells using the QIAamp Circulating Nucleic Acid Kit (QIAGEN). RNA was quality checked, transcribed into complementary DNA using the Verso complementary DNA synthesis kit (AB-1453; LifeTechnologies, Carlsbad, CA), sheared, amplified, adaptor ligated and sequenced for pair-end RNA-Seq reads (Illumina Inc., San Diego, CA, USA). RNA-sequencing was performed by the Cincinnati Children’s Hospital Sequencing Core, with an average read-depth of 30 million reads and average read quality of 37 Phred score for 75 nt pair-end reads. Raw sequencing reads were aligned to mouse genome build GRCm38/mm10 and UCSC reference transcriptome using Tophat 2.0.9, and Partek E/M quantification model. Read counts were further normalized to transcripts per kilobase million (TPM) for downstream analysis. A list of lung-specific negative control genes was used to further normalize gene expression among batches (Supplementary Figure 25; Supplementary Note 1). STEM^40^ was applied to discover significant temporal patterns using the expression profiles of differentially expressed genes. Six significant patterns were identified with *p*-value < 0.05 and cluster size >300 genes. In total, 3716 genes were assigned to these six major expression patterns (Fig. 5).

### Statistics

Statistical analyses were performed using R and GraphPad Prism. One-tailed binomial probability-based test^17^ was used to identify cell type-specific differentially expressed genes in PND1 Drop-seq analysis. One-tailed Welch’s test-based method^30^ was used to identify cell type-specific differentially expressed genes in PND1 Fluidigm C1 data analysis. One-tailed Fisher’s exact test was used to assess the significant gene and cell type association. Two-tailed Chi-square with Yates’ correction was used to assess the significance of *Axin2*^+^ frequency in AT1/AT2 cells. ANOVA with Dunnett’s multiple comparison was used to determine the significant induction of UPR proteins and lipid-related RNAs in mouse lung at PND1.

## Electronic supplementary material

Supplementary Information

Reporting Summary

Description of Additional Supplementary Files

Supplementary Data 1

Supplementary Data 2

Supplementary Data 3

Supplementary Data 4

Supplementary Data 5

Supplementary Data 6

Peer Review File

Source Data

## Data Availability

All data from single-cell and time-course RNA-seq experiments have been deposited in Gene Expression Omnibus under accession code [GSE122332]. Analytic scripts and interpreted results from the present study have been incorporated into LGEA database^26,27^ and scLAB (single cells of lung at birth) web portal, which are freely available at https://research.cchmc.org/pbge/lunggens/SCLAB.html. The authors declare that all data supporting the findings of this study are available within the Article and its Supplementary Information files or from the corresponding authors upon reasonable request.

## References

[1] Hillman NH, Kallapur SG, Jobe AH (2012). Physiology of transition from intrauterine to extrauterine life. Clin. Perinatol..

[2] Graves BW, Haley MM (2013). Newborn transition. J. Midwifery Women’s Health.

[3] Morton SU, Brodsky D (2016). Fetal physiology and the transition to extrauterine life. Clin. Perinatol..

[4] Desai TJ, Brownfield DG, Krasnow MA (2014). Alveolar progenitor and stem cells in lung development, renewal and cancer. Nature.

[5] Whitsett JA, Wert SE, Weaver TE (2010). Alveolar surfactant homeostasis and the pathogenesis of pulmonary disease. Annu. Rev. Med..

[6] Whitsett JA, Matsuzaki Y (2006). Transcriptional regulation of perinatal lung maturation. Pediatr. Clin. North Am..

[7] Gallacher DJ, Hart K, Kotecha S (2016). Common respiratory conditions of the newborn. Breathe.

[8] Kho AT (2010). Transcriptomic analysis of human lung development. Am. J. Respir. Crit. Care Med..

[9] Besnard V (2011). Maternal synchronization of gestational length and lung maturation. PLoS One.

[10] Beauchemin KJ (2016). Temporal dynamics of the developing lung transcriptome in three common inbred strains of laboratory mice reveals multiple stages of postnatal alveolar development. PeerJ.

[11] Xu Y (2012). Transcriptional programs controlling perinatal lung maturation. PLoS One.

[12] Xu Y (2016). Single-cell RNA sequencing identifies diverse roles of epithelial cells in idiopathic pulmonary fibrosis. JCI Insight.

[13] Macosko EZ (2015). Highly parallel genome-wide expression profiling of individual cells using nanoliter droplets. Cell.

[14] Treutlein B (2014). Reconstructing lineage hierarchies of the distal lung epithelium using single-cell RNA-seq. Nature.

[15] Nabhan A, Brownfield DG, Harbury PB, Krasnow MA, Desai TJ (2018). Single-cell Wnt signaling niches maintain stemness of alveolar type 2 cells. Science.

[16] Zepp JA (2017). Distinct mesenchymal lineages and niches promote epithelial self-renewal and myofibrogenesis in the lung. Cell.

[17] Shekhar K (2016). Comprehensive classification of retinal bipolar neurons by single-cell transcriptomics. Cell.

[18] Qiu X (2017). Reversed graph embedding resolves complex single-cell trajectories. Nat. Methods.

[19] Guo M, Bao EL, Wagner M, Whitsett JA, Xu Y (2016). SLICE: determining cell differentiation and lineage based on single cell entropy. Nucleic Acids Res..

[20] Tompkins DH (2009). Sox2 is required for maintenance and differentiation of bronchiolar Clara, ciliated, and goblet cells. PLoS One.

[21] Que J, Luo X, Schwartz RJ, Hogan BL (2009). Multiple roles for Sox2 in the developing and adult mouse trachea. Development.

[22] Tsao PN (2009). Notch signaling controls the balance of ciliated and secretory cell fates in developing airways. Development.

[23] Guseh JS (2009). Notch signaling promotes airway mucous metaplasia and inhibits alveolar development. Development.

[24] Xi Y (2017). Local lung hypoxia determines epithelial fate decisions during alveolar regeneration. Nat. Cell Biol..

[25] Chen J, Bardes EE, Aronow BJ, Jegga AG (2009). ToppGene Suite for gene list enrichment analysis and candidate gene prioritization. Nucleic Acids Res..

[26] Du Y (2017). Lung Gene Expression Analysis (LGEA): an integrative web portal for comprehensive gene expression data analysis in lung development. Thorax.

[27] Du Y, Guo M, Whitsett JA, Xu Y (2015). ‘LungGENS’: a web-based tool for mapping single-cell gene expression in the developing lung. Thorax.

[28] Gordon EJ, Gale NW, Harvey NL (2008). Expression of the hyaluronan receptor LYVE-1 is not restricted to the lymphatic vasculature; LYVE-1 is also expressed on embryonic blood vessels. Dev. Dyn..

[29] Kretschmer S (2013). Visualization of intrapulmonary lymph vessels in healthy and inflamed murine lung using CD90/Thy-1 as a marker. PLoS One.

[30] Guo M, Wang H, Potter SS, Whitsett JA, Xu Y (2015). SINCERA: a pipeline for single-cell RNA-Seq profiling analysis. PLoS Comput. Biol..

[31] Watabe T (2012). Roles of transcriptional network during the formation of lymphatic vessels. J. Biochem..

[32] Hansen A (2010). KSHV-encoded miRNAs target MAF to induce endothelial cell reprogramming. Genes Dev..

[33] Chen L (2010). Tbx1 regulates Vegfr3 and is required for lymphatic vessel development. J. Cell Biol..

[34] De Val S, Black BL (2009). Transcriptional control of endothelial cell development. Dev. Cell.

[35] Takeda N (2004). Endothelial PAS domain protein 1 gene promotes angiogenesis through the transactivation of both vascular endothelial growth factor and its receptor, Flt-1. Circ. Res..

[36] Hamilton TG, Klinghoffer RA, Corrin PD, Soriano P (2003). Evolutionary divergence of platelet-derived growth factor alpha receptor signaling mechanisms. Mol. Cell. Biol..

[37] Guilliams M (2013). Alveolar macrophages develop from fetal monocytes that differentiate into long-lived cells in the first week of life via GM-CSF. J. Exp. Med..

[38] Ziegenhain C (2017). Comparative analysis of single-cell RNA sequencing methods. Mol. Cell.

[39] Svensson V (2017). Power analysis of single-cell RNA-sequencing experiments. Nat. Methods.

[40] Ernst J, Bar-Joseph Z (2006). STEM: a tool for the analysis of short time series gene expression data. BMC Bioinform..

[41] Hetz C (2012). The unfolded protein response: controlling cell fate decisions under ER stress and beyond. Nat. Rev. Mol. Cell Biol..

[42] Xu C, Bailly-Maitre B, Reed JC (2005). Endoplasmic reticulum stress: cell life and death decisions. J. Clin. Invest..

[43] Mutze K, Vierkotten S, Milosevic J, Eickelberg O, Konigshoff M (2015). Enolase 1 (ENO1) and protein disulfide-isomerase associated 3 (PDIA3) regulate Wnt/beta-catenin-driven trans-differentiation of murine alveolar epithelial cells. Dis. Models Mech..

[44] Muller DP (1987). Free radical problems of the newborn. Proc. Nutr. Soc..

[45] Burri PH (1984). Fetal and postnatal development of the lung. Annu. Rev. Physiol..

[46] Cao SS, Kaufman RJ (2014). Endoplasmic reticulum stress and oxidative stress in cell fate decision and human disease. Antioxid. Redox Signal..

[47] Yoshida H, Matsui T, Yamamoto A, Okada T, Mori K (2001). XBP1 mRNA is induced by ATF6 and spliced by IRE1 in response to ER stress to produce a highly active transcription factor. Cell.

[48] Chen CY (2014). Signal peptide peptidase functions in ERAD to cleave the unfolded protein response regulator XBP1u. EMBO J..

[49] Whitsett JA, Wert SE, Weaver TE (2015). Diseases of pulmonary surfactant homeostasis. Annu. Rev. Pathol..

[50] Wert SE, Whitsett JA, Nogee LM (2009). Genetic disorders of surfactant dysfunction. Pediatr. Dev. Pathol..

[51] Rindler, T. N. et al. Alveolar injury and regeneration following deletion of ABCA3. *JCI Insight* **2**, e97381 (2017). 10.1172/jci.insight.97381 PMC5752264 29263307

[52] Bridges JP (2014). Epithelial SCAP/INSIG/SREBP signaling regulates multiple biological processes during perinatal lung maturation. PLoS One.

[53] Martis PC (2006). C/EBPalpha is required for lung maturation at birth. Development.

[54] Zhang K (2011). The unfolded protein response transducer IRE1alpha prevents ER stress-induced hepatic steatosis. EMBO J..

[55] Fu S (2011). Aberrant lipid metabolism disrupts calcium homeostasis causing liver endoplasmic reticulum stress in obesity. Nature.

[56] Yamamoto K (2010). Induction of liver steatosis and lipid droplet formation in ATF6alpha-knockout mice burdened with pharmacological endoplasmic reticulum stress. Mol. Biol. Cell.

[57] Tang, X. et al. EMC3 coordinates surfactant protein and lipid homeostasis required for respiration. *J. Clin. Invest*. **127**, 4314–4325 (2017). 10.1172/JCI94152 PMC5707157 29083321

[58] Stulberg MJ, Lin A, Zhao H, Holley SA (2012). Crosstalk between Fgf and Wnt signaling in the zebrafish tailbud. Dev. Biol..

[59] Volckaert T, De Langhe SP (2015). Wnt and FGF mediated epithelial-mesenchymal crosstalk during lung development. Dev. Dyn..

[60] Arora R, Metzger RJ, Papaioannou VE (2012). Multiple roles and interactions of Tbx4 and Tbx5 in development of the respiratory system. PLoS Genet..

[61] Suhrie, K. et al. Neonatal lung disease associated with TBX4 mutations. *J. Pediatr.*10.1016/j.jpeds.2018.10.018 (2018). 10.1016/j.jpeds.2018.10.018 PMC6389379 30413314

[62] Richard-Parpaillon L, Heligon C, Chesnel F, Boujard D, Philpott A (2002). The IGF pathway regulates head formation by inhibiting Wnt signaling in Xenopus. Dev. Biol..

[63] Carron C, Bourdelas A, Li HY, Boucaut JC, Shi DL (2005). Antagonistic interaction between IGF and Wnt/JNK signaling in convergent extension in Xenopus embryo. Mech. Dev..

[64] Xie T (2018). Single-cell deconvolution of fibroblast heterogeneity in mouse pulmonary fibrosis. Cell Rep..

[65] Montoro DT (2018). A revised airway epithelial hierarchy includes CFTR-expressing ionocytes. Nature.

[66] Plasschaert LW (2018). A single-cell atlas of the airway epithelium reveals the CFTR-rich pulmonary ionocyte. Nature.

[67] Nagendran, M., Riordan, D. P., Harbury, P. B. & Desai, T. J. Automated cell-type classification in intact tissues by single-cell molecular profiling. *eLife* **7**, e30510 (2018). 10.7554/eLife.30510 PMC5802843 29319504

[68] Gokey, J. J. et al. Active epithelial Hippo signaling in idiopathic pulmonary fibrosis. *JCI Insight* **3**, e98738 (2018). 10.1172/jci.insight.98738 PMC5926907 29563341

[69] Satija R, Farrell JA, Gennert D, Schier AF, Regev A (2015). Spatial reconstruction of single-cell gene expression data. Nat. Biotechnol..

[70] Johnson WE, Li C, Rabinovic A (2007). Adjusting batch effects in microarray expression data using empirical Bayes methods. Biostatistics.

