# Supplementary material for: Single cell RNA analysis identifies cellular heterogeneity and adaptive responses of the lung at birth
Source: Nat Commun. 2019 Jan 3;10:37. doi: 10.1038/s41467-018-07770-1 (PMC6318311; doi:10.1038/s41467-018-07770-1)
Supplement: Supplementary file 1 — Supplementary Information [file 41467_2018_7770_MOESM1_ESM.pdf]

Supplementary Information

Single cell RNA analysis identifies cellular heterogeneity and adaptive responses of the lung at birth

Guo et al.

Supplementary Tables

Supplementary Table 1. Number of batches and cells in Drop-seq and Fluidigm C1 single-cell RNA-seq of mouse lung on postnatal day 1 (PND1).

Platform	Batch	Number of cells	Cells after QC
Drop-seq	Batch1	2,389	2,388
	Batch2	7,555	5,615
Fluidigm C1	Batch1	39	27
	Batch2	19	17
	Batch3	47	33
	Batch4	63	53

Supplementary Table 2. Batches and number of samples in time-course bulk RNA-seq of developing mouse lung.

Developmental time	Batch	Number of samples
E16.5	Batch1	3
E18.5	Batch1	3
PND1	Batch3	2
PND3	Batch3	2
PND7	Batch1	2
PND14	Batch2	2
PND28	Batch1	2

Supplementary Table 3. List of antibodies used for immunohistochemistry and immunoblotting analysis.

Antigen	Source	Host species	Clone/Lot	Antigen Retrieval with heat	Concentration used for IHC or IF	Dilution used for Western or FACS
ABCA3	CCHMC in house (GP985)	Guinea pig		Citrate[1]	1:200 (IF)	
ACTA2	Sigma A5228	Mouse IgG2a	Clone 1A4 lot 065M4762V	Not required/Citrate	1:1500	
AGER	R&D Systems (MAB1179-500)	Rat	JYK0214091 lot H	Citrate	2.5 ug/ml (IF)	
ATF4	Abcam (ab184909)	Rabbit	GR321752-7	Citrate	1:2000 (IHC) 1:200 (IF)	
ATF6	Novus (NBP1-40256)	Mouse IgG1	Clone 70B1413.1			1:2000
ATF6	ThermoFisher (PA520215)	Rabbit	TB2528944	Citrate	1:1000 (IHC) 1:200 (IF)	1:1000
CHOP	Cell Signaling (2895T)	Mouse IgG2a	Clone L63F7 lot 11			1:1500
CSPG4	Millipore (AB5320)	Rabbit	2726769	Citrate	1:100	
EMCN	R&D Systems (AF4666)	Goat	CAAS021702	Citrate	1 ug/ml (IF)	
FOXJ1*	eBiosciences (14-9965-82)	Mouse IgG1	Clone 2A5	Citrate	1:500 (IF)	
FN1	Abcam (ab2413)	Rabbit	GR3210190-1	Citrate	1:100 (IF)	
GFP	Abcam (ab13970)	Chicken	R236651-21	Citrate	1:100 (IF)	
IGFBP5	Santa Cruz Biotechnology (sc-515116)	Mouse IgA	L1317	Citrate	1:75 (IF)	
LYVE1	Abcam (ab14917)	Rabbit	GR274834-2	Citrate	1:100	
NKX2.1	CCHMC in house (GP237)	Guinea pig		Citrate	1:100 (IF)	
PDGFRβ	R&D Systems (AF1042)	Goat	60V0418031	Citrate	7.9 ug/ml (IF)	
PDIA3	ThermoFisher (PA3-009)	Rabbit	SC250765	Citrate	1:200 (IF)	
PDPN	DSHB (8.1.1-c)	Syrian Hamster	42414-145ug/ml1g	Citrate	1:100	
SCGB1A1	CCHMC in house (GP210)	Guinea pig		Citrate	1:1500 (IF)	
SFRP2	Santa Cruz	Rabbit	J1512	Citrate	1:100 (IF)	

	Biotechnology (SC-13940)					
SOX2	Seven Hills Bioreagents (WRAB-1236)	Rabbit		Citrate	1:2000 (IF)	
SOX17	CCHMC in house (GP	Guinea pig		Citrate	1:400 (IF)	
SYVN1	Cell Signaling (14773S)	Rabbit	1			1:1500
TGFBI	Abcam (ab170874)	Rabbit	GR126611-25	Citrate	1:100 (IF)	
CD45	Biolegend (10311)	Rat IgG2b	30-F11			1:100
CD4	Biolegend (100426)	Rat IgG2b	GK1.5			1:100
CD8b	Biolegend (140415)	Rat IgG1	53-5.8			1:100
CD11b	Biolegend (101219)	Rat IgG2b	M1/70			1:100
CD11c	Biolegend (117337)	Armenian Hamster	N418			1:100
CD19	Biolegend (152405)	Rat IgG2a	1D3			1:100
LYG6	Biolegend (127643)	Rat IgG2a	1A8			1:100
F4/80	Biolegend (123149)	Rat IgG2a	BM8			

*The signal using this antibody was amplified using a biotinylated secondary and a strept-avidin conjugated fluorophore.

[1]Citrate buffer pH 6.0 with heat

Supplementary Table 4. List of Taqman assay probes from Applied Biosystems (Thermo Fisher) used to assess the lipid associated unfolded protein response pathway.

**Target**	**Taqman assay #**
Srebf2	Mm00843434_s1
Cebpd	Mm01306292_m1
Scap	Mm01250176_m1

Supplementary Table 5. Candidate markers defining each putative Drop-seq cell type in mouse lung at PND1.

**Putative Drop-seq cell type**	**Candidate Markers**
Vas-Endo	*Sox17, Emcn, Plvap, Clec1a*
Lym-Endo	*Prox1, Thy1, Ccl21b, Ccl21a*
AT1	*Pdpn, Aqp5, Ager, Hopx*
AT1/AT2	*Muc1*$^{+}$*/Pdpn*$^{+}$*, Egfr, Shh*
AT2	*Sftpb, Slc34a2, Abca3, Lpcat1*
Club	*Scgb1a1, Scgb3a2, Cyp2f2, Krt15*
Ciliated	*Foxj1, Ccdc113, Lrrc6*, *1700028P14Rik*
Sox2^{hi}	*Sox2, Foxj1*$^{+}$*/Krt15*$^{+}$*, Katnal2, Hs3st6, Spag16*
Proliferative Lymphocyte	*Mki67, Top2a, Hells, Ighm, Hist1h2bj, Hist1h4i, Hist1h4c, Hist1h2bq, Hist1h2ak, Hist1h2br*
B Lymphocyte	*Ighm, Igkc, Cd19, Cd79a, Cd79b, Ms4a1*
T Lymphocyte	*Cd3g, Cd3d, Cd7, Trbc1, Trbc2*
Basophils	*Fcer1a, Cd200r3, Mcpt8, Cyp11a1*
Macrophage	*Spi1, Tyrobp, Fcer1g, Fcgr3, Mpeg1, Fcgr2b, Emr1*
MatrixFB-1	*Tcf21, Wnt2, Fn1, Fgf10, Inmt*
MatrixFB-2	*Col1a1, Col1a2, Agtr2, Mfap5, Sfrp2*$^{+}$*/Igfbp5*$^{+}$
Pericyte-1	*Pdgfrb, Notch3, Map3k7cl, Mustn1, Acta2*
Pericyte-2	*Pdgfrb, Notch3, Agtr1a, Vsnl1, Art3*
MyoFB-1	*Tgfbi*, *Pdgfra*$^{+}$*/Acta2*$^{-}$
MyoFB-2	*Tgfbi*, *Pdgfra*$^{+}$*/Acta2*$^{+}$
Smooth Muscle	*Tgfbi*, *Pdgfra*$^{-}$*/Acta2*$^{+}$*, Actg2, Cnn1, Des*

Supplementary Figures

	Shekhar et al.,2016 (27,499 cells)	Batch1 of PND1 (2,388 cells)	Batch2 of PND1 (5,615 cells)	PND1 (8,003 cells)
**Number of Detected Genes per Cell**				
Min.	454	497	424	424
1st Qu.	644	639	777	717
Median	810	785	1047	958
Mean	878.4	919	1285	1176
3rd Qu.	1033	1037	1469	1342
Max.	6182	6261	10050	10050
**Number of Transcript Counts per Cell**				
Min.	551	657	615	615
1st Qu.	911	1040	1335	1208
Median	1192	1384	2012	1790
Mean	1344	1853	3244	2829
3rd Qu.	1602	2077	3401	2974
Max.	28740	27260	114700	114700
**Distribution of Non-zero Transcript Counts**				
**Expression with 1 UMI**	**75%**	**67%**	**63%**	**64%**
**Expression with 2 UMIs**	**16%**	**17%**	**17%**	**17%**
**Expression with 3 UMIs**	**5%**	**6%**	**7%**	**7%**
**Expression with >3 UMIs**	**4%**	**10%**	**12%**	**12%**

Supplementary Figure 1. The quality metrics of Drop-seq of mouse lung at PND1 after pre-filtering and Drop-seq of mouse bipolar retina[1]. Genes with at least 1 transcript count were considered as detected genes.

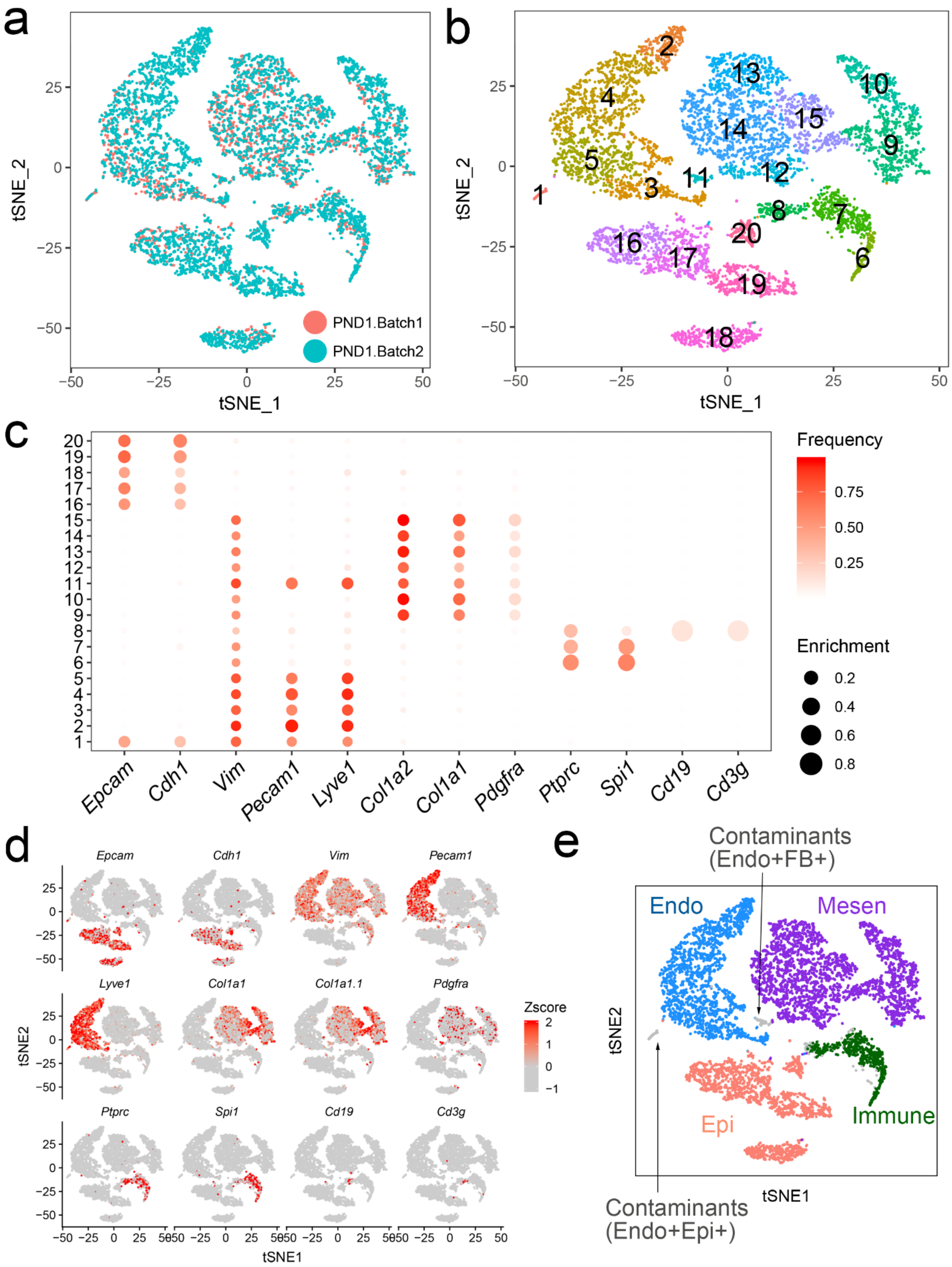
a
b
c
d
e
tSNE_1
tSNE_2
PND1.Batch1
PND1.Batch2
Frequency
Enrichment
Epcam
Cdh1
Vim
Pecam1
Lyve1
Col1a2
Col1a1
Pdgfra
Ptprc
Spi1
Cd19
Cd3g
Col1a1.1
Zscore
tSNE1
tSNE2
Contaminants (Endo+FB+)
Endo
Mesen
Epi
Immune
Contaminants (Endo+Epi+)

Supplementary Figure 2. Identification of lung major cell types using Drop-seq of mouse lung at PND1. (**a**) t-distributed Stochastic Neighbor Embedding (tSNE) analysis of cells. Cells were colored by batch information. (**b**) Visualization of cell clusters in tSNE plot of cells. Cell clusters were unbiasedly identified using the Louvain-Jaccard algorithm[1]. (**c**) Expression frequency and sensitivity based enrichment scores of known cell type markers in individual cell clusters. Enrichment scores were per gene max normalized for visualization. (**d**) Expression of known cell type markers in tSNE plot of cells. (**e**) The assignment of cell clusters to four major cell types and two potentially contaminated populations, including endothelial cells (Endo, blue), mesenchymal cells (Mesen, purple), immune cells (Immune, cells in green color), epithelial cells (Epi, red), contamination of endothelial and epithelial cells (Endo+Epi+, grey), and contamination of endothelial and fibroblast cells (Endo+FB+, grey).

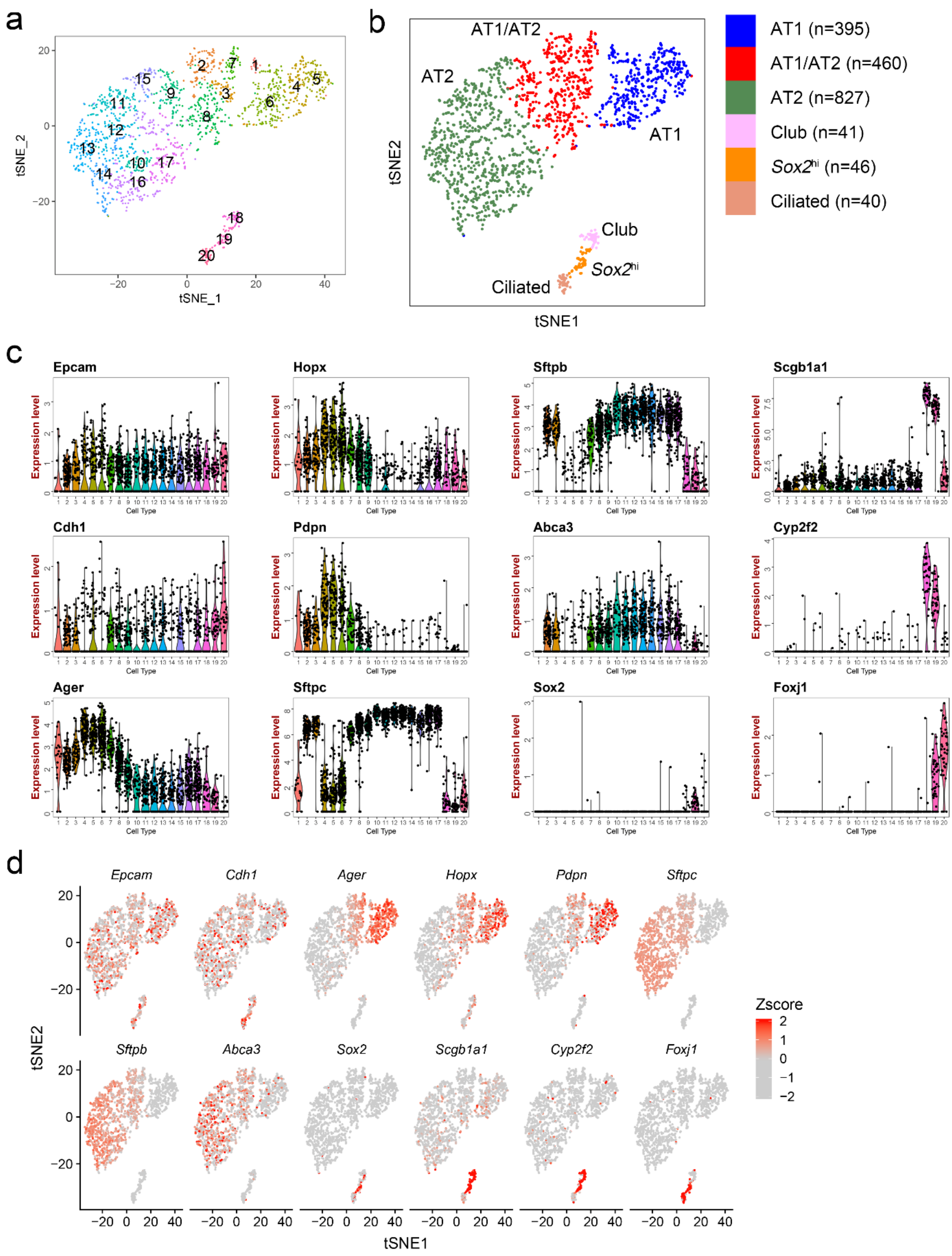

a
b
AT1/AT2
AT2
AT1
Club
Sox2hi
Ciliated
tSNE1
tSNE2
tSNE_1
tSNE_2
AT1 (n=395)
AT1/AT2 (n=460)
AT2 (n=827)
Club (n=41)
Sox2hi (n=46)
Ciliated (n=40)
c
Epcam
Hopx
Sftpb
Scgb1a1
Cdh1
Pdpn
Abca3
Cyp2f2
Ager
Sftpc
Sox2
Foxj1
Expression level
Cell Type
d
Zscore

Supplementary Figure 3. Identification of lung epithelial subtypes using Drop-seq of mouse lung at PND1. (**a**) Visualization of cell clusters in the t-distributed Stochastic Neighbor Embedding (tSNE) plot. Epithelial cell clusters were unbiasedly identified using the Louvain-Jaccard algorithm[1]. (**b**) 20 cell clusters were assigned to 6 epithelial subtypes based on inspecting the expression of known marker expression and functional annotations enriched by predicted signature genes. (**c**) Violin plots of the expression of known lung epithelial (*Epcam*, *Cdh1*), distal lung epithelial (*Ager*, *Hopx*, *Pdpn*, *Sftpc*, *Sftpb*, *Abca3*), and lung airway epithelial (*Sox2*, *Scgb1a1*, *Cyp2f2*, *Foxj1*) markers in individual cell clusters. (**d**) The expression of known lung epithelial markers in tSNE plot of epithelial cells.

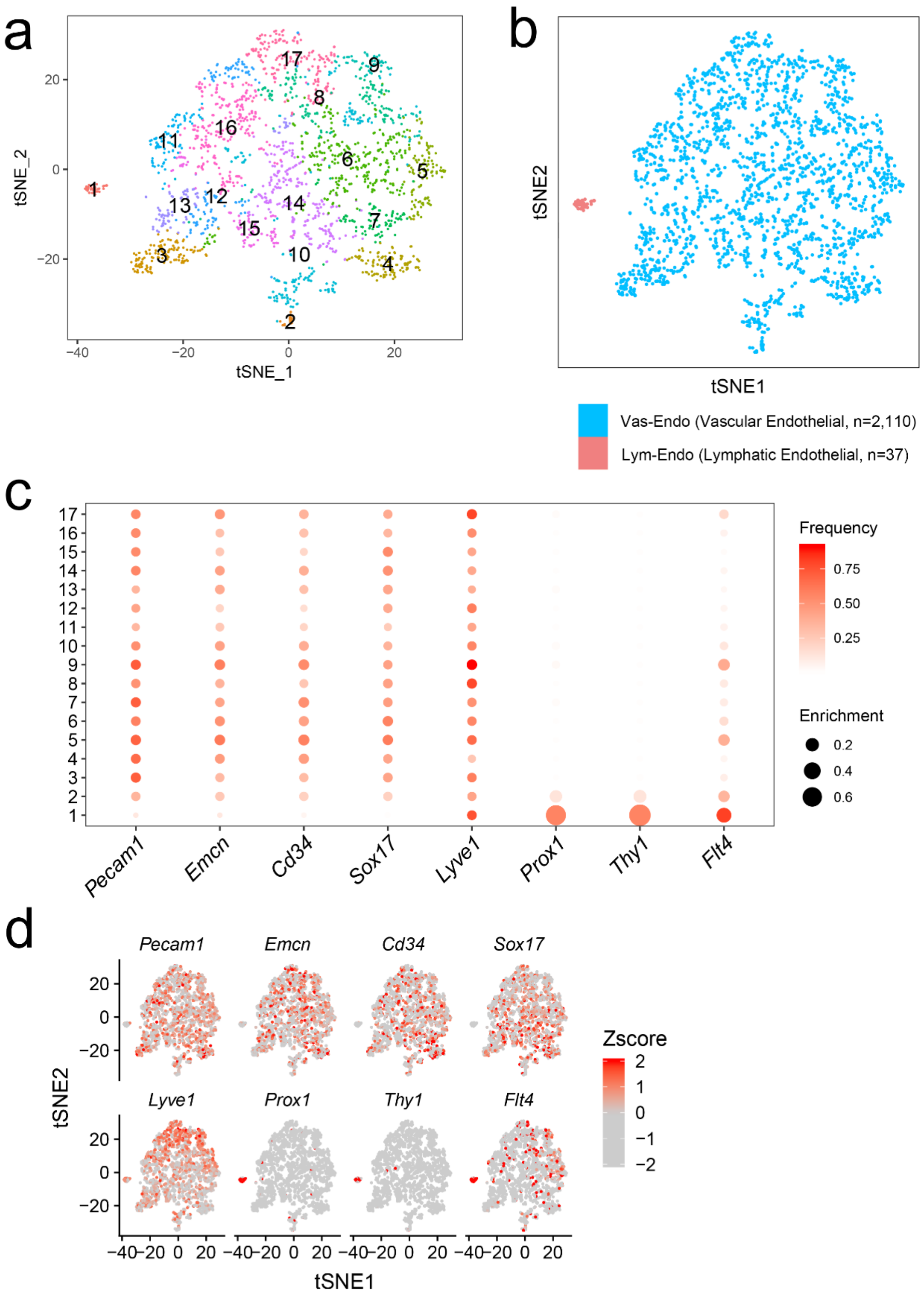

Supplementary Figure 4. Identification of lung endothelial subtypes using Drop-seq of mouse lung at PND1. (**a**) Visualization of cell clusters in the t-distributed Stochastic Neighbor Embedding (tSNE) plot. Endothelial cell clusters were unbiasedly identified using the Louvain-Jaccard algorithm[1]. (**b**) 17 cell clusters were assigned to two endothelial subtypes based on

inspecting the expression of known marker expression and functional annotations enriched by predicted signature genes. (**c**) Expression frequency and sensitivity based enrichment scores of known lymphatic endothelial (*Lyve1*, *Thy1*, *Prox1*, *Flt4*, *Pecam1*) and vascular endothelial (*Emcn*, *Sox17*, *Cd34*, *Pecam1*) markers in individual cell clusters. Enrichment scores were per gene max normalized for visualization. (**d**) The expression of known lymphatic and vascular endothelial markers in tSNE plot of endothelial cells.

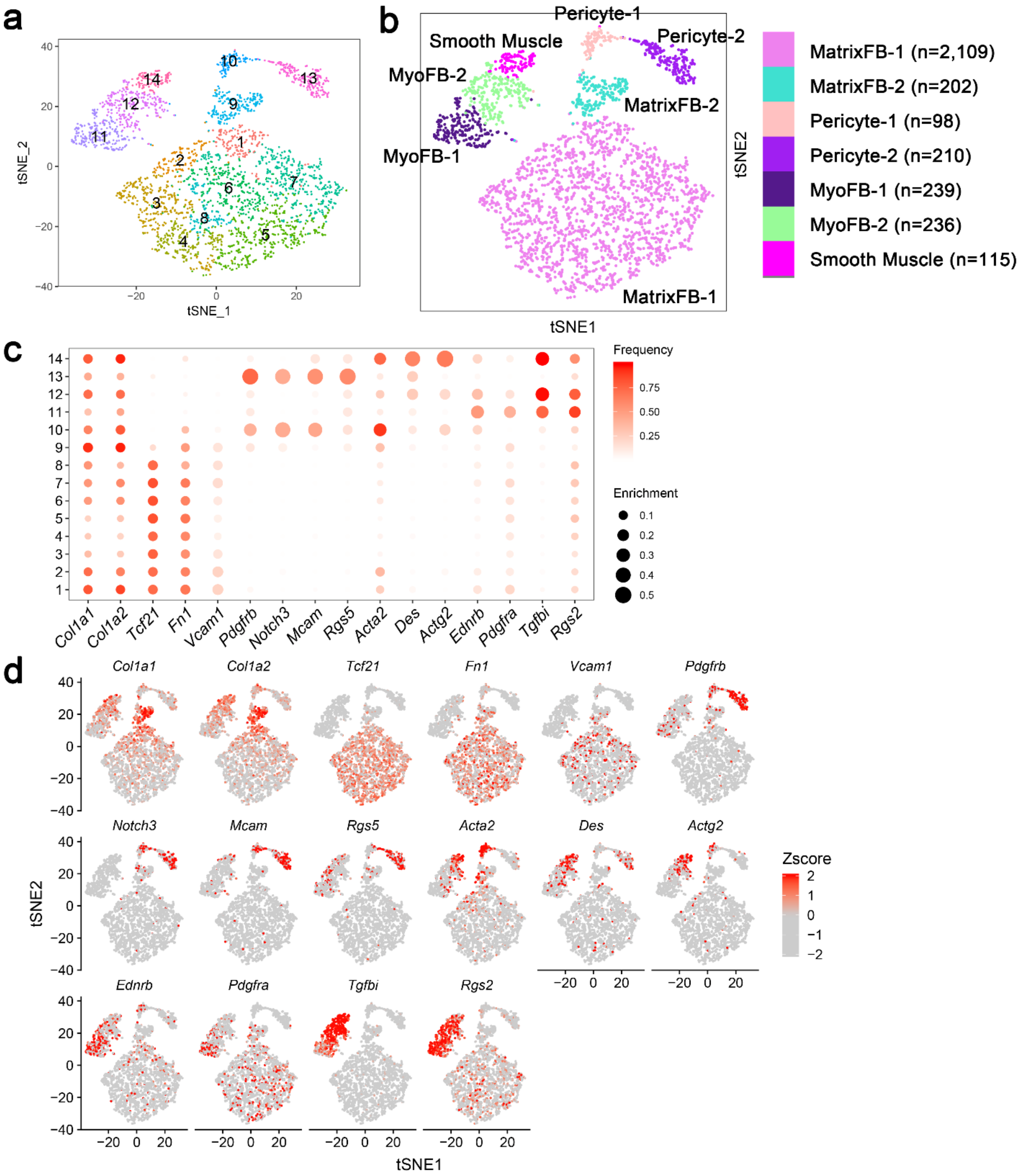

Supplementary Figure 5. Identification of lung mesenchymal subtypes using Drop-seq of mouse lung at PND1. (**a**) Visualization of cell clusters in the t-distributed Stochastic Neighbor Embedding (tSNE) plot. Mesenchymal cell clusters were unbiasedly identified using the Louvain-Jaccard algorithm[1]. (**b**) 14 cell clusters were assigned to 7 mesenchymal subtypes based on inspecting the expression of known marker expression and functional annotations enriched by predicted signature genes. (**c**) The expression frequency and sensitivity based enrichment scores of lung fibroblast (*Col1a1, Col1a2, Tcf21*, Fn1, Vcam1),

myofibroblast/smooth muscle (*Pdgfra*, *Ednrb*, *Acta2*, *Actg2*, *Des*, *Tgfbi*, *Rgs2*), and pericyte (*Pdgfrb, Notch3, Mcam, Rgs5*) markers and signature genes in individual cell clusters. Enrichment scores were per gene max normalized. (**d**) The expression of lung fibroblast, myofibroblast, smooth muscle, and pericyte markers and signature genes in tSNE plot of mesenchymal cells.

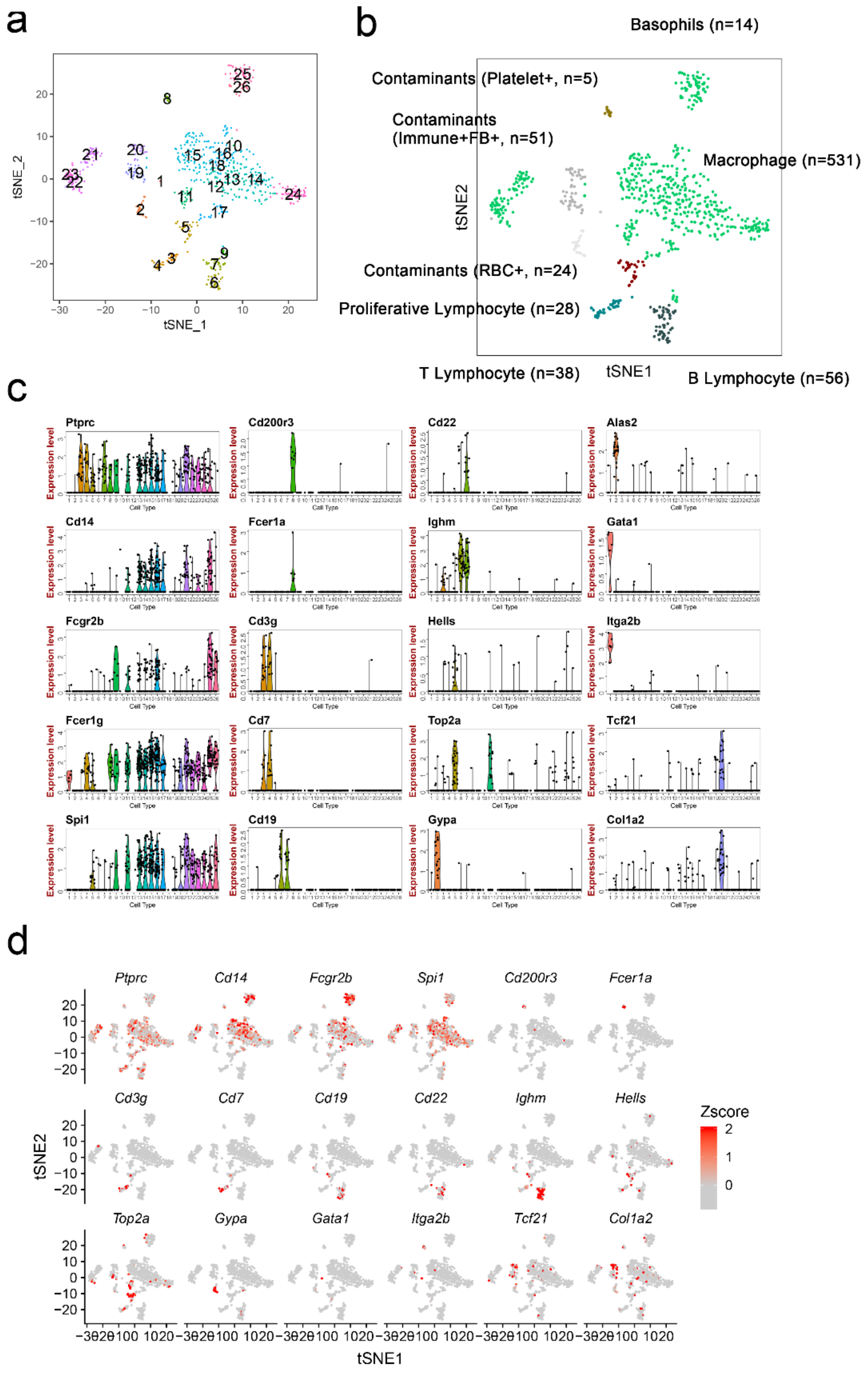

Supplementary Figure 6. Identification of lung immune subtypes using Drop-seq of mouse lung at PND1. (**a**) Visualization of cell clusters in the t-distributed Stochastic Neighbor Embedding (tSNE) plot. Immune cell clusters were unbiasedly identified using the Louvain-Jaccard algorithm[1]. (**b**) 26 cell clusters were assigned to five immune subtypes and three

potential contaminated cell populations based on inspecting the expression of known marker expression. (**c**) Violin plots of the expression of leukocyte pan marker (*Ptprc*), monocyte/macrophage (*Cd14*, *Fcgr2b*, *Spi1*), Basophils (*Cd200r3*, *Fcer1a*), lymphocyte (*Cd3g*, *Cd7*, *Cd19*, *Cd22*, *Hells*, *Ighm*), megakaryocyte (*Gata1*, *Itga2b*), erythrocyte (*Gypa*), and fibroblast cell (*Tcf21, Col1a2*) markers in individual cell clusters. (**d**) The expression of cell type markers or signature genes in tSNE plot of immune cells.

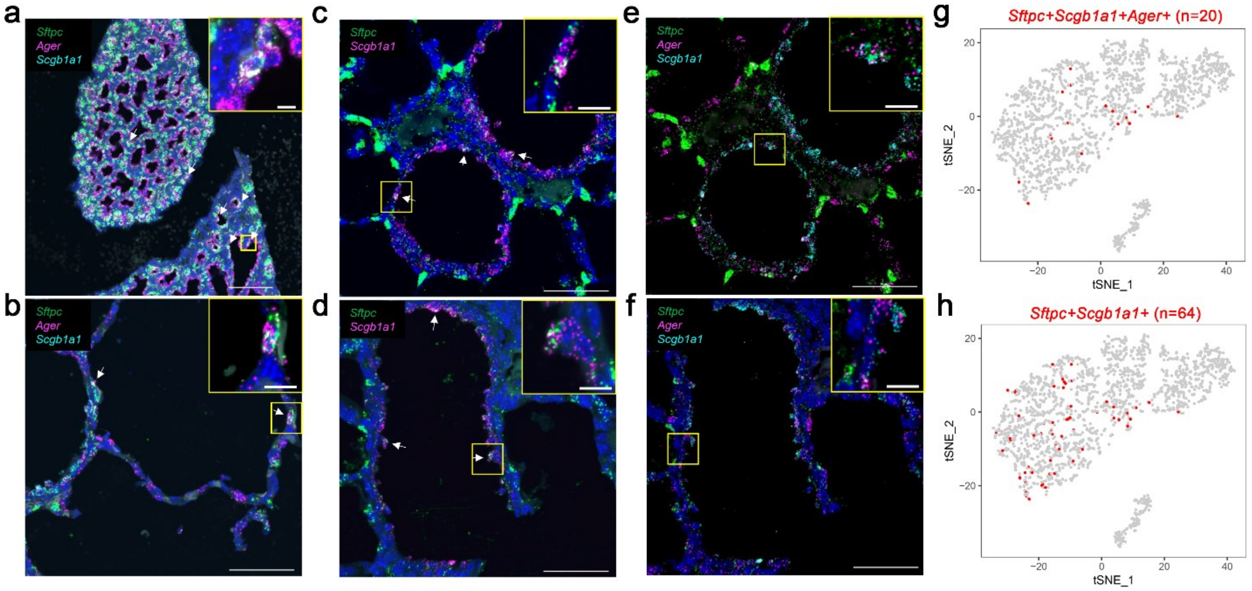

Supplementary Figure 7. Epithelial cells co-expressing *Sftpc*, *Ager*, and/or *Scgb1a1* in mouse lung. (**a-b**) Proximity ligation fluorescent in-situ hybridization (PLISH) for *Sftpc*, *Ager*, and *Scgb1a1* RNAs in peripheral regions of (**a)** E18.5 mouse lungs, scale bar is 100mm and inset is 5mm, and **(b)** PND1 mouse lungs, scale bar is 50mm and inset is 5mm. White arrows point to cells co-expressing *Sftpc* and *Ager*. (**c-d**) PLISH for cells co-expressing *Sftpc* and *Scgb1a1* RNAs in PND1 mouse lungs. Scale bar is 50mm and inset is 5mm. (**e-f**) PLISH for cells co-expressing *Sftpc*, *Ager*, and *Scgb1a1* RNAs in PND1 mouse lungs. Six *Sftpc*+/*Ager*+/*Scgb1a1*+ cells were detected, representing 0.1% of total DAPI positive cells. Scale bar is 50mm and inset is 5mm. (**g**) Epithelial cells (red, n=20) highly expressing *Sftpc*, *Ager*, and *Scgb1a1* in the Drop-seq of mouse lung at PND1. (**h**) Epithelial cells (red, n=64) highly expressing *Sftpc* and *Scgb1a1* in the Drop-seq of mouse lung at PND1. Zscore>=1 was used as the criterion for selective expression. In **a-f**, images are representative of 3 mice. In **g-h**, cells are from Drop-seq of two individual mice.

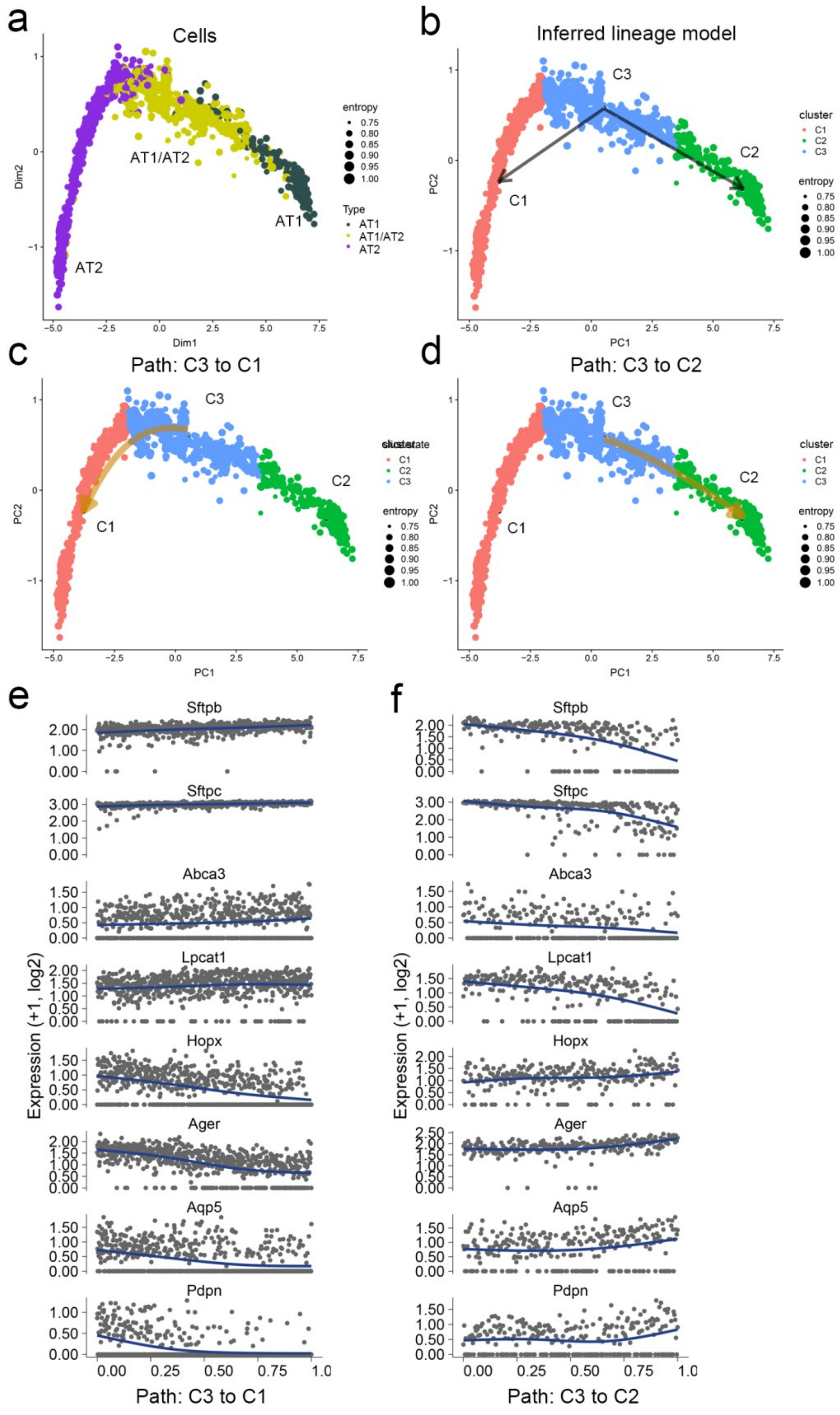

Supplementary Figure 8. Predicted differentiation lineage model among the three peripheral epithelial subpopulations in mouse lung at PND1. (**a**) Cells of the three distal epithelial subpopulations (AT1, AT1/AT2, AT2) in a reduced dimensional space calculated by DDRTree method in “Monocle 2”[2]. (**b**) SLICE[3] measured the entropy of individual cells, identified three cell states/clusters, C1, C2, and C3, among the cells, and inferred a branched

differentiation lineage model. (**c**) SLICE predicted a principal curve based entropy-directed differentiation trajectory from cluster C3 to cluster C1. (**d**) SLICE predicted a principal curve based entropy-directed differentiation trajectory from cluster C3 to cluster C2. (**e**) Expression patterns of AT1 and AT2 markers following the trajectory from C3 to C1. (**f**) Expression patterns of AT1 and AT2 markers following the trajectory from C3 to C2.

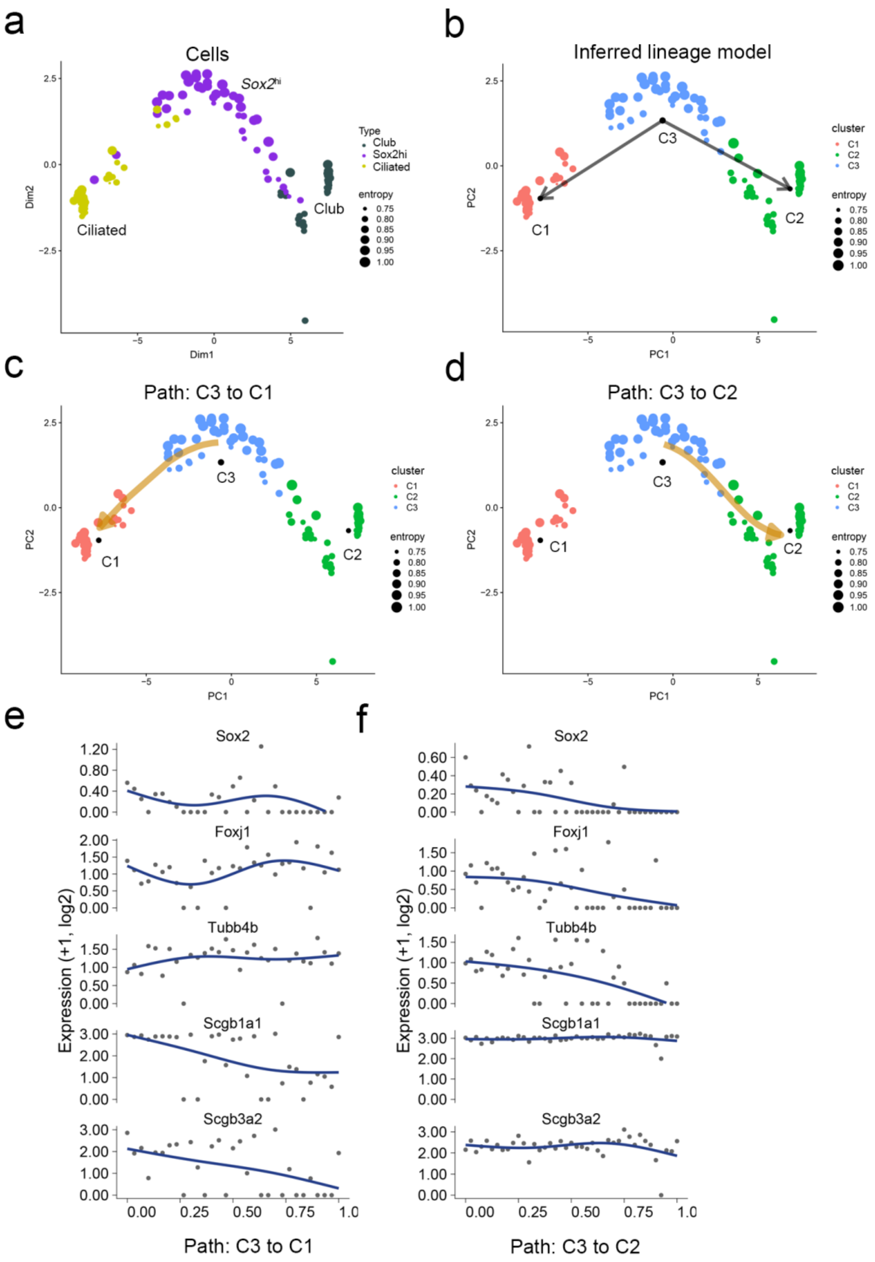

Supplementary Figure 9. Predicted differentiation lineage model among the three proximal airway epithelial subpopulations in mouse lung at PND1. (**a**) Cells of the three airway epithelial subpopulations (Club, *Sox2*^{hi}, Ciliated) in a reduced dimensional space calculated by DDRTree method in "Monocle 2"[2]. (**b**) SLICE[3] measured the entropy of individual cells, identified three cell states/clusters, C1, C2, and C3, among the cells, and inferred a branched differentiation lineage model. (**c**) SLICE predicted a principal curve based entropy-directed differentiation trajectory from cluster C3 to cluster C1. (**d**) SLICE predicted a principal curve based entropy-directed differentiation trajectory from cluster C3 to cluster C2. (**e**) Expression patterns of AT1 and AT2 markers following the trajectory from C3 to C1. (**f**) Expression patterns of AT1 and AT2 markers following the trajectory from C3 to C2.

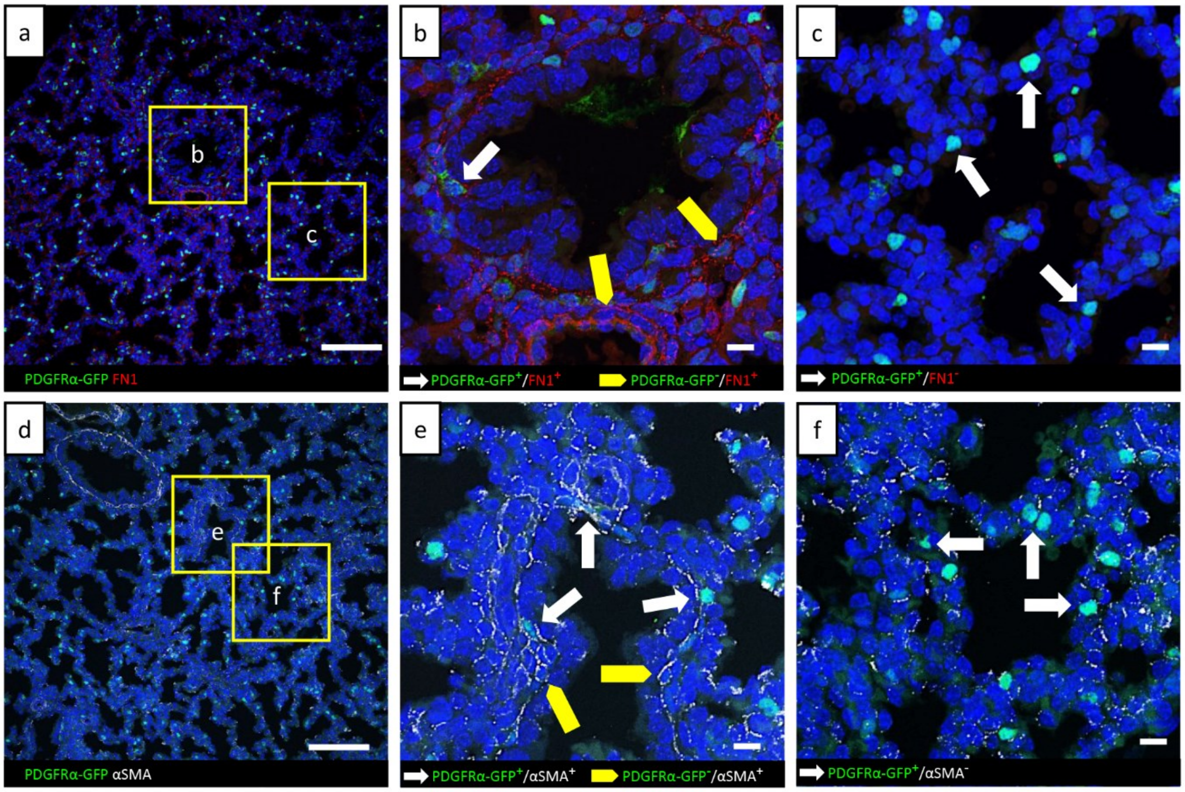

Supplementary Figure 10. Validation of mesenchymal cell subtypes identified by Drop-seq analysis. PDGFRα-GFP$^+$ fibroblasts are sub-divided into multiple populations in the saccular mouse lung, as indicated in the single-cell RNA-seq (scRNA-Seq) analysis. Fibronectin 1 (FN1), a selective marker for MatrixFB-1 cell type at PND1, is localized in E18.5 PDGFRα-GFP$^+$ peribronchiolar matrix fibroblasts (**a**, **b**). The majority of PDGFRα-GFP$^+$ fibroblasts in the terminal saccules are FN1$^-$ at this time (**c**). Some peribronchiolar and perivascular fibroblasts are FN1$^+$/PDGFRα-GFP$^-$, further illustrating the heterogeneity of fibroblasts at this time point (**b**). αSMA (ACTA2) is expressed in the Smooth Muscle, Pericyte-1, and MyoFB-2 scRNA-Seq clusters at PND1, and are visible via immunostaining at E18.5 (**d**). Peribronchiolar αSMA$^+$/PDGFRα-GFP$^+$ MyoFB-2 fibroblasts are present and express dim levels PDGFRα-GFP, as previously noted in Chen et al., 2012[4] (**e**). The majority of PDGFRα-GFP$^+$ fibroblasts in the terminal saccules at this time are αSMA$^-$ (**f**), and the majority of peribronchiolar smooth muscle cells are αSMA$^+$/PDGFRα-GFP$^-$ (**e**). PDGFRα-GFP$^+$ fibroblasts were from five E18.5 mouse embryos. Red: FN1; Green: PDGFRα-GFP; White: αSMA; Blue: DAPI. Scale bars are 100 μm for images acquired with a 20x objective (**a**, **d**) and 10 μm for images acquired with a 60x objective (**b**, **c**, **e**, **f**).

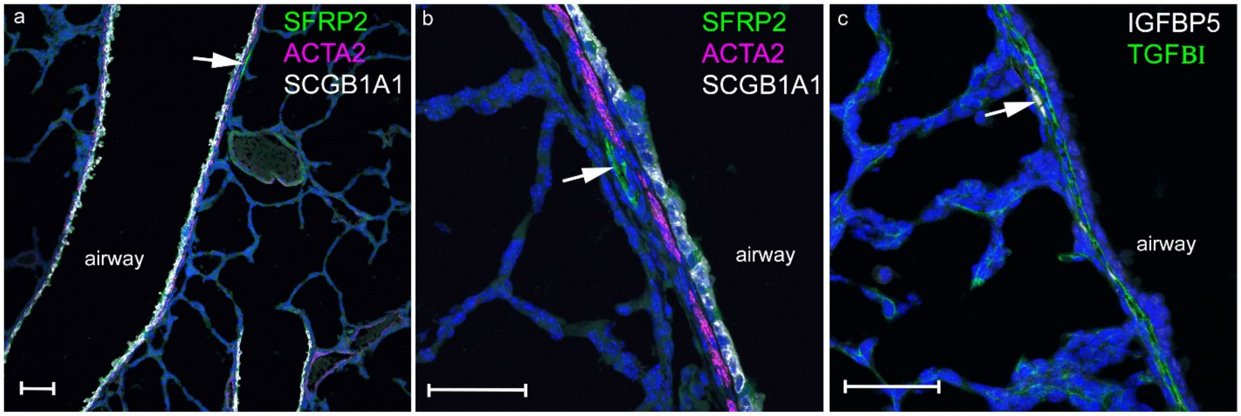

Supplementary Figure 11. Distribution of SFRP2$^+$/IGFBP5$^+$ MatrixFB-2 cells in PND1 mouse lung. SFRP2$^+$ and/or IGFBP5$^+$ mesenchyme cells (white arrows) were distributed only along the airways (**a-c**). SFRP2$^+$ cells were ACTA2$^-$ but were adjacent to ACTA2$^+$ cells (**b**). IGFBP5$^+$ cells were adjacent to cells expressing high levels of TGFBI (**c**). Figures are representative of at least 3 individual mice. Scale bars are 50 μm.

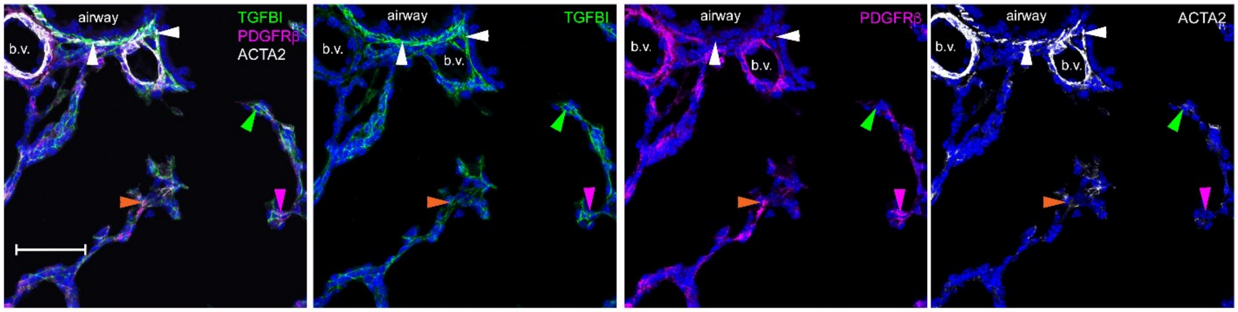

Supplementary Figure 12. Immunostaining for TGFBI in PND1 mouse lung. A magenta arrowhead denotes a cell positive for PDGFRβ only in the alveolus. The green arrowhead indicates a cell only positive for TGFBI located in the distal lung. TGFBI single positive cells were also detected along the airways. White arrowheads mark cells double positive for TGFBI and ACTA2 that are found lining airways representing the smooth muscle cell population identified by Drop-seq. Orange arrowheads indicate triple positive cells in the alveolus, the myofibroblast population. “b.v.” stands for blood vessels. Figures are representative of at least 3 individual mice. Scale bar is 50 μm.

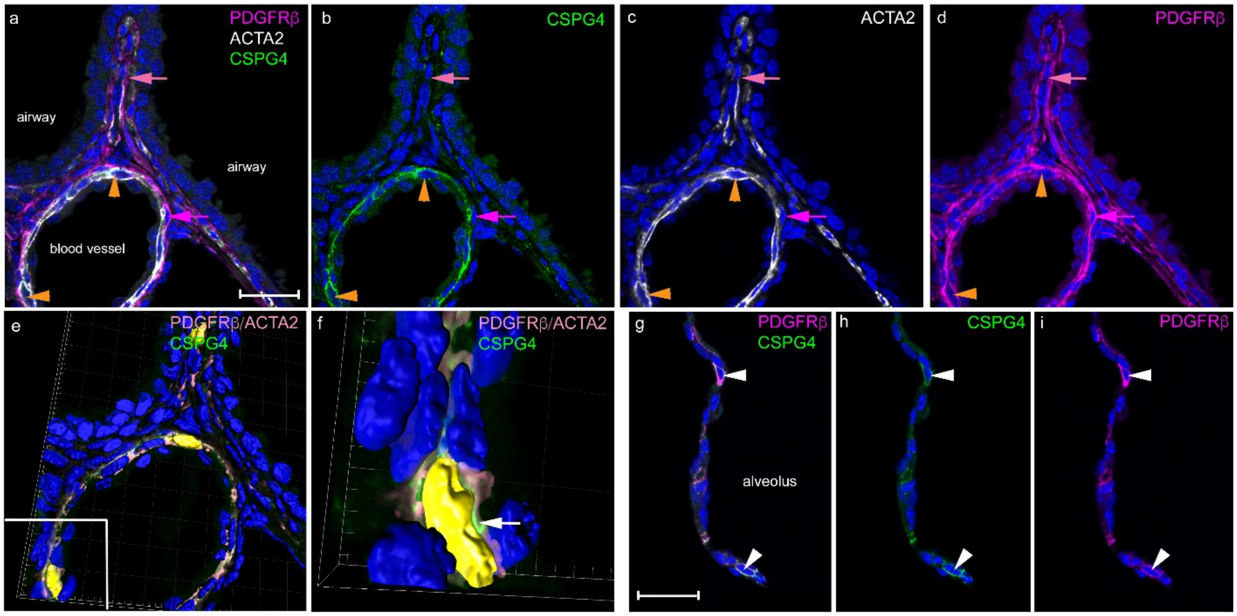

Supplementary Figure 13. Validation of pericyte sub-populations in mouse lung at PND1. PND1 lung tissue was immunostained for the indicated markers and expression visualized by confocal microscopy. (**a-f**) Detection of PDGFRβ^{+}/CSPG4^{+}/ACTA2^{+} cells surrounding blood vessels. (**a-d**) Single plane images taken from a confocal z-stack. Orange arrowheads indicate location of triple positive cells. A cell expressing only PDGFRβ was indicated by magenta arrow. PDGFRβ^{+}/ACTA2^{+} co-expressing cells were also identified adjacent to the airways (pink arrow). (**e-f**) 3-D rendering of the z-stack information was generated using IMARIS (Bitplane) software. The boxed region in (**e**) is enlarged in (**f**). Blue and yellow structures indicate cell nuclei. Using the IMARIS software, the domains of PDGFRβ and ACTA2 co-expression were merged into a light mauve colored structure. Cells with yellow nuclei are surrounded by both PDGFRβ^{+}/ACTA2^{+} staining and CSPG4 expression (white arrow) (**f**). (**g-i**) PDGFRβ^{+}/CSPG4^{+} co-expressing cells were also detected in the lung alveolus. At PND1, ACTA2^{+} cells are rarely identified in the alveolus. Scale bars are 25 μm. Figures are representative of at least 3 individual mice.

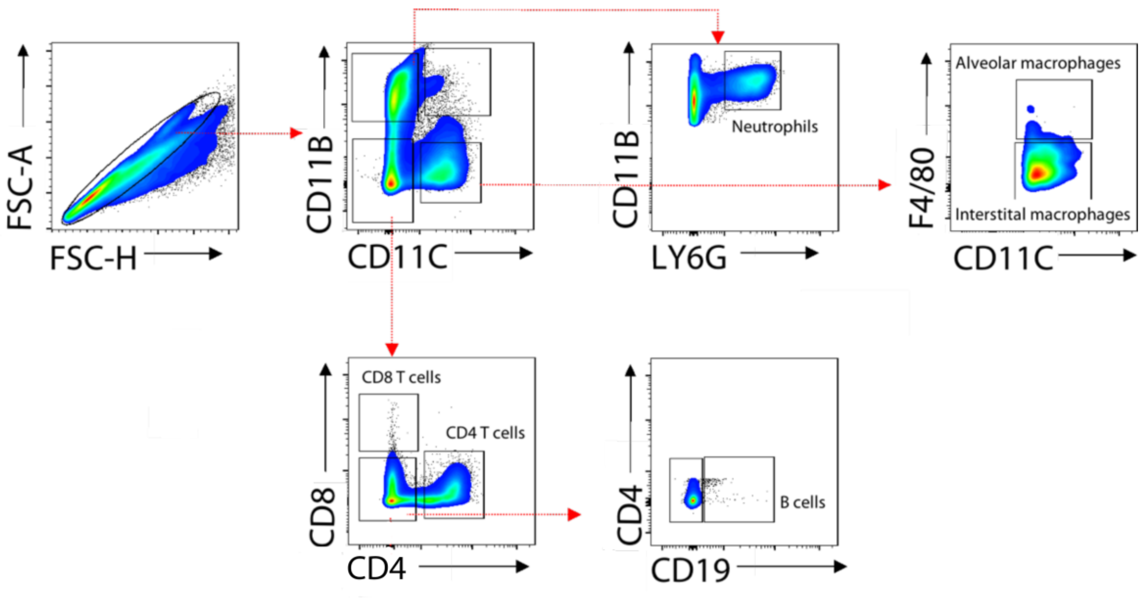

Supplementary Figure 14. Representative bivariate density plots of CD45+ cells in PND1 B6 newborn mice. Flow cytometry analysis identified macrophage, T cells, and B cells in mouse lung at PND1. Macrophages are identified as $CD45^{+}CD11B^{-}CD11C^{+}$ cells, $CD4^{+}$ T-cells are identified as $CD45^{+}CD11b^{-}CD11C^{-}CD4^{+}CD8^{-}$ cells, B cells are identified as $CD45^{+}CD11B^{-}CD11C^{-}CD4^{-}CD8^{-}CD19^{+}$cells. Data are representative of two independent experiments.

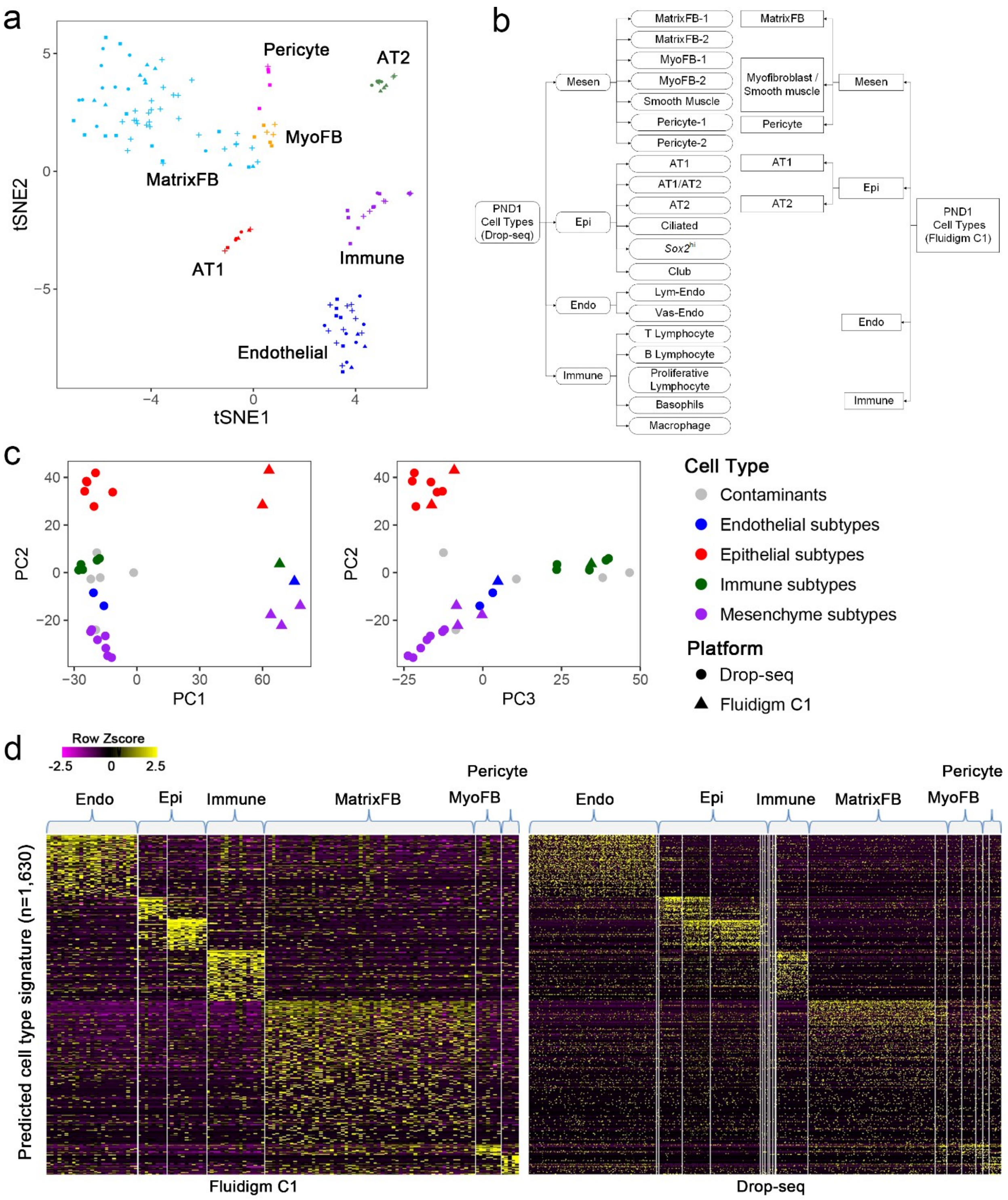

Supplementary Figure 15. Cross-validation and comparison of Drop-seq and Fluidigm C1 single cell RNA-seq analyses of mouse lung at PND1. (**a**) Lung cell types at PND1 identified using Fluidigm C1 single cells (n=130) are shown. Cells were visualized in a two-dimensional tSNE plot. AT1: alveolar type 1 cells; AT2: alveolar type 2 cells; MatrixFB: matrix fibroblast cells; MyoFB: myofibroblast/smooth muscle cells; Endo: endothelial cells; Epi: Epithelial cells;

Immune: immune cells. (**b**) A comparison of cell types identified from Drop-seq and Fluidigm C1 based single cell RNA-seq (scRNA-seq) of mouse lung at PND1. (**c**) Principal component analysis (PCA) of cell populations predicted using Drop-seq and Fluidigm C1 scRNA-seq. The expression of a gene in a cell type was represented by its average normalized expression in the cells of this type. (**d**) Cell type signature genes (n=1,630) predicted from Fluidigm C1 data were selectively expressed in corresponding Drop-seq cell types.

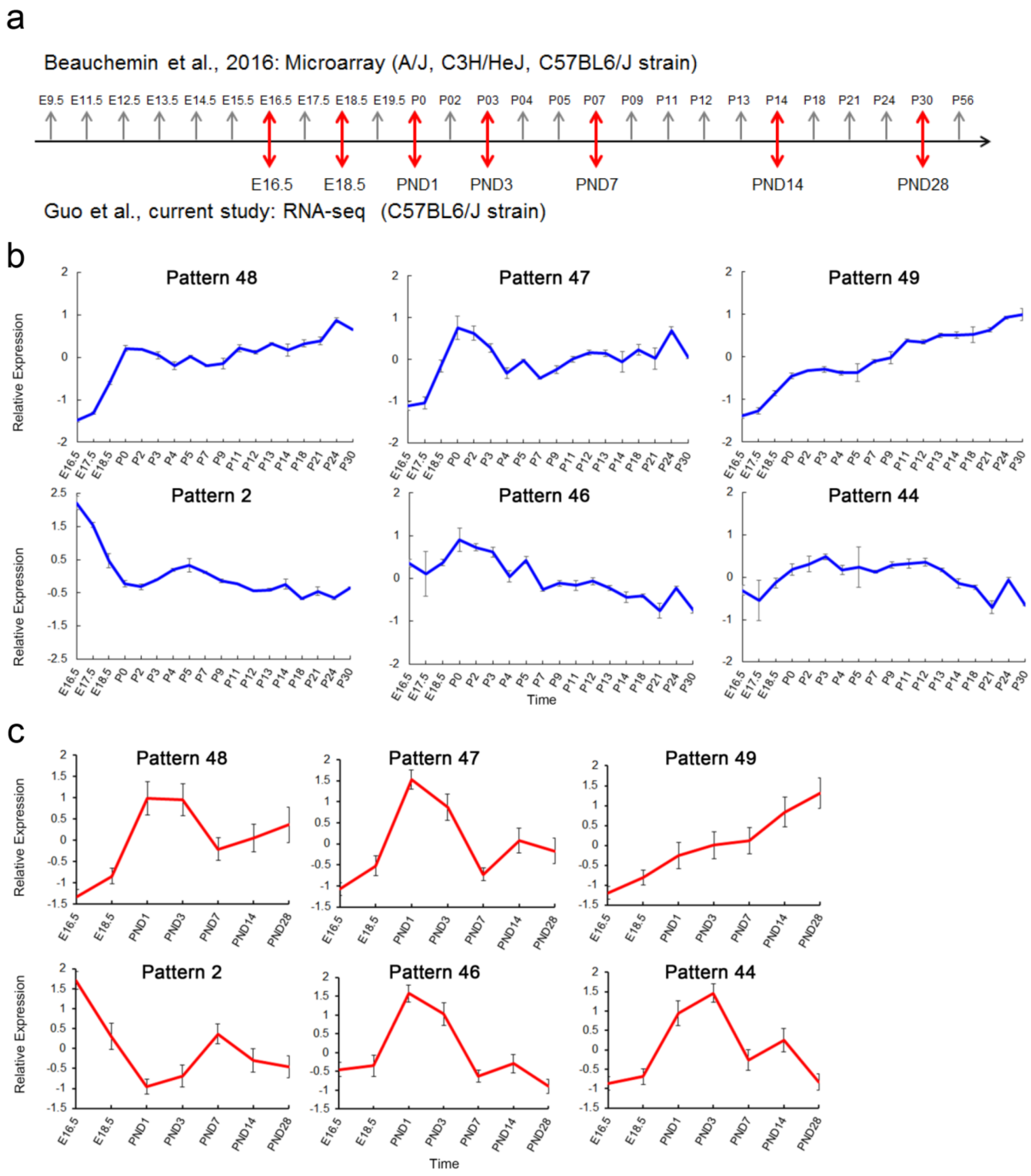

Supplementary Figure 16. Validation of significant temporal patterns using RNA Microarray lung development transcriptome data. (**a**) Whole-lung RNA-seq samples in the present study and the microarray transcriptome data collected in Beauchemin et al., 2016[5]. (**b-c**) showed the expression of six identified temporal patterns in microarray data[5] and RNA-seq data (present study), respectively. In **b**, blue lines represent mean expression, and error bars represent standard error of the mean. In **c**, red lines represent mean expression, and error bars represent standard error of the mean.

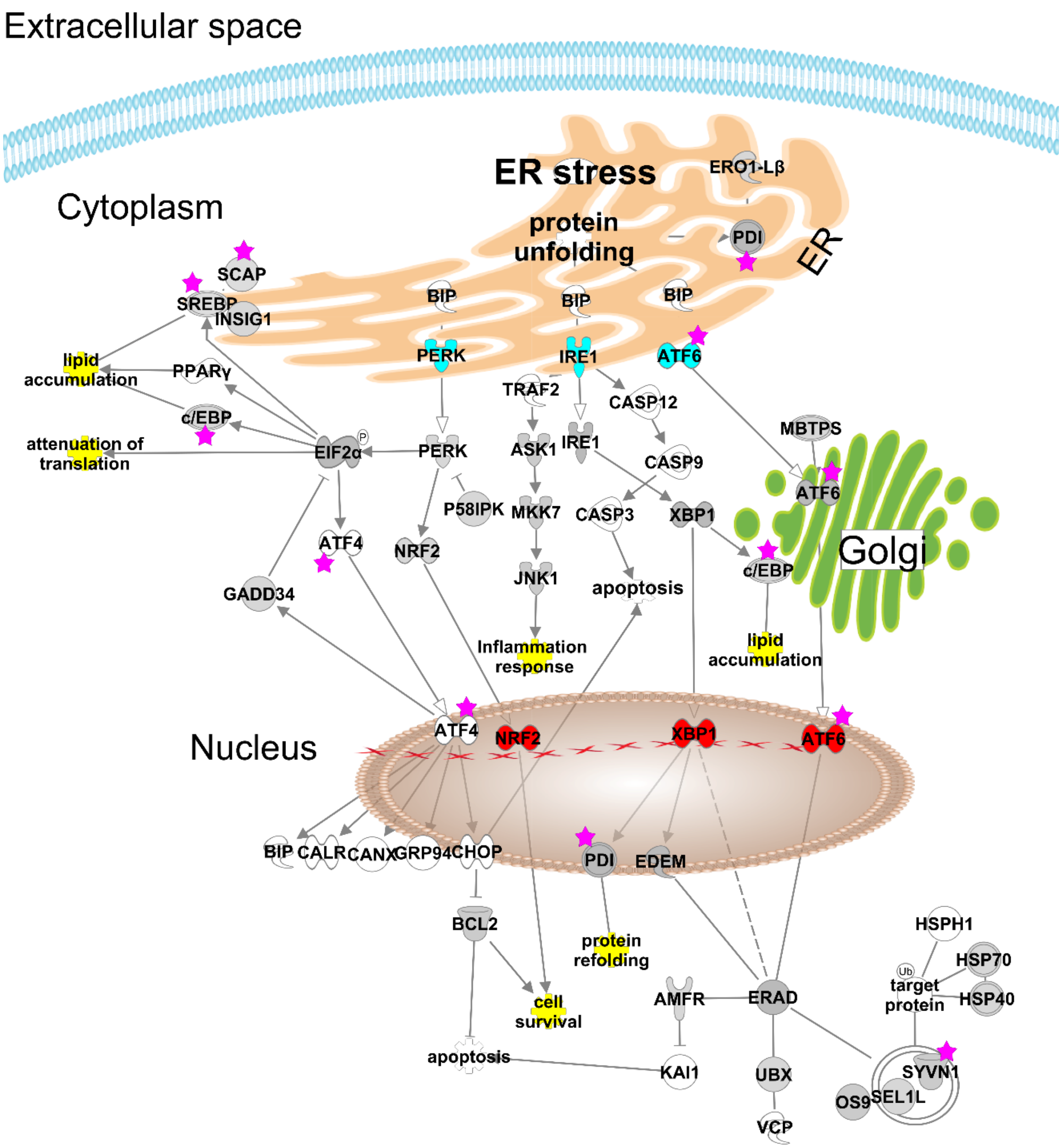

Supplementary Figure 17. Induction of UPR pathway in mouse lung at PND1. Representation of mRNAs induced in modified Ingenuity UPR pathway in mouse lung at birth. Color filled nodes represent genes with peak expression on PND1-PND3 based on the time-course RNA-seq analysis. White nodes indicate genes not induced at birth. Nodes in light blue represent genes encoding ER-stress sensors. Nodes in red represent key transcription factors of UPR signaling pathway. Nodes in grey represent all other genes induced in the UPR pathway. Nodes in yellow represent induced bioprocesses following the induction of UPR pathway. Nodes marked with pink star were studied in the western blotting, qPCR, or immunohistochemistry analyses in **Figs. 6-7 and Supplementary Fig. 18**.

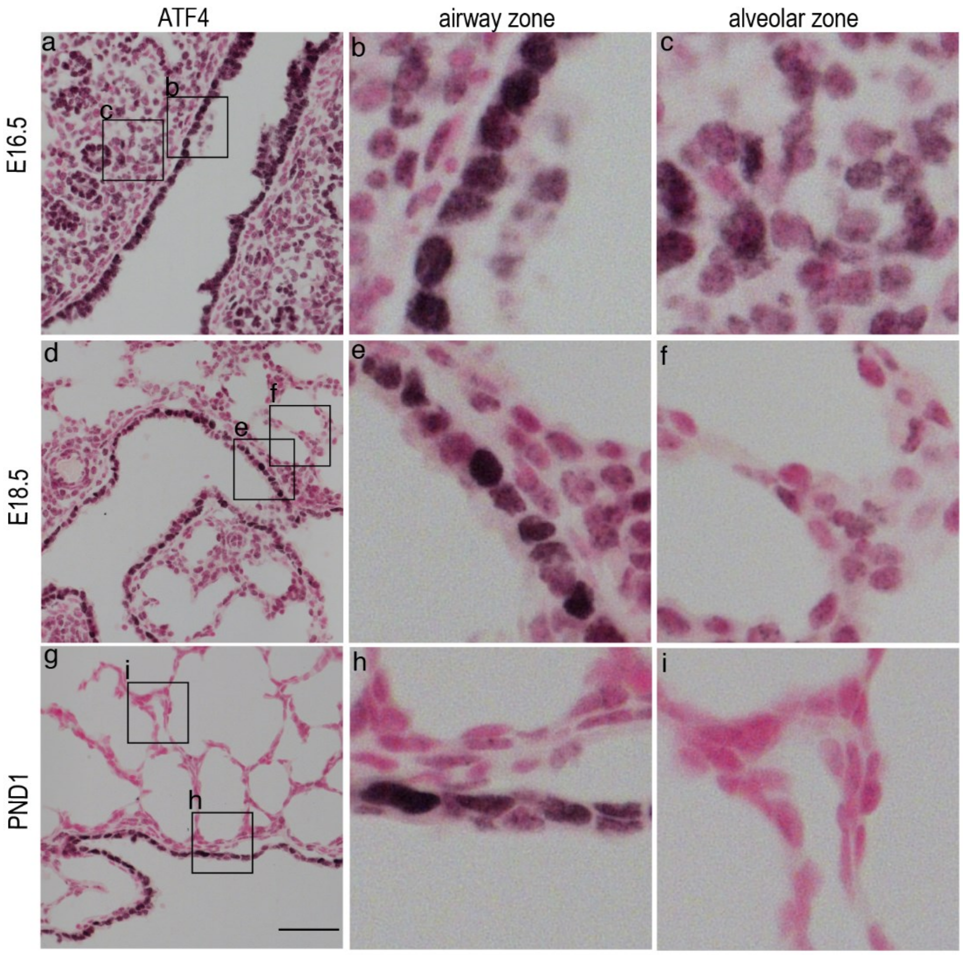

Supplementary Figure 18. ATF4 localization in perinatal mouse lung. ATF4 immunohistochemistry by DAB amplification of embryonic (panels **a-f**) and postnatal day (PND) 1 (panel **g-i**) samples is shown. ATF4 signal was observed in airway cells at all perinatal stages examined. However, immunolocalization signal suggested diminished ATF4 localization in alveolar and its surrounding stromal cells at E18.5 and PND1 relative to E16.5 samples. Images are representative of at least three embryos/animals for each time point. For each time point boxed region in first panel is zoomed in to highlight airway and alveolar zone ATF4 localization as indicated. Scale bar is 50 μm.

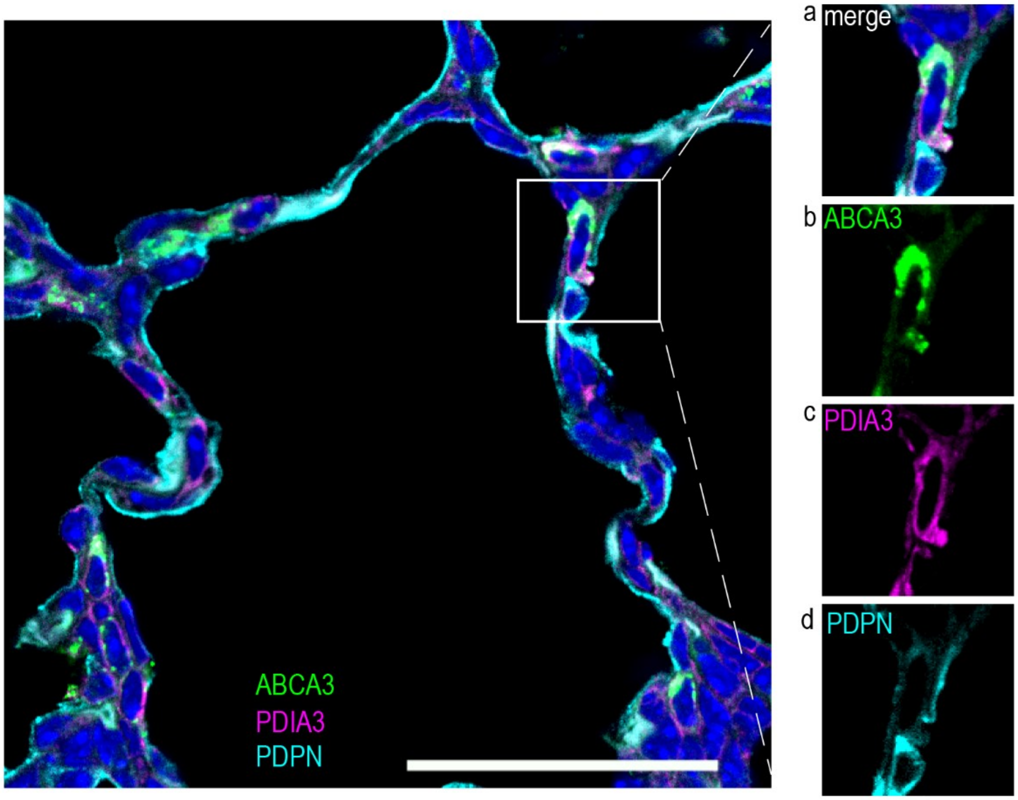

Supplementary Figure 19. PDIA3 was co-localized with ABCA3. Lung tissue (from PND1) was immunostained with PDIA3, ABCA3 and PDPN and imaged by confocal microscopy. PDIA3 was co-localized with ABCA3 (**a-c**) but not with PDPN (**c, d**). Figures are representative of at least 3 individual mice. Scale bar is 50 μm.

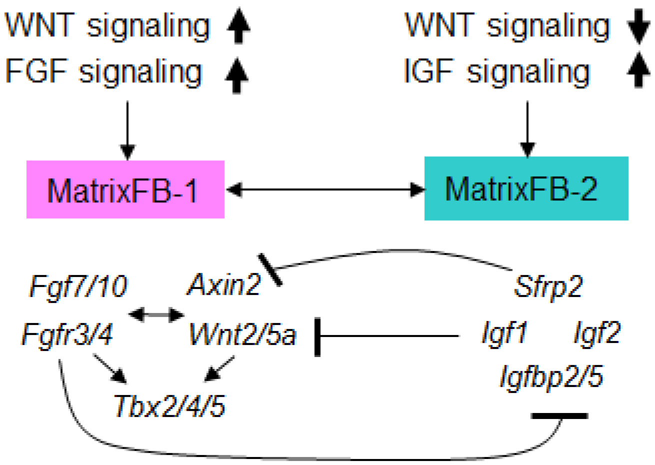

Supplementary Figure 20. Schematic diagram of predicted regulatory network controlling the two matrix fibroblast (MatrixFB) subtypes.

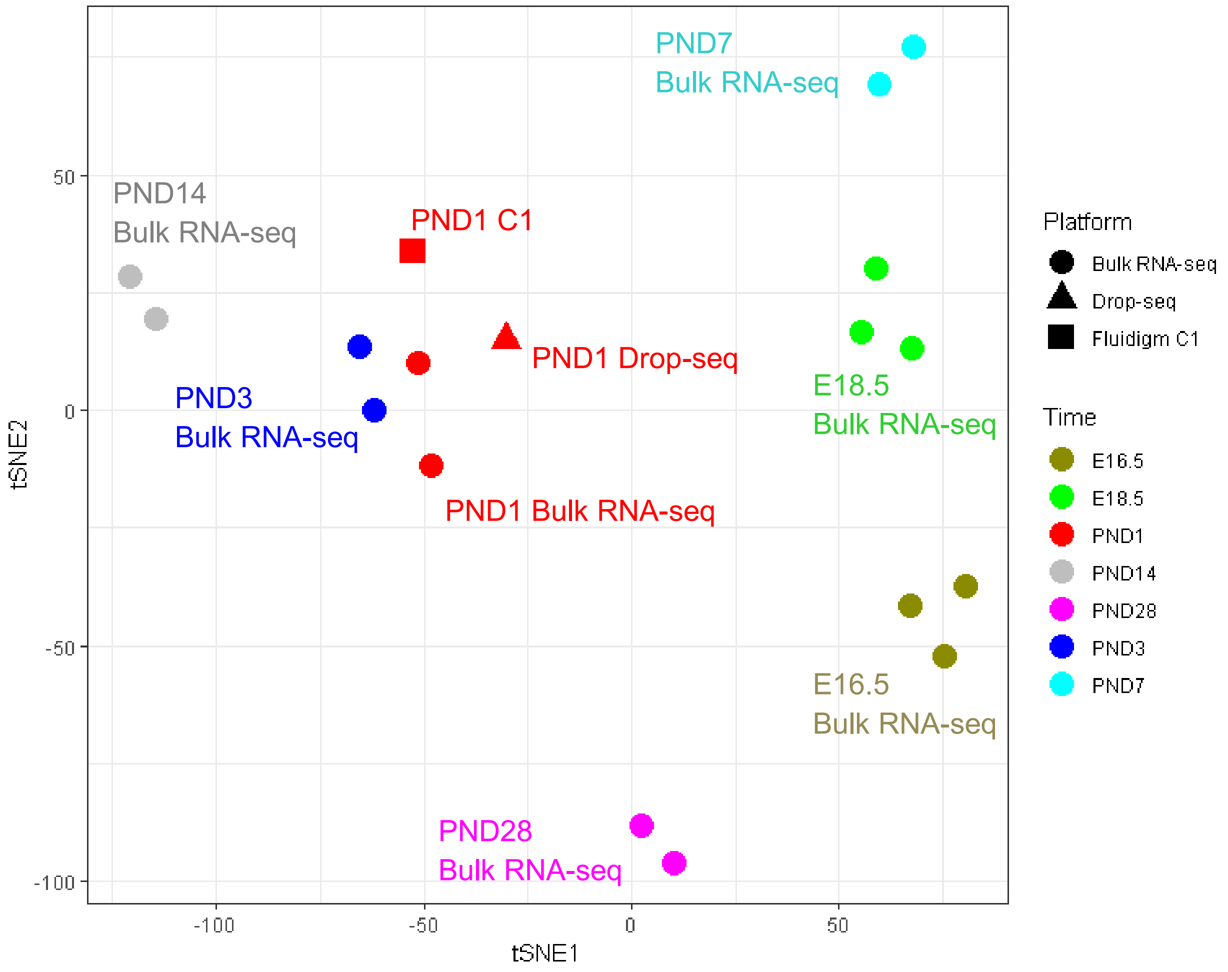

Supplementary Figure 21. PND1 Pseudo-bulk RNA-seq samples were closely located with bulk RNA-seq samples at PND1. "PND1 Drop-seq" pseudo-bulk sample was created by summing UMIs in all PND1 Drop-seq cells for each gene and then was normalized by the total number of UMIs. "PND1 C1" pseudo-bulk sample was created by summing reads in all PND1 Fluidigm C1 cells for each gene and then was normalized by the total number of reads. Principal component analysis was applied to all the 18 samples (16 bulk RNA-seq samples and 2 pseudo-bulk RNA-seq samples) and the first 8 principal components were used for t-Distributed Stochastic Neighbor Embedding (t-SNE) analysis.

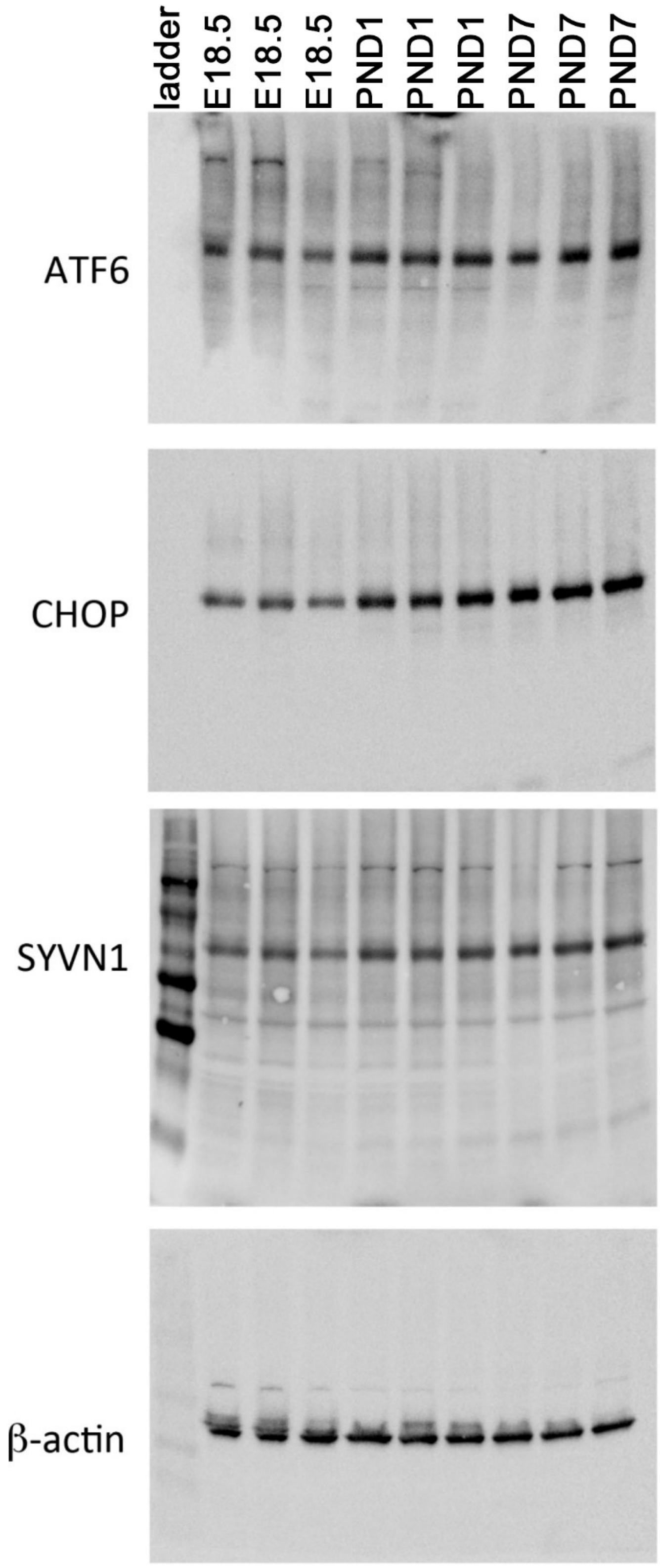

Supplementary Figure 22. Images of full sized immunoblots. Related to Fig. 6.

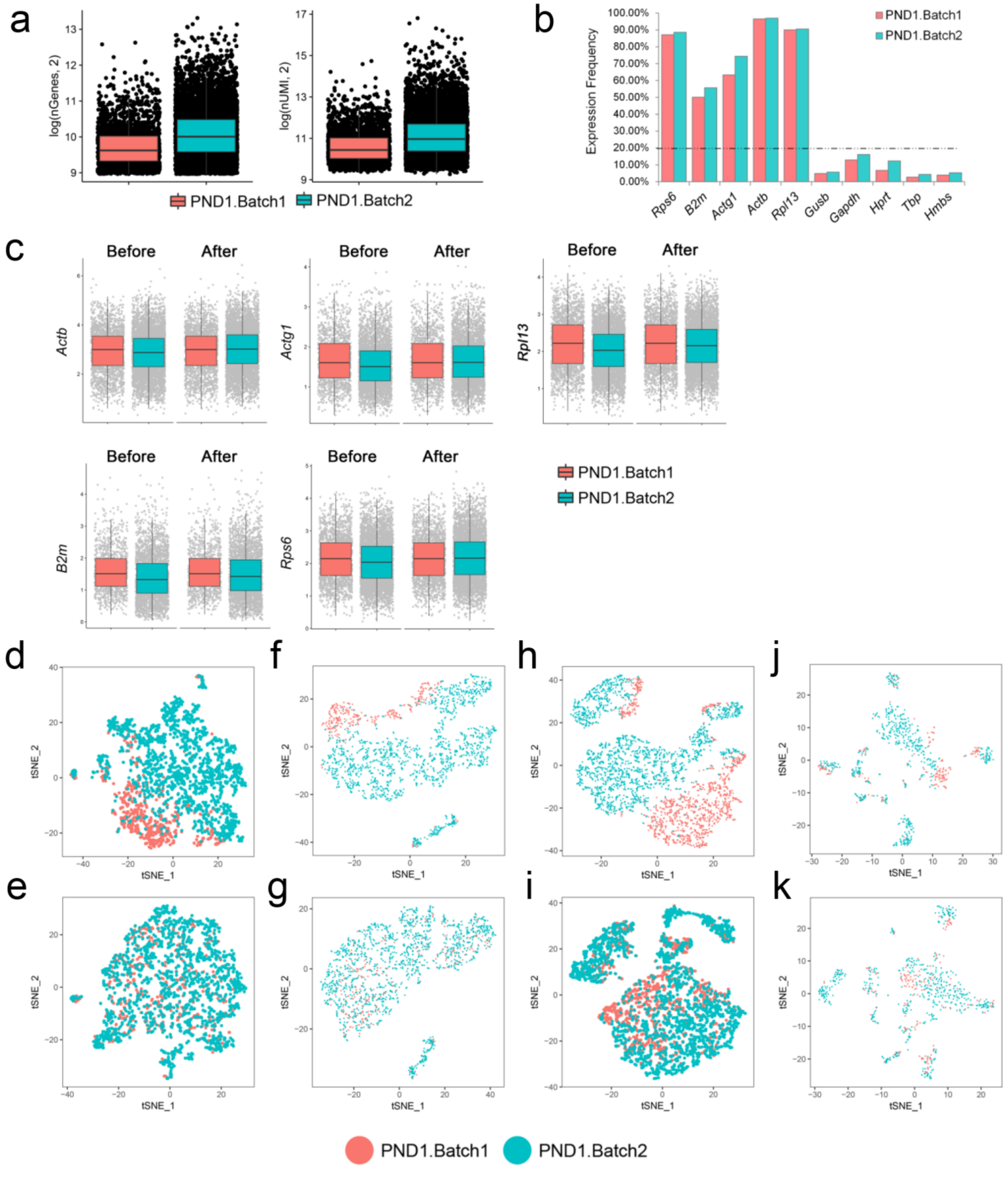

Supplementary Figure 23. Correction of batch difference in cell type identification using Drop-seq of mouse lung at PND1. (**a**) The number of detected genes (nGenes) and transcript counts (nUMI, unique molecular identifier) per cell between the two batches of Drop-seq data. Boxplots represent 25th (bottom), 50th (centerline), and 75th (top) percentiles. (**b**) The expression frequency of commonly used housekeeping genes in the two batches. (**c**) The differences between the median expressions of housekeeping genes were reduced after lung-negative-

gene based scaling of gene expression. Boxplots represent 25th (bottom), 50th (centerline), and 75th (top) percentiles. (**d**-**e**) t-distributed Stochastic Neighbor Embedding (tSNE) analysis of endothelial cells before and after ComBat[6] correction of gene expression, respectively. (**f**-**g**) tSNE analysis of epithelial cells before and after ComBat correction of gene expression, respectively. (**h**-**i**) tSNE analysis of mesenchymal cells before and after ComBat correction of gene expression, respectively. (**j**-**k**) tSNE analysis of immune cells before and after ComBat correction of gene expression, respectively.

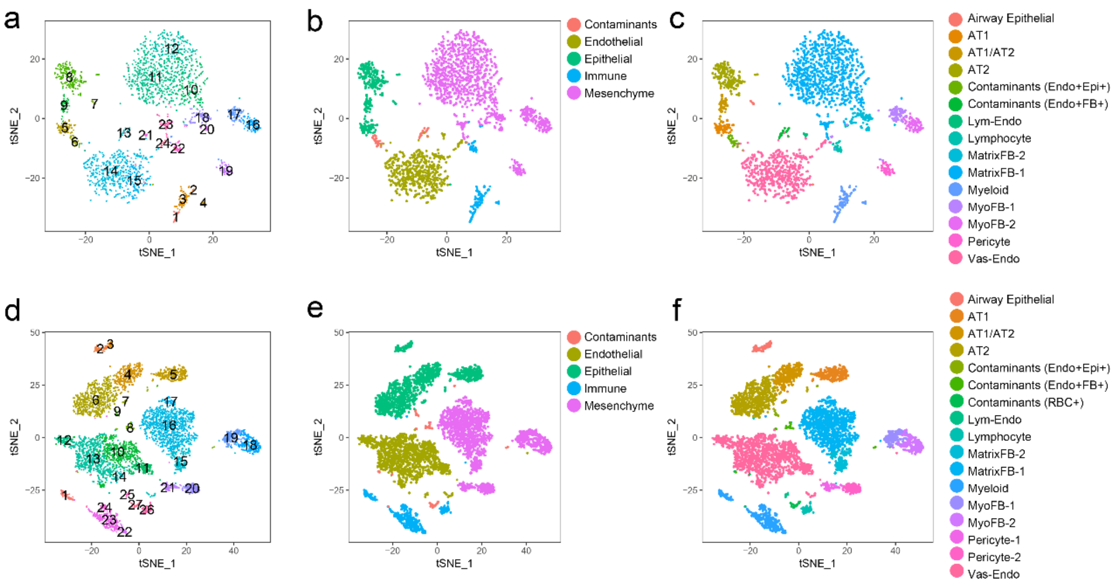

g

Integrated analysis

Independent analysis	Major cell type	Cell subtype	Contam					Endo		Epi						Immune					Mesen						
			Contaminants (Endo+Epi+)	Contaminants (Endo+FB+)	Contaminants (Immune+FB+)	Contaminants (Platelet+)	Contaminants (RBC+)	Vas-Endo	Lym-Endo	AT1	AT1/AT2	AT2	Club	Sox2hi	Ciliated	Proliferative Lymphocyte	B Lymphocyte	T Lymphocyte	Basophils	Macrophage	MatrixFB-1	MatrixFB-2	Pericyte-1	Pericyte-2	MyoFB-1	MyoFB-2	Smooth Muscle
PND1.Batch1	Contam	Contaminants (Endo+Epi+)	29	0	0	0	0	0	0	0	0	0	0	0	0	0	0	0	0	0	0	0	0	0	0	0	0
		Contaminants (Endo+FB+)	0	19	0	0	0	0	0	0	0	0	0	0	0	0	0	0	0	0	1	0	0	0	0	0	0
	Endo	Vas-Endo	3	2	0	0	1	559	0	0	0	0	0	0	0	0	0	0	0	0	0	0	0	0	1	0	0
		Lym-Endo	0	0	4	0	0	2	13	0	0	0	0	0	0	1	0	0	0	0	0	0	0	0	0	0	0
	Epi	AT1	0	0	0	0	0	0	0	71	3	0	0	0	0	0	0	0	0	0	0	0	0	0	0	0	0
		AT1/AT2	0	0	0	0	0	0	0	9	41	3	0	0	0	0	0	0	0	0	0	0	0	0	0	0	0
		AT2	0	0	3	0	0	0	0	0	6	178	0	0	0	0	0	0	0	0	2	0	0	0	0	0	0
		Airway Epithelial	0	0	0	0	0	1	0	0	0	0	4	1	8	0	0	0	0	0	0	0	0	0	0	0	0
	Immune	Lymphocyte	0	0	0	0	1	2	0	0	0	0	0	0	0	5	22	7	0	0	1	1	1	0	0	0	0
		Myeloid	0	0	10	1	0	0	0	0	0	0	0	0	0	3	0	1	5	118	0	0	0	0	0	1	0
	Mesen	MatrixFB-1	0	3	0	0	3	0	0	2	0	0	0	0	0	0	1	1	0	0	896	14	0	0	1	3	0
		MatrixFB-2	0	0	0	0	0	0	0	0	0	0	0	0	0	0	0	0	0	0	3	80	9	0	0	1	1
		Pericyte	0	0	0	0	0	3	0	0	0	0	0	0	0	0	0	0	0	0	0	0	0	58	0	0	0
		MyoFB-1	0	0	0	0	1	0	0	0	0	0	0	0	0	0	0	0	0	0	0	0	0	0	49	31	1
		MyoFB-2	0	0	0	0	0	0	0	0	0	1	0	0	0	0	0	0	0	0	0	0	0	0	0	32	50
PND1.Batch2	Contam	Contaminants (Endo+Epi+)	9	0	0	0	0	1	0	0	3	6	0	0	0	0	0	0	0	0	0	0	0	0	0	0	0
		Contaminants (Endo+FB+)	0	24	0	0	0	10	0	0	0	0	0	0	0	0	0	0	0	0	1	1	0	0	1	1	0
		Contaminants (RBC+)	0	0	6	0	18	16	0	2	0	2	0	0	0	1	0	0	0	1	0	0	0	0	0	0	0
	Endo	Vas-Endo	1	0	1	0	0	1511	0	0	0	0	0	0	0	2	0	0	0	0	4	2	3	0	0	1	0
		Lym-Endo	0	0	0	0	0	1	24	0	1	0	0	0	0	0	0	0	0	0	0	0	0	0	0	0	0
	Epi	AT1	0	0	1	0	0	0	0	296	3	0	0	0	0	0	0	0	0	0	0	0	0	0	1	1	0
		AT1/AT2	0	0	0	0	0	0	0	5	322	5	0	0	0	0	0	0	0	0	0	0	0	0	0	0	0
		AT2	0	0	1	0	0	0	0	8	80	629	1	0	0	0	0	0	0	0	1	0	0	0	0	1	0
		Airway Epithelial	0	0	1	0	0	0	0	0	1	2	36	45	32	1	0	0	0	0	0	0	0	0	0	0	0
	Immune	Lymphocyte	0	0	1	3	0	0	0	0	0	1	0	0	0	11	33	29	2	1	0	0	0	1	0	0	0
		Myeloid	0	0	19	1	0	0	0	2	0	0	0	0	0	4	0	0	7	411	0	0	0	0	0	0	0
	Mesen	MatrixFB-1	0	1	1	0	0	0	0	0	0	0	0	0	0	0	0	0	0	0	1099	0	0	0	0	0	0
		MatrixFB-2	0	0	0	0	0	0	0	0	0	0	0	0	0	0	0	0	0	0	101	102	1	0	0	2	1
		Pericyte-2	0	0	3	0	0	0	0	0	0	0	0	0	0	0	0	0	0	0	0	2	83	8	1	2	0
		Pericyte-1	0	0	0	0	0	2	0	0	0	0	0	0	0	0	0	0	0	0	0	0	0	143	0	0	0
		MyoFB-1	0	0	0	0	0	2	0	0	0	0	0	0	0	0	0	0	0	0	0	0	0	0	163	9	0
		MyoFB-2	0	0	0	0	0	0	0	0	0	0	0	0	0	0	0	0	0	0	0	0	1	0	22	151	62

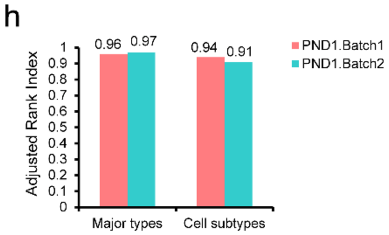

Supplementary Figure 24. Consistency in cell type identification between independent analysis and integrated analysis of the two batches of Drop-seq data. (**a-c**) Independent cell type identification analysis of batch 1 of postnatal day 1 (PND1) mouse lung Drop-seq data. (**a**) Unbiasedly identified cell clusters in batch 1. (**b**) Putative major cell types in batch 1. (**c**) All putative cell types in batch 1. (**d-f**) Independent cell type identification analysis of batch 2 of PND1 mouse lung Drop-seq data. (**d**) Unbiasedly identified cell clusters in batch 2. (**e**) Putative major cell types in batch 2. (**f**) All putative cell types in batch 2. (**g**) Overlaps between independent and integrated cell type assignment of PND1 mouse lung Drop-seq data. Red color represents row max value. (**h**) The adjusted rank index of major cell type and cell subtype assignment in independent and integrated analysis of the two batches of PND1 mouse lung Drop-seq data. The cell type identification in the independent analysis of individual batches was largely followed the analytic workflow in the integrated analysis described in the "Computational analysis of Drop-seq". Briefly, in the independent analyses of each batch, genes detected in less than 2 cells were excluded from the analysis; "MeanVarPlot" function in Seurat[7] was used for detecting highly variable genes, which were then used for principal component analysis (PCA) based dimension reduction; top 20 principal components (PCs) were used for t-distributed Stochastic Neighbor Embedding (tSNE) and clustering analysis. Cell clusters were identified using Louvain-Jaccard algorithm[1] using the scores of top 20 PCs as input. Cell type assignment was based on inspecting the expression of known cell type markers. Adjusted Rank Index values were calculated using the "adjustedRandIndex" function in R mclust package.

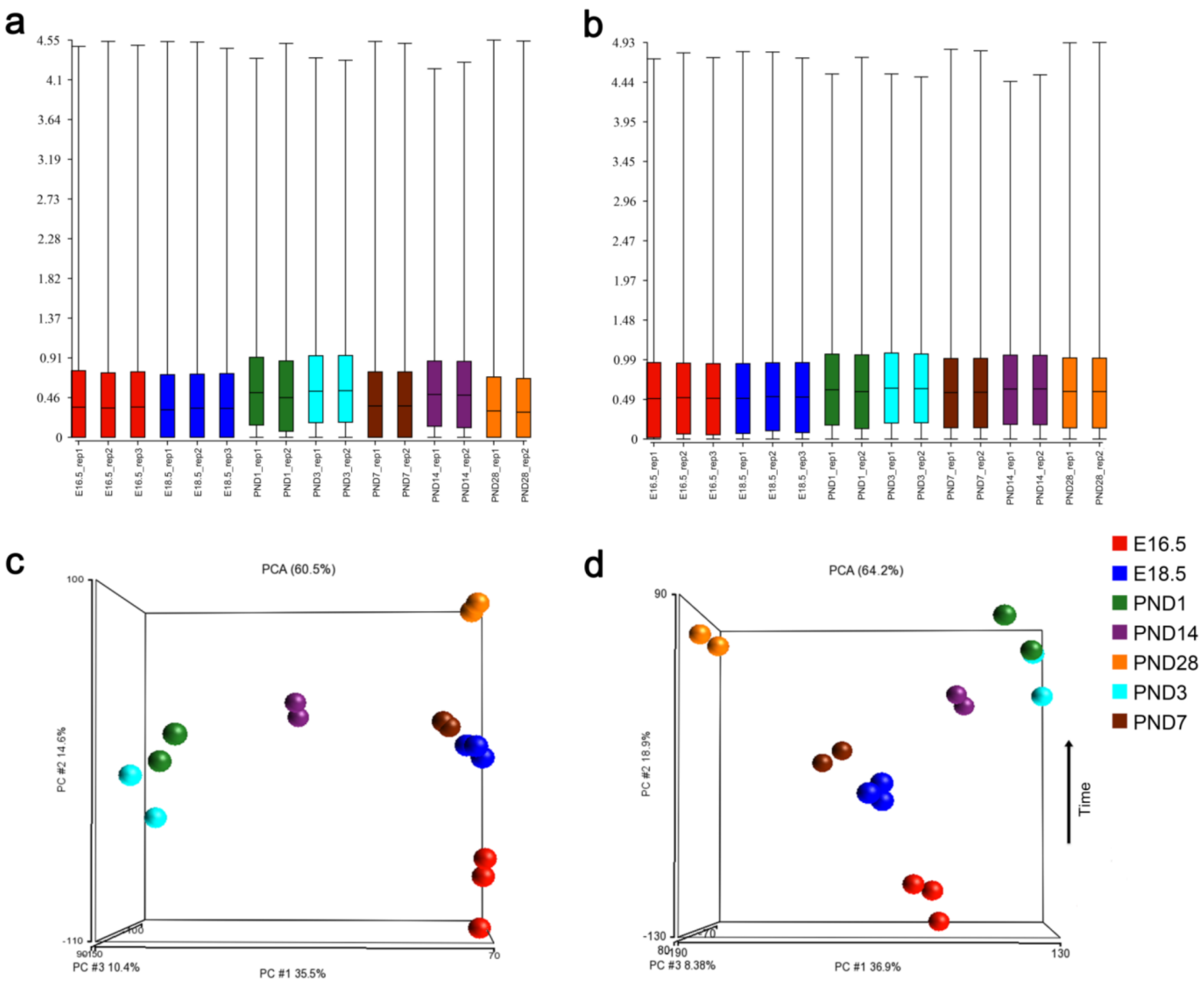

Supplementary Figure 25. Normalization of time-course RNA-seq data to remove batch difference. (**a**-**b**) showed the boxplot of gene expression profiles of time-course RNA-seq samples before and after lung-negative gene based normalization, respectively. (**c**-**d**) showed the principal component analysis (PCA) of time-course RNA-seq samples before and after normalization.

Supplementary Notes

Supplementary Note 1. Computational analysis of time-course RNA-seq data.

Since the experiments and sequencing data were generated in different times (**Supplementary Fig. 25, Supplementary Table 2**), boxplot and principal component analysis (PCA) detected the batch effect as primary source of variation **Supplementary Fig. 25a and 25c**. Three-dimensional scatter plot of the first three PCA components in **Supplementary Fig. 25c** shows PC1 (principal component 1) separated the samples by the batch effects and PC2 separated the samples by the developmental time stages.

We applied a list of lung specific negative control genes to normalize gene expression among batches. Negative control genes were selected based on following three criteria: 1) the expression of the gene is not significantly changed in any of the lung perturbation experiments in Pulmonary Biology Gene Expression Database (https://research.cchmc.org/pbge/index.html); 2) the expression of the gene is not significantly changed during the lung developmental times[5, 8, 9]. 2,677 genes meet the criteria were used to normalize gene expression from three difference batches. **Supplementary Fig. 25b** and **25d** showed that negative control genes normalization significantly reduced batch effects and samples are clustered by their development time. After normalization, genes with >=3 expression in at least one sample and >=20% coefficient of variation across all time points were used in the downstream analysis. STEM (Short Time-series Expression Miner)[10] was applied to discover significant temporal patterns using the expression profiles of differentially expressed genes. Six significant patterns were identified with *p* value <0.05 and cluster size > 300 genes. In total, 3716 genes were assigned to these 6 major patterns (**Fig. 5**). The temporal expression patterns for individual genes, as well as the six significant temporal profiles, can be queried and visualized in

https://research.cchmc.org/pbge/lunggens/SCLAB.html. Functional enrichment analysis of temporal profiles was performed using ToppGene Suite (http://toppgene.cchmc.org/)[11].

Supplementary Note 2. Comparison of cell type assignments between integrated and independent analysis.

Multiple batch correction operations (lung-negative-gene based expression scaling, batch-aware highly variable gene selection, ComBat[6] based expression correction for dimension reduction) were applied in the above integrated cell type assignment of 8,003 cells from the two batches. To assess whether these correction operations affected the cell type assignment, we performed independent cell type assignment using the data from individual batches only, and compared the cell type assignments from the integrated and independent analyses. The results showed that the integrated and independent cell type assignment (at both major cell type and subtype levels) have high consistency (>0.9 adjusted rank index, **Supplementary Fig. 24**), suggesting the correctness of our batch correction operations and the cell type assignments from the integrated analysis.

Supplementary References

1. Shekhar K*, et al.* Comprehensive Classification of Retinal Bipolar Neurons by Single-Cell Transcriptomics. *Cell* **166**, 1308-1323 e1330 (2016).

2. Qiu X*, et al.* Reversed graph embedding resolves complex single-cell trajectories. *Nature methods* **14**, 979-982 (2017).

3. Guo M, Bao EL, Wagner M, Whitsett JA, Xu Y. SLICE: determining cell differentiation and lineage based on single cell entropy. *Nucleic acids research* **45**, e54 (2017).

4. Chen L, Acciani T, Le Cras T, Lutzko C, Perl AK. Dynamic regulation of platelet-derived growth factor receptor alpha expression in alveolar fibroblasts during realveolarization. *American journal of respiratory cell and molecular biology* **47**, 517-527 (2012).

5. Beauchemin KJ*, et al.* Temporal dynamics of the developing lung transcriptome in three common inbred strains of laboratory mice reveals multiple stages of postnatal alveolar development. *PeerJ* **4**, e2318 (2016).

6. Johnson WE, Li C, Rabinovic A. Adjusting batch effects in microarray expression data using empirical Bayes methods. *Biostatistics* **8**, 118-127 (2007).

7. Satija R, Farrell JA, Gennert D, Schier AF, Regev A. Spatial reconstruction of single-cell gene expression data. *Nature biotechnology* **33**, 495-502 (2015).

8. Xu Y*, et al.* Transcriptional programs controlling perinatal lung maturation. *PLoS One* **7**, e37046 (2012).

9. Kho AT, Bhattacharya S, Mecham BH, Hong J, Kohane IS, Mariani TJ. Expression profiles of the mouse lung identify a molecular signature of time-to-birth. *Am J Respir Cell Mol Biol* **40**, 47-57 (2009).

10. Ernst J, Bar-Joseph Z. STEM: a tool for the analysis of short time series gene expression data. *BMC bioinformatics* **7**, 191 (2006).

11. Chen J, Bardes EE, Aronow BJ, Jegga AG. ToppGene Suite for gene list enrichment analysis and candidate gene prioritization. *Nucleic acids research* **37**, W305-311 (2009).
