# Supplementary material for: Single cell RNA analysis identifies cellular heterogeneity and adaptive responses of the lung at birth
Source: Nat Commun. 2019 Jan 3;10:37. doi: 10.1038/s41467-018-07770-1 (PMC6318311; doi:10.1038/s41467-018-07770-1)
Supplement: Supplementary file 3 — Description of Additional Supplementary Files [file 41467_2018_7770_MOESM3_ESM.pdf]

Supplementary Data 1. Putative cell type assignment for PND1 Drop-seq data.

Supplementary Data 2. Predicted signature of PND1 Drop-seq cell types and enriched functional annotations.

Supplementary Data 3. Putative cell type assignment for PND1 Fluidigm C1 single cell RNA-seq data.

Supplementary Data 4. Predicted cell type signature genes using PND1 Fluidigm C1 single cell data.

Supplementary Data 5. Temporal patterns and their enriched functions of time-course whole lung RNA-seq data.

Supplementary Data 6. Associations of Unfolded Protein Response (UPR) network genes with cell types identified from Drop-seq analysis of mouse lung on postnatal day 1 (PND1). The calculations of sensitivity based “Enrichment” and “*p* value” of Fisher’s exact test were described in the Methods section. Shown are associations with *p* value<0.05 and Enrichment score>=1.5. Sensor: stress sensors; TF: transcription factors; ERAD: endoplasmic reticulum associated degradation; Lipid: lipid biosynthesis; Other: other genes in UPR network. Minimum “*p* value” was set to 1.00E-05.

Source Data 1. Source data for tSNE plot of PND1 Drop-seq cells in Figure 1.

Source Data 2. Source data for western blot and qPCR plots in Figure 6.
