# Supplementary material for: Single cell RNA analysis identifies cellular heterogeneity and adaptive responses of the lung at birth
Source: Nat Commun. 2019 Jan 3;10:37. doi: 10.1038/s41467-018-07770-1 (PMC6318311; doi:10.1038/s41467-018-07770-1)
Supplement: Supplementary file 11 — Source Data [file 41467_2018_7770_MOESM11_ESM.pdf]

Reviewers' Comments:

Reviewer #1:
Remarks to the Author:
In this manuscript, the authors perform sc-RNAseq using droplet-based technology on PNd1 mouse lung. They use unsupervised clustering to distinguish known populations and sub-populations and validate a subset in tissue by PLISH and antibody staining. They perform other statistical analyses including an entropy analysis, infer Notch pathway activation in cell types that recapitulates known biology, and highlight predicted transcription factors in lymphatic and vascular endothelial cells that 'rediscover' known regulators of these cell types. They go on to perform pathway analysis and map gene cohorts that track together in abundance over time by analysis in RNAseq results obtained at specific developmental and postnatal stages.

Overall the dataset and accompanying user interface will provide a valuable resource for the lung biology community. There are also some novel and interesting observations such as the identification and validation of the activated UPR at PNd1. Some portions of the results are less well-developed and occasionally surprising in relation to known biology, as detailed below.

MAJOR COMMENTS
1) In some cases, molecularly distinct sub-classes are proposed to be functionally distinct sub-types (e.g., FB1 and FB2 or Per1 and Per2) while in other cases they are proposed to be the same cell type at distinct stages of maturation (e.g., BP, Sox2-Hi, MyoFB). While these interpretations have been well-established by prior work for BPs (and somewhat for FBs), they are less well-supported by prior knowledge for the other 3 instances. The authors should provide further evidence from the literature or their own data to support the presence of two distinct pericyte sub-types. Similarly, the rationale for why they believe the MyoFB classes to represent early versus late (rather than 2 distinct sub-types) is not clearly explained.
The most surprising is the proposal that Sox2-Hi cells are the progenitors for Club and ciliated cells. This Sox2-Hi population expressed markers of both Club and ciliated cells, yet prior work in the field suggests that ciliated cells differentiate much earlier in development, and at least one lineage study using CCSP-Cre has reported that marked cells exclusively give rise to Club cells. Furthermore, Sox2+ airway cells that are neither Club nor ciliated cells exist in mature mouse airways and generate basal cell-like cells that become active following H1N1 influenza. In light of these previous studies, the authors' reliance on co-expression of Club and ciliated markers along with entropy analysis does not seem sufficient to conclude that the Sox2-Hi class are bipotent progenitors of Club and ciliated cells.

2) The sc-RNAseq results indicate Lyve-1 is expressed in both lymphatic and vascular endothelial cells. However, Lyve-1 is believed to be specific for lymphatic endothelial cells based on prior work in the field. How do the authors reconcile this result? Is it possible what they call the vascular endothelial population may represent a different lymphatic endothelial cell type or progenitor?

3) For every named cell class and sub-class, the authors should specify which exact marker(s) they use to identify the named class. For instance, what defines and distinguishes what the authors call myofibroblasts from fibroblasts, pericytes, and smooth muscle cells? To some degree, these classes express the same transcripts, though at different levels. The authors should be very clear about this to allow for comparison of their dataset and conclusions with future studies by different groups.

MINOR POINTS
1) In Figure 9, the wrong color is indicated for the AGER stain
2) There are numerous instances of spelling errors throughout the manuscript

Reviewer #2:
Remarks to the Author:
I found this a very interesting description of the transcriptional profile defining individual cell types in the mouse lung using a Drop-seq technique. The types of cell types identified and their gene expression match very well with previous knowledge but this study adds further knowledge and complexity to the variety of cell types in the lung. RNA Seq is then used to analyse temporal changes in gene expression through embryonic development and after birth, with the transcriptome data being inferred back on the single cell data to explore which cells are likely contributing to key changes in gene expression around birth. Whilst it would have been better to have single cell data at each time point that would have been a heroic effort. The key signatures of adaptation to breathing/birth include responses to oxidative and ER stress, and increased lipid metabolism which are well explained in the discussion. Protein studies are used to confirm these changes, with for example a very convincing enhancement in ATF6 at PND1 in epithelial cells. Perhaps more consideration could be given in the discussion to the need for expansion of cellular ER likely to be required to facilitate enhanced glycoprotein and lipid biosynthesis. Discussion could also be extended as to the possible importance of the increasing expression of many of the key genes (in Fig 6d) prior to birth in preparation for the response to birth, and how that might impact in prematurity.

Minor corrections
P15 line 4 – “endothelial cell” should be “endothelial cells”
P17 line 14 – “similarly” should be “similar”
P41 line 12 “important in regulation” should be “important role in regulation”
For methodology, some further detail on the Drop-seq isolation procedures (currently simply referring to the original Macosko paper in Cell) that may affect gene expression (eg. disaggregation techniques, time to isolate cells) would be useful

Reviewer #3:
Remarks to the Author:
Review to nature communications
Article entitled “Preparing for the first breath at a single cell level”

Summary:
The author’s goal was to identify dynamic changes in gene expression in the diverse pulmonary cells at birth. The authors used single cell gene expression technology to identify murine pulmonary cell types on the day of birth. Epithelial, endothelial, mesenchymal, and immune cells and their subpopulations were identified. The identified cell population and the respective signature genes were cross-validated with different single cell analysis platform. Gene expression patterns were compared to the one before and after birth to identify signaling pathways activated in pulmonary cells. The UPR signaling pathway is one of such pathway activated during prenatal adaptation of the lung. This study appears to be an interesting resource in the field.

Reviewer comments:

Comments in the results section:

General comments:
The authors did a good job on the analysis of the single cell data and the RNA-seq data. Yet the study and the findings would strengthen with more detailed and extensive validation of the single cell results with validation of expression and cell type/location with immunofluorescence and/or FISH. In addition, the overall integration of results from single cell, bulk RNA-seq and immunofluorescence should go a step further or should be better explained. Most of the time, the

conclusions ends often with speculations without really demonstrating them clearly. While the amount of information produced is vaste, the demonstration falls always short, some information are always missing or not completely convincing (due to lack of validation). With improved validations and clearer integration of the single cell data and time course bulk data, this work could be a very useful resource in the field.

Specific comments:

Figure 1, 2: single cell analysis appears appropriate (Figures and supplemental figures 2-7). Results looks really good. Nice and clear analysis.
Figure 2: It would be nice to see the pseudotemporal ordering of the single cells along each differentiation lineage (AT1, AT2 and ciliated, club). This would complement Figure 2a, 2b, 2c and 2d. It would be also nice to show immunofluorescence (or FISH or FACS) for the Sox2hi, ciliated and club cell on section (similar to figure 2e). It would validate the markers identified (see Villani et al, Science, 2017 for validation of newly identified cell populations).
Figure 3: Validation of the identified markers using immunofluorescence or FISH would strengthen the findings and confirmed the methods for this tissue.
Figure 4: Validation of the identified markers and cell populations (MatrixFB-1 and MatrixFB-2) using immune fluorescence or FISH or FACS experiments would strengthen the findings and confirmed the methods for this tissue. The authors state an hypothesis but, unfortunately, do not try to demonstrate that Tbox genes regulates matrixFB-1 differentiation. Similarly, IGF-1 is predicted to drive the differentiation of MatrixFB-2 cells. To confirm these predictions and the validity of the method, a demonstration would be required.
The Pericyte-1 and Pericyte-2 cell populations were not validated by an orthogonal method. Immunofluorescence and/or FACS analysis methods should be used to validate the level of expression Acta2 and/or validate Pdgrfrb, Notch3, Mcam, Cspg4 and Acta2 markers.
Smooth muscle/myofibroblasts cells were also identified without orthogonal validation using immunofluorescence (or FISH or FACS) on the identified markers.
The authors identified 5 different sub-population of immune cells. Again, it would be necessary to to validate the findings with an orthogonal method. For example, markers of proliferation would demonstrate the production of proliferative lymphocytes.
Cross-validation and comparison of Drop-Seq and Fluidigm C1: The authors demonstrate that C1 data and Drop-Seq data are consistent and the results are reproducible independently of the platform used. This also validate the method used by the authors and is not really an orthogonal method to validate the pathways, gene and cell populations discovered. They conclude the paragraph with a known trade-offs between these 2 platforms. I don't think that the data presented in Figure 5 is really driving the conclusion led by the authors on the cell number/number of genes detected trade-off. More thorough studies have been done in the past to really compare the single cell platforms (see for example: Ziegenhain et al, Mol Cell, 2017; Svensson et al., 2017). This piece of information could easily be relegated to a supplementary figure.
Dynamic regulation of genes and pathways at birth (Figure 6): it is not clear what point this figure is trying to make. It would have been interesting to compare the pseudo-bulk from the single cell experiment PND1 and the bulk RNA-seq at the same time to show any concordance. In addition, the authors do not go back to the single cell data to show that these genes are seen in particular cell population (and associated function). It would have been interesting to combine the single cell results in the context of a broader time course that the bulk experiment brings. The next section seemed to be build on the integration of the single cell and the time course bulk data but it is not very clearly explained and the figures are not showing the integration clearly.
Activation of the unfolded portion response pathway at birth (Figure 7, 8): Figure7a is more information than real data and it would probably be better placed in supplemental. It would also be useful to highlight in the figure the studied gene (ATF4/6, CHOP, SYVN1, … for example). Figure 7b makes an excellent and clear point. Would it possible to add CHOP in this figure since the authors are studying it the Figure 8.
In figure 8a, the western blot appears to be slightly overexposed. Where the quantification done

on this blot? The authors would benefit to measure the band intensity on low exposure blot (i.e. not saturated signal) to prevent signal saturation and maximize the dynamic range. It would be helpful to have an immunofluorescence to show the co-localization of markers of specific cell population on section to better demonstrate that ATF6, and not ATF4, is important for ER-stress response. In Figure 7b, ATF4 and ATF6 have very similar pattern. It would be interesting to see ATF4 as well on the western blot. The pattern 47 (RNA-seq) only suggest that ATF6 is up-regulated at PND1. Next, the authors look at CHOP and Synv1. It would add to their demonstration to show on section the co-expression of ATF6, CHOP and Synv1 on sections.
Pdia3 is also regulated at PDN1 and shown to be expressed in Lymphatic endothelial cells, ciliated, broncho-pulmonary epithelial cells. The immunofluorescence shows that Pdia3 is co-localized with AT2 marker Abca3. Yet, the single cell result shows that Pdia3 is not expressed in AT2 cells only in AT1/AT2 cells. Could the authors reconcile these 2 results?
Surfactant production was shown to be up-regulated at PDN1 in the bulk RNA-seq results. It would be interesting to show also the change in expression at the protein level of different surfactant proteins. It has been shown that the ER control is important for the biosynthesis of surfactant proteins. One can wonder whether the activation of ERAD would have a direct effect on the proper biosynthesis of surfactant proteins? ERAD activation would be needed to respond to the increase production of surfactant proteins?

Comments for the methods section:
In results, the cell preparation for Drop-seq and Fluidigm C1 experiments are not described in details. These methods are significantly different in preparation and would need to be explained in better details such that it explains the dissociation methods and final concentration used to load the single cell instrument . In addition, Drop-seq sometimes required adjustment of settings and it would be very valuable to details the setting used in the experiments such as flow rates used for this experiment.

Reviewer #4:
Remarks to the Author:
This manuscript provides the first data set of single cell RNA Seq of postnatal lung cell diversity. This is an important resource for developmental biologists, as well as those interested in regeneration and disease.

1. The authors correlate their findings with current literature to provide helpful comparisons. They point out how their data are consistent with recent findings. How are the data contrasting? A few examples of how postnatal lung is not the same as adult or as stem cells would provide more perspective, not just knowing how things all match up.

2. AT2/AT1 progenitors are described. What about possible “BASCs” or cells that express markers of Club cells and alveolar cells?

3. It is noted in the discussion that the data showed potential pathways/genes that control differentiation (of endothelial cells). This can’t be revealed with only the single age analyzed. More caution should be used.

4. Is all the data from single cell (Drop or Fluidgm) or is some from bulk RNA seq?

5. The authors created a public website for others to use. When I tried the site, it wa not possible to access it.

Reviewer #1 (Remarks to the Author):

MAJOR COMMENTS:

1) In some cases, molecularly distinct sub-classes are proposed to be functionally distinct sub-types (e.g., FB1 and FB2 or Per1 and Per2) while in other cases they are proposed to be the same cell type at distinct stages of maturation (e.g., BP, Sox2-Hi, MyoFB). While these interpretations have been well-established by prior work for BPs (and somewhat for FBs), they are less well-supported by prior knowledge for the other 3 instances. The authors should provide further evidence from the literature or their own data to support the presence of two distinct pericyte sub-types. Similarly, the rationale for why they believe the MyoFB classes to represent early versus late (rather than 2 distinct sub-types) is not clearly explained.

The most surprising is the proposal that Sox2-Hi cells are the progenitors for Club and ciliated cells. This Sox2-Hi population expressed markers of both Club and ciliated cells, yet prior work in the field suggests that ciliated cells differentiate much earlier in development, and at least one lineage study using CCSP-Cre has reported that marked cells exclusively give rise to Club cells. (ciliated --> club?) Furthermore, Sox2+ airway cells that are neither Club nor ciliated cells exist in mature mouse airways and generate basal cell-like cells that become active following H1N1 influenza. In light of these previous studies, the authors' reliance on co-expression of Club and ciliated markers along with entropy analysis does not seem sufficient to conclude that the Sox2-Hi class are bipotent progenitors of Club and ciliated cells.

Author's Responses:

a. Validate pericyte-1 and pericyte-2

We applied an unsupervised and unbiased approach to identify major and sub-clusters. Cell type mapping is based on the significance and overlaps of sub-type representative signature genes with RNAs selectively expressed in a given cell type based on experimental or other high-throughput screens. In the case of pericyte 1 & 2, both expressed multiple pericyte selective markers such as *Pdgfrb*, *Notch3*, *Mcam*, and *Cspg4*. The distinction between Pericyte-1 and Pericyte-2 is that one expresses high levels of *Acta2*, another pericyte marker (also a marker for smooth muscle), while the other one expresses low levels of *Acta2* RNA. In addition, Pericyte-1 cells selectively expressed *Map3k7cl* and *Mustn1*, while Pericyte-2 cells selectively expressed *Agtr1a*, *Vsnl1,* and *Art3*. As suggested, we performed immunofluorescence staining for CSPG4, PDGFRβ and ACTA2 and demonstrated the existence of distinct PDGFRβ^{+}/CSPG4^{+} and PDGFRβ^{+}/CSPG4^{+}/ACTA2^{+} pericyte cells in mouse lung at PND1 (now added to **Supplementary Fig. 14**).

b. Why the MyoFB classes represent early versus late (rather than 2 distinct sub-types)?

We appreciate the reviewer's comment. We termed "MyoFB early" and "MyoFB late" since both express MyoFB representative genes. "MyoFB early" also enriched in stem cell markers such as *Pdgfra* and *Ednrb*. Since we do not have clear evidence that these are distinct "cell types" or related cells that are in a transitional stage, we now changed subtype terms as MyoFB-1 and MyoFB-2.

c. Are Sox2-Hi cells the progenitors for club and ciliated cells?

We cannot prove this without lineage tracing. Nevertheless, scRNA-seq is increasingly used to predict lineage and developmental relationships among heterogeneous cell populations and their differentiation states. In the present study, we applied entropy based differentiation state analysis. Our *in silicon* model predicted the potential role of Sox2-hi as progenitors for club and ciliated cells. Our *in silicon* lineage prediction is supported by previous studies. Several lines of evidence demonstrated that Sox2 plays multiple roles in both the developing and adult airways: required for maintenance and differentiation of bronchiolar club, ciliated, and goblet cells. Sox2 induces proliferation and partially reprograms alveolar epithelial cells into cells with characteristics of the conducting airways including club and ciliated cells. Conditionally deleting Sox2 leads to a reduction in the number of basal, ciliated and club cells during development and in the adult following injury[38-40].

Experimental Validation: To better address reviewer's comments, we performed immunofluorescence staining for SOX2, identifying $SOX2^{hi}$ (solid white outline) and $SOX2^{lo}$ (dotted white outline) subpopulations. Co-expression of SCGB1A1 or FOXJ1 with SOX2 was observed in the $SOX2^{hi}$**.** The result has been added to **Supplementary Fig. 8**.

2) The sc-RNAseq results indicate Lyve-1 is expressed in both lymphatic and vascular endothelial cells. However, Lyve-1 is believed to be specific for lymphatic endothelial cells based on prior work in the field. How do the authors reconcile this result? Is it possible what they call the vascular endothelial population may represent a different lymphatic endothelial cell type or progenitor?

Author's Response: Single cell approaches have enhanced resolution of cell selective markers based mRNA expression. We observed *Lyve1* RNA in both lymphatic and vascular endothelial cells in multiple single cell datasets including present data. These observations are consistent during lung development and in both human and mouse. Our single cell study suggested that *Lyve1* is **essential but not sufficient** to identify lymphatic endothelial cells. We performed immunofluorescence staining and identified co-staining of LYVE1 with EMCN and/or SOX17, the latter two are selective markers for vascular endothelial cells. Publications (Kretschmer et al., 2013 and Gordon et al., 2008) also suggested that LYVE-1 is not restricted to the lymphatic

vasculature but is also expressed on embryonic blood vessels. Our present study supports the use of Lyve-1 in combination with additional markers, such as Thy1 or Prox1, to specifically identify lymphatic endothelial cells. We have added the immunofluorescence data in **Fig. 3c**.

3) For every named cell class and sub-class, the authors should specify which exact marker(s) they use to identify the named class. For instance, what defines and distinguishes what the authors call myofibroblasts from fibroblasts, pericytes, and smooth muscle cells? To some degree, these classes express the same transcripts, though at different levels. The authors should be very clear about this to allow for comparison of their dataset and conclusions with future studies by different groups.

Author's Response: We agree with the reviewer that these mesenchymal cells share many genes; our subtype distinction is primarily based on gradient differences of selective markers. As suggested, we have added **Supplementary Table 9** listing candidate markers for each subtype that may be useful to the field.

MINOR POINTS

1) In Figure 9, the wrong color is indicated for the AGER stain

2) There are numerous instances of spelling errors throughout the manuscript

Author's Response: Yes. Thank you. We had reported to the editor regarding the mislabeling of AGER in the original Figure 9 and sent the correct version on 4/10/2018. As suggested, we have more carefully edited the manuscript to correct syntax and spelling errors.

Reviewer #2 (Remarks to the Author):

I found this a very interesting description of the transcriptional profile defining individual cell types in the mouse lung using a Drop-seq technique. The types of cell types identified and their gene expression match very well with previous knowledge but this study adds further knowledge and complexity to the variety of cell types in the lung. RNA Seq is then used to analyse temporal changes in gene expression through embryonic development and after birth, with the transcriptome data being inferred back on the single cell data to explore which cells are likely contributing to key changes in gene expression around birth. Whilst it would have been better to have single cell data at each time point that would have been a heroic effort. The key signatures of adaptation to breathing/birth include responses to oxidative and ER stress, and increased lipid metabolism which are well explained in the discussion. Protein studies are used to confirm these changes, with for example a very convincing enhancement in ATF6 at PND1 in epithelial cells. Perhaps more consideration could be given in the discussion to the need for expansion of cellular ER likely to be required to facilitate enhanced glycoprotein and lipid biosynthesis. Discussion

could also be extended as to the possible importance of the increasing expression of many of the key genes (in Fig 6d) prior to birth in preparation for the response to birth, and how that might impact in prematurity.

Author's Response: We appreciate the reviewer's positive comments and suggestions. Based on the reviewer's suggestion, we have extended the discussion section to discuss the potential role of cellular ER in glycoprotein and lipid biosynthesis, as well as the importance of increasing expression of key genes in surfactant biosynthesis, lung fluid homeostasis and birth caused oxidative stress in the critical transition of air breath at birth and the relevance to lung disease in premature infants (please see **pages 21-23**).

Minor corrections
P15 line 4 – "endothelial cell" should be "endothelial cells"
P17 line 14 – "similarly" should be "similar"
P41 line 12 "important in regulation" should be "important role in regulation"

Author's Response: We appreciate reviewer's editing. We made corresponding corrections in the revised manuscript.

For methodology, some further detail on the Drop-seq isolation procedures (currently simply referring to the original Macosko paper in Cell) that may affect gene expression (eg. disaggregation techniques, time to isolate cells) would be useful

Author's Response: We appreciate the suggestion by the reviewer. We added detailed Drop-seq isolation procedures in the revised method section.

Reviewer #3 (Remarks to the Author):

General comments:
This study appears to be an interesting resource in the field._The authors did a good job on the analysis of the single cell data and the RNA-seq data. Yet the study and the findings would strengthen with more detailed and extensive validation of the single cell results with validation of expression and cell type/location with immunofluorescence and/or FISH. In addition, the overall integration of results from single cell, bulk RNA-seq and immunofluorescence should go a step further or should be better explained. Most of the time, the conclusions ends often with speculations without really demonstrating them clearly. While the amount of information produced is vast, the demonstration falls always short, some information are always missing or not completely convincing (due to lack of validation). With improved validations and clearer

integration of the single cell data and time course bulk data, this work could be a very useful resource in the field.

Author's Response: We appreciate the reviewer's positive comments and suggestions. Based on the reviewer's suggestion, we have validated cell type selective markers and cell location using immunofluorescence and/or PLISH for endothelial, Sox2-hi, MatrixFB-2, MyoFB, Pericyte, and potential "BASCs" cells. We applied flow cytometry analyses to identify macrophages, T, and B lymphocytic immune cell populations in mouse lung at PND1. These new data are included in the **Results** section and in new **Supplementary Figures**.

Specific comments:

Reviewer #3 Figure 1, 2: single cell analysis appears appropriate (Figures and supplemental figures 2-7). Results looks really good. Nice and clear analysis. Figure 2: It would be nice to see the pseudotemporal ordering of the single cells along each differentiation lineage (AT1, AT2 and ciliated, club). This would complement Figure 2a, 2b, 2c and 2d. It would be also nice to show immunofluorescence (or FISH or FACS) for the Sox2hi, ciliated and club cell on section (similar to figure 2e). It would validate the markers identified (see Villani et al, Science, 2017 for validation of newly identified cell populations).

Author's Response: We appreciate the reviewer's positive comments and suggestions. As suggested, we performed dimension reduction using the DDRTree method in "Monocle 2"[38], and then used SLICE[37] to measure cell differentiation states and predicted entropy-directed cell differentiation trajectories. We plotted the epithelial subtype pseudotemporal lineage model ordering by the decrease of scEntropy (now added to **Fig. 2e-f** and **Supplementary Figs. 9-10**). Based on review's suggestion, we performed immunofluorescence staining for SOX2, identified $SOX2^{hi}$ (solid white outline) and $SOX2^{lo}$ (dotted white outline) subpopulations. Co-expression of SCGB1A1 or FOXJ1 with SOX2 was shown in the $SOX2^{hi}$ (now added to **Supplementary Fig. 8**).

Reviewer #3 Figure 3: Validation of the identified markers using immunofluorescence or FISH would strengthen the findings and confirmed the methods for this tissue.

Author's Response: Our main purpose here is to understand the distinct regulatory networks and driving forces regulating the two endothelial subtypes. We used our previous developed pipeline to infer the transcriptional regulatory networks and predict key transcriptional factors (TFs) for each of the two endothelial subtypes[59]. A number of TFs known to play important roles in the development of lymphatic endothelial cells (including *Prox1*[51, 58-60], *Hoxd8*[61], *Maf*[62], *Tbx1*[63], *Sox18*[64], *Elk3*[65], and *Nr2f2*[66]) and vascular endothelial cells (including *Epas1*[67], *Foxf1*[68, 69], *Klf2*[70], *Klf4*[70], *Gata2*[71], *Sox17*[43], *Ets1*[72], *Erg*[73], and *Fli1*[74]) were predicted and ranked as 20

most important TFs (**Fig. 3**), supporting the validity of our prediction method. Functional validation of the novel TFs predicted in the analysis will be processed in the future.

Reviewer #3 Figure 4: Validation of the identified markers and cell populations (MatrixFB-1 and MatrixFB-2) using immune fluorescence or FISH or FACS experiments would strengthen the findings and confirmed the methods for this tissue. The authors state an hypothesis but, unfortunately, do not try to demonstrate that Tbox genes regulates matrixFB-1 differentiation. Similarly, IGF-1 is predicted to drive the differentiation of MatrixFB-2 cells. To confirm these predictions and the validity of the method, a demonstration would be required.

Author's Response: We appreciate the reviewer's suggestions. We therefore performed immunofluorescence staining for PDGFRα$^-$ and FN1$^+$ for MatrixFB-1, SFRP2$^+$ and IGFBP5$^+$ for MatrixFB-2. We identified a subset of MatrixFB-2 (SFRP2$^+$/IGFBP5$^+$) cells within the mesenchymal compartment lining the proximal airways (now added to **Fig. 4c**). The SFRP2$^+$ and/or IGFBP5$^+$ cell population was distinct from cells expressing ACTA2 and TGFBI (now added to **Supplementary Fig. 12**), the latter is predicted as a new marker for MyoFB and smooth muscle cells (now added to **Supplementary Fig. 13**). While most of the research papers focusing on one cell lineage or cell heterogeneity within the same lineage, we have predicted 20 distinct subtypes, predicted potential key regulators and associated signaling networks for multiple cell types. It is beyond the scope to firmly confirm all of these findings in one paper. We are interested to follow up the distinct signaling network influencing MatrixFB-1 and MatrixFB-2 differentiation in a separate study.

Reviewer #3: The Pericyte-1 and Pericyte-2 cell populations were not validated by an orthogonal method. Immunofluorescence and/or FACS analysis methods should be used to validate the level of expression Acta2 and/or validate Pdgrfrb, Notch3, Mcam, Cspg4 and Acta2 markers.

Author's Response: We appreciate the reviewer's suggestion. We therefore tested immunofluorescence staining for CSPG4, PDGFRβ and ACTA2, demonstrating the presence of PDGFRβ$^+$/CSPG4$^+$ and PDGFRβ$^+$/CSPG4$^+$/ACTA2$^+$ pericyte cells in mouse lung at PND1 (now added to **Supplementary Fig. 14**).

Reviewer #3: Smooth muscle/myofibroblasts cells were also identified without orthogonal validation using immunofluorescence (or FISH or FACS) on the identified markers.

Author's Response: We appreciate the reviewer's suggestion. We preformed immunofluorescence staining for αSMA (ACTA2) in PDGFRα-GFP$^+$ fibroblast and demonstrated the presence of PDGFRα$^+$/ACTA2$^-$ (MyoFB-1), PDGFRα$^+$/ACTA2$^+$ (MyoFB-2) and PDGFRα$^-$/ACTA2$^+$ (smooth muscle) populations (now added to **Supplementary Fig. 11**).

In addition, we tested a new marker, *Tgfbi*, which identified all three subtypes of smooth muscle/myofibroblasts cells (now added to **Supplementary Fig. 13**).

Reviewer #3: The authors identified 5 different sub-population of immune cells. Again, it would be necessary to validate the findings with an orthogonal method. For example, markers of proliferation would demonstrate the production of proliferative lymphocytes.

Author's Response: We appreciate the reviewer's suggestion. We therefore used flow cytometry to further identify macrophages, T cells, and B cells populations in mouse lung at PND1 (now added to **Supplementary Fig. 15**).

Reviewer #3: Cross-validation and comparison of Drop-Seq and Fluidigm C1: The authors demonstrate that C1 data and Drop-Seq data are consistent and the results are reproducible independently of the platform used. This also validate the method used by the authors and is not really an orthogonal method to validate the pathways, gene and cell populations discovered. They conclude the paragraph with a known trade-offs between these 2 platforms. I don't think that the data presented in Figure 5 is really driving the conclusion led by the authors on the cell number/number of genes detected trade-off. More thorough studies have been done in the past to really compare the single cell platforms (see for example: Ziegenhain et al, Mol Cell, 2017; Svensson et al., 2017). This piece of information could easily be relegated to a supplementary figure.

Author's Response: We appreciate the reviewer's suggestions. We referenced Ziegenhain et al, Mol Cell, 2017; Svensson et al., 2017 and we moved original **Fig. 5** to **Supplementary Fig. 16**. Our comparison of the two platforms aligned well with previous findings and supported the conclusion that increasing cell numbers increases statistical power to identify more cell types while deeper sequencing leads to more mapped genes.

Reviewer #3: Dynamic regulation of genes and pathways at birth (Figure 6): it is not clear what point this figure is trying to make. It would have been interesting to compare the pseudo-bulk from the single cell experiment PND1 and the bulk RNA-seq at the same time to show any concordance. In addition, the authors do not go back to the single cell data to show that these genes are seen in particular cell population (and associated function). It would have been interesting to combine the single cell results in the context of a broader time course that the bulk experiment brings. The next section seemed to be build on the integration of the single cell and the time course bulk data but it is not very clearly explained and the figures are not showing the integration clearly.

Author's Response: We sought to identify pathways and major bioprocesses selectively induced in PND1 (representing the physiological adaptive responses to the transition to air breathing) via bulk RNA-seq time course analysis. We then used single cell data to reveal the cell specific UPR responses which we validated using immunofluorescence and western blot. Based on the reviewer's suggestion, we have created pseudo-bulk RNA-seq samples from Drop-seq based scRNA-seq data and Fluidigm C1 based scRNA-seq data from mouse lung at PND1 and compared these with bulk RNA-seq time course RNA-seq data. t-SNE analysis of pseudo-bulk and bulk RNA-seq samples showed that the pseudo-bulk and bulk RNA-seq samples from PND1 were closely related, suggesting the concordance of pseudo-bulk and bulk RNA-seq data from PND1. The results are now added to **Supplementary Fig. 22**.

Reviewer #3: Activation of the unfolded portion response pathway at birth (Figure 7, 8): Figure7a is more information than real data and it would probably be better placed in supplemental. It would also be useful to highlight in the figure the studied gene (ATF4/6, CHOP, SYVN1, … for example). Figure 7b makes an excellent and clear point. Would it possible to add CHOP in this figure since the authors are studying it the Figure 8.

Author's Response: We appreciate the reviewer's suggestion. Based on the reviewer's suggestion, we have now moved original **Fig. 7a** to **Supplementary Fig. 18** and highlighted the studied genes in the same Figure. We changed original **Fig. 7b** to **Fig. 6a** and added CHOP (*Ddit3*) to the gene panel. We moved original **Fig. 8** to **Fig. 6b-e**.

Reviewer #3 : In figure 8a, the western blot appears to be slightly overexposed. Where the quantification done on this blot? The authors would benefit to measure the band intensity on low exposure blot (i.e. not saturated signal) to prevent signal saturation and maximize the dynamic range. It would be helpful to have an immunofluorescence to show the co-localization of markers of specific cell population on section to better demonstrate that ATF6, and not ATF4, is important for ER-stress response. In Figure 7b, ATF4 and ATF6 have very similar pattern. It would be interesting to see ATF4 as well on the western blot. The pattern 47 (RNA-seq) only suggest that ATF6 is up-regulated at PND1. Next, the authors look at CHOP and Synv1. It would add to their demonstration to show on section the co-expression of ATF6, CHOP and Synv1 on sections.

Author's Response: We agree with review that ATF4 and ATF6 have similar expression patterns in lung epithelial cell subtypes. The difference is that ATF6 induced at birth while ATF4 expression was not changed. These results are consistent from RNA-seq and HE staining (original **Fig. 7a**, **Fig. 8**, and **Supplementary Fig. 8**). Consistent with this observation, ATF4 transcriptional targets were not induced at birth as well (see original **Fig 7a,** now **Supplementary Fig. 18**). Therefore we did not prioritize ATF4 for further confirmation. We

also tested co-staining of ATF6, SYVN1 and CHOP in immunofluorescence, but the antibodies did not work, we have no conclusive data to support co-expression of these three proteins.

Reviewer #3: Pdia3 is also regulated at PDN1 and shown to be expressed in Lymphatic endothelial cells, ciliated, broncho-pulmonary epithelial cells. The immunofluorescence shows that Pdia3 is co-localized with AT2 marker Abca3. Yet, the single cell result shows that Pdia3 is not expressed in AT2 cells only in AT1/AT2 cells. Could the authors reconcile these 2 results?

Author's Response: Single cell analysis predicted enriched expression of *Pdia3* in Lymphatic endothelial cells, airway epithelial cells and AT1/AT2 cells. We do not conclude that *Pdia3* is not expressed in AT2 cells. Among epithelial cell populations, the total number of *Pdia3*+ cells was highest in AT2 cell population; however, *Pdia3* was less enriched in AT2 cells compared with other epithelial cells (see attached tables below). *Nkx2-1* was most significantly enriched in AT1/AT2 cells. Immunofluorescence staining demonstrated that PDIA3 co-localized with NKX2-1, consistent with single cell RNA-seq data.

Epi-subtypes	*Pdia3+*	*Nkx2-1+*	Co-expressed	Total cell#
AT1/AT2	237	369	208	460
AT2	268	503	188	827
AT1	122	219	86	395
Sox2hi	39	31	30	46
Ciliated	25	22	17	40
Club	22	20	14	41

Gene	Epi subtypes	Cluster Expressed	Cluster Frequency	Cluster Recall	Sensitivity based Enrichment	Fisher's exact p value
Nkx2-1	AT1/AT2	369	0.80	0.29	5.04	2.21E-186
Nkx2-1	AT2	503	0.61	0.40	3.87	1.14E-161
Nkx2-1	AT1	219	0.55	0.17	3.44	9.92E-67
Nkx2-1	Sox2hi	31	0.67	0.02	3.51	1.34E-14
Nkx2-1	Ciliated	22	0.55	0.02	4.00	2.23E-08
Nkx2-1	Club	20	0.49	0.02	3.92	1.23E-06
Pdia3	AT1/AT2	237	0.52	0.09	1.57	3.16E-12
Pdia3	Sox2hi	39	0.85	0.01	1.75	7.16E-12
Pdia3	Ciliated	25	0.62	0.01	2.00	4.21E-04
Pdia3	Club	22	0.54	0.01	1.96	1.22E-02
Pdia3	AT2	268	0.32	0.10	0.97	0.95
Pdia3	AT1	122	0.31	0.04	0.81	0.97

To further validate the Pdia3 expression in AT1 and AT2 cells, we repeated immunofluorescence staining for PDIA3 and ABCA3 (an AT2 cell marker) and PDPN (another AT1 marker) on frozen lung sections at PND1. We observed clear co-localization of PDIA3 with ABCA3, and lack of co-staining with AGER (original **Fig. 9,** now **Fig. 7** in the revised manuscript) or PDPN (**Supplementary Fig. 20**). Previous proteomics study of cultured AT2 cells revealed the induction of PDIA3 protein expression during AT2 to AT1 transdifferentiation and the role of PDIA3 is mediated by WNT signaling (PMID: 26035385), consisting with the present finding that both *Pdia3* and *Axin2* are more enriched in AT1/AT2 cells than other epithelial cell types. The lack of co-expression with AT1 markers is likely relative to the plasticity of alveolar AT1 and AT2 cells at PND1 and immunofluorescence staining fails to capture this transition stage of cells.

Reviewer #3: Surfactant production was shown to be up-regulated at PDN1 in the bulk RNA-seq results. It would be interesting to show also the change in expression at the protein level of different surfactant proteins. It has been shown that the ER control is important for the biosynthesis of surfactant proteins. One can wonder whether the activation of ERAD would have a direct effect on the proper biosynthesis of surfactant proteins? ERAD activation would be needed to respond to the increase production of surfactant proteins?

Author's Response: We appreciate the reviewer's suggestion. We collected lung developmental proteomics data from https://www.lungmap.net/. We plotted the profiles of surfactant proteins (see attached plot below). The levels of Sftpa1, Sftpb, Sftpd and Napsa correlated well with their RNA expression (**Fig. 5**). Sftpc, Lpcat1 and Abca3 were not detected in the protein dataset.

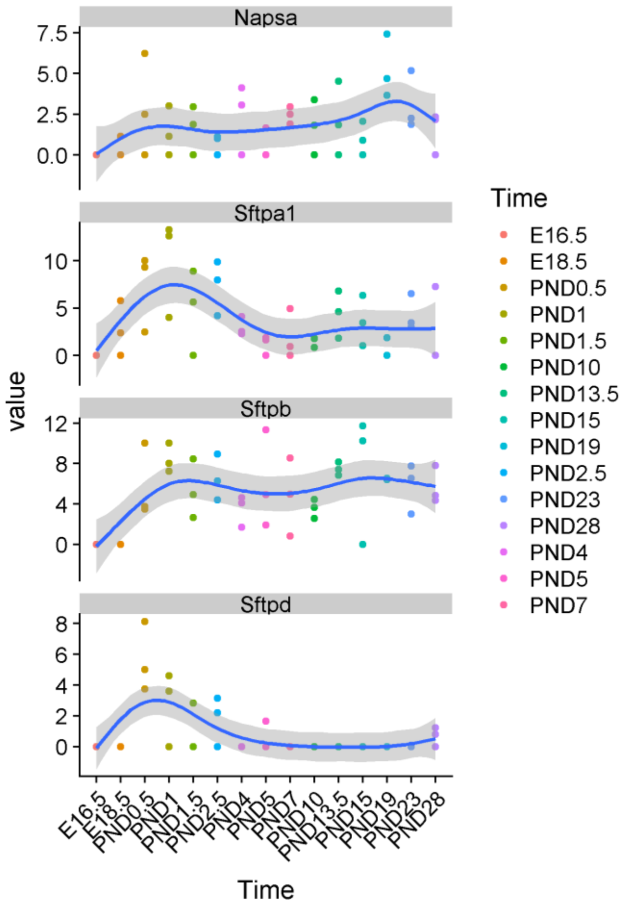

Reviewer #3

Comments for the methods section:

In results, the cell preparation for Drop-seq and Fluidigm C1 experiments are not described in details. These methods are significantly different in preparation and would need to be explained in better details such that it explains the dissociation methods and final concentration used to load the single cell instrument. In addition, Drop-seq sometimes required adjustment of settings and it would be very valuable to details the setting used in the experiments such as flow rates used for this experiment.

Author's Response: We appreciate the suggestion by the reviewer. We added detailed Drop-seq and Fluidigm C1 experimental procedures in the revised **Method** section.

Reviewer #4 (Remarks to the Author):

This manuscript provides the first data set of single cell RNA Seq of postnatal lung cell diversity. This is an important resource for developmental biologists, as well as those interested in regeneration and disease.

1. The authors correlate their findings with current literature to provide helpful comparisons. They point out how their data are consistent with recent findings. How are the data contrasting? A few examples of how postnatal lung is not the same as adult or as stem cells would provide more perspective, not just knowing how things all match up.

Author's Response: We appreciate the suggestion by the reviewer. We have pointed out differences and novelty when comparing with previous findings and in the discussion section. We have been active in organizing lung development and disease related single cell data from other laboratories and publications in our website (https://research.cchmc.org/pbge/lunggens/mainportal.html), seeking to merge external data in our database to enhance availability/access and integrative analyses. We believe this will enable detailed comparisons to reveal the differences and similarities of related studies that we hope will be useful for the research community.

2. AT2/AT1 progenitors are described. What about possible "BASCs" or cells that express markers of Club cells and alveolar cells?

Author's Response: We appreciate the suggestion by the reviewer. We preformed PLISH seeking to identify potential "Broncho-Alveolar stem cells (BASCs)" at PND1, identifying cells co-expressing *Sftpc*, *Ager*, and *Scgb1a1*. We were able to identify only 6 potential "BASCs" like cells in 3 PND1 mice, representing less than 0.1% of the total lung cells as counted by DAPI staining. We did not confidently detect any such cells in E18.5 mouse lungs (now added to **Supplementary Fig. 7c** and **7d**). We also checked "BASCs" like cells using our PND1 drop-seq data, with the criteria of zscore>1, 20 out of 1809 epithelial cells were identified as triple positive (*Sftpc+Ager+Scgb1a1+*), accounting for 1% of epithelial and 0.2% of total lung cells. These cells clustered best with AT1/AT2 cells (now added to **Supplementary Fig. 7c**)

3. It is noted in the discussion that the data showed potential pathways/genes that control differentiation (of endothelial cells). This can't be revealed with only the single age analyzed. More caution should be used.

Author's Response: We are not sure what "potential pathways/genes that control endothelial cells differentiation" that reviewer refers to. For the two subtypes of endothelial cells, we used our previously developed SINCERA pipeline to predict the transcriptional regulatory networks

and key transcription factors (TFs) for each of the proposed two endothelial subtypes. A number of TFs known to play important roles in the development of lymphatic endothelial cells (including *Prox1*[51, 58-60], *Hoxd8*[61], *Maf*[62], *Tbx1*[63], *Sox18*[64], *Elk3*[65], and *Nr2f2*[66]) and vascular endothelial cells (including *Epas1*[67], *Foxf1*[68, 69], *Klf2*[70], *Klf4*[70], *Gata2*[71], *Sox17*[43], *Ets1*[72], *Erg*[73], and *Fli1*[74]) (**Fig. 3**) were identified, supporting the validity of our prediction method.

4. Is all the data from single cell (Drop or Fluidgm) or is some from bulk RNA seq?

Author's Response: Our paper is primarily focused on the drop-seq analyses from PND1. Fluidigm C1 data was used for cross-validation and for comparison of Drop-Seq. We used bulk RNA-seq data from mouse lungs at E16.5, E18.5, PND1, PND3, PND7, PND14, and PND28 to identify temporal changes in gene expression through embryonic development and after birth.

5. The authors created a public website for others to use. When I tried the site, it was not possible to access it.

Author's Response: We checked the website functionality to assure the correct functioning of the web site. Please see **https://research.cchmc.org/pbge/lunggens/SCLAB.html.**

Reviewers' Comments:

Reviewer #1:
Remarks to the Author:
In their revised manuscript, Guo and colleagues have adequately addressed my concerns raised with the original submission. They have improved their validation of putative distinct cell classes by immunostaining for distinguishing markers in intact lung. They have also more clearly presented the distinguishing markers for each putative cell sub-type. Overall, the manuscript is much improved and I have no remaining criticisms. In its current state, this manuscript is a valuable addition to the lung biology field.

Reviewer #2:
Remarks to the Author:
The authors have addressed my comments and appear to have also satisfied the comments and requests of the other reviewers, and the manuscript is considerably improved. Although the two very recent papers in Nature (refs 121 and 122) have been included, perhaps a more detailed comparison of alignment of findings could have been provided.

Reviewer #3:
None

Reviewer #4:
Remarks to the Author:
The authors have clearly done a lot of work - the additional analysis of the single cell data is helpful, especially since they added all the comparison among the two batches. Overall the value of these data as a resource outweighs the need for complete follow up, yet questions remain when the authors apply some cell biology.

For example, the Sox2 staining is very weak and I am not sure if cells can be decided among low and high Sox2.

It's unclear to me why they look at Ager/Spc/Ccsp overlap when they look for BASCs. Why not just Spc and Ccsp? Also when they do PLISH, they are not looking at terminal bronchioles - where BASCs should be.

How can we be sure that high level of entropy means higher progenitor? It might just be describing a transitional state. The authors should state better that in their conclusion inferred from the single cells data it's just an hypothesis that needs testing with lineage tracing.

- Lyve1 t-sne is Sopp Fig 4d is quite different in intensity with the one shown in Fig 3B.

REVIEWERS' COMMENTS:

Reviewer #4 (Remarks to the Author):

The authors have clearly done a lot of work - the additional analysis of the single cell data is helpful, especially since they added all the comparison among the two batches. Overall the value of these data as a resource outweighs the need for complete follow up, yet questions remain when the authors apply some cell biology.

Author's response: thank you for the positive comments.

For example, the Sox2 staining is very weak and I am not sure if cells can be decided among low and high Sox2.

Author's response: we agree that the SOX2 immunofluorescence staining is not quantifiable to distinguish cell subtypes. We have deleted Supp. Fig 8 from the manuscript.

It's unclear to me why they look at Ager/Spc/Ccsp overlap when they look for BASCs. Why not just Spc and Ccsp? Also when they do PLISH, they are not looking at terminal bronchioles - where BASCs should be.

Author's response: Our drop-seq analysis doesn't identify a clear cluster of cells co-expressing *Scgb1a1* and *Sftpc*. To address Reviewer's request, we preformed PLISH analyses and identified cells co-expressing *Sftpc* and *Scgb1a1* (Supplementary Fig. 7c, d). We reviewed multiple sections to identify these cells previously described as BASCs. We did not find co-expressing cells in bronchoalveolar duct junction regions. We therefore cannot determine if they represent BASCs. We report them as "epithelial cells co-expressing *Sftpc* and *Scgb1a1* and will not refer them as BASCs. Based on PLISH and drop-seq data, we also detected a small percentage of cells co-expressing *Sftpc*, *Ager*, and *Scgb1a1* (Supplementary Fig. 7), these cells were clustered in the AT1/AT2 epithelial subtype.

How can we be sure that high level of entropy means higher progenitor? It might just be describing a transitional state. The authors should state better that in their conclusion inferred from the single cells data it's just an hypothesis that needs testing with lineage tracing.

Author's response: we agree with the reviewer that entropy is used to predict the order of cell differentiation stages or transitional state but not to determine which cells are the actual progenitors. We tested the algorithm for multiple datasets with known lineage or developmental time course, the predictions are consistent with the experimental findings (Guo, et al 2017). We agree that *in silico* prediction methods including SLICE and Monocle (order cells by pseudo-time) provide statistical probability of trajectories of cell states, not directly identify "cell type" or progenitors. We clarified this in the text.

- Lyve1 t-sne is Sopp Fig 4d is quite different in intensity with the one shown in Fig 3B.

Author's response: we appreciate reviewer's observation. In the main figure, we used z-score normalized data and our customized plot function based on ggplot2 package. The supplementary figure was plotted using the FeaturePlot function in Seurat package with default settings (log2 transformed values). We replot the supplementary figures 2-6 using same parameters to be consistent.
